# The Burden of HPV Infections and HPV‐Related Diseases Among People With HIV: A Systematic Literature Review

**DOI:** 10.1002/jmv.70274

**Published:** 2025-04-02

**Authors:** Bekana K. Tadese, Xuedan You, Tidiane Ndao, Joseph E. Tota, Ya‐Ting Chen, Alisa Chowdhary, Jia Pan, Ana C. Costa, Nelly Mugo

**Affiliations:** ^1^ Merck & Co., Inc. Rahway New Jersey USA; ^2^ Adelphi Values PROVE™, Adelphi Mill Bollington UK; ^3^ Kenya Medical Research Institute Nairobi City Kenya

**Keywords:** cervical cancer, HIV, HPV, HPV‐related cancer, human papillomavirus, PWH

## Abstract

Human papillomavirus (HPV) is associated with a significant global burden of precancerous lesions and cancer. People with HIV (PWH) are at higher risk of HPV infection and HPV‐related diseases. This systematic review was conducted to synthesize data on the burden of HPV infection and HPV‐related diseases among PWH. Studies published between January 2018–June 2023 were sourced from databases and conferences. Included were 221 publications containing epidemiological data on HPV infections and the clinical burden of HPV‐related diseases among PWH. The burden varied by geographical region, age, sex, and sexual orientation. Compared to people without HIV (PWoH), PWH had higher prevalence and incidence of HPV infection and HPV‐related diseases. Among PWH, the prevalence of anal HPV infection ranged between 44% and 83%; men had a higher prevalence and incidence of anogenital warts than women. The incidence of anal HPV infection was over two‐fold greater among transgender women with HIV and men who have sex with men with HIV than among their respective counterparts without HIV. Incident HPV‐related anal cancer was up to two‐fold higher among PWH than PWoH, and incident cervical cancer was up to six times higher among women with HIV than those without. The most prevalent high‐risk (hr) HPV genotypes with HPV‐related disease were vaccine genotype HPV16/18/52/58. HPV35 was one of the most prevalent genotypes with anal or cervical HPV infection among PWH of African descent. PWH also have a higher burden of concurrent HPV infections and HPV‐related diseases. This study calls for strengthening appropriate HPV vaccine delivery and increasing vaccine uptake among this high‐risk group, potentially by integrating HPV vaccination with routine HIV care.

## 1 Introduction

Human papillomavirus (HPV) is the most common sexually transmitted infection, and is associated with a significant burden of precancerous lesions and cancers globally [1, 2]. A prominent example is cervical cancer, the fourth most frequently diagnosed and the fourth leading cause of cancer death among women worldwide. Over 90% of global cervical cancer cases are caused by HPV.

There are > 200 HPV genotypes; however, about 15 of them are classified as high‐risk (hr) genotypes, and responsible for HPV‐related cancers [3, 4, 5]. Particularly, persistent HPV infections cause almost all cervical cancers as well as contribute to a substantial burden of anogenital and oropharyngeal cancers [6]. Moreover, HPV‐related cancers contribute to high morbidity and mortality rates and decreased quality of life of patients [7, 8]. HPV‐related diseases are also associated with high healthcare resource utilization and costs [7]. One study estimated that lifetime medical treatment costs in the United States (US) alone amount to $744 million [7, 8].

While HPV affects the general population, it poses unique challenges for people with human immunodeficiency virus (PWH) due to immune system dysfunction [1]. Globally, PWH were estimated over 39 million at the end of 2022 [9]. Although it is recognized that HPV infection and HPV‐related diseases among PWH represent a substantial healthcare burden globally, the evidence base communicating this burden is limited.

Understanding the burden of HPV in PWH is crucial for the implementation of effective prevention strategies, including HPV vaccination and optimizing clinical management including improvements of screening. This systematic review provides an understanding of the burden of HPV infections and HPV‐related diseases among PWH and identified remaining research gaps.

## 2 Methods

This is a systematic literature review that was performed following the Preferred Reporting Items for Systematic Reviews and Meta‐Analyses (PRISMA) guidelines [10].

### 2.1 Search Strategy and Inclusion Criteria

A comprehensive search was conducted within the Ovid platform and included EMBASE, Medline, and Evidence‐Based Medicine Reviews (including Cochrane databases). Search strategies and supplementary searches of relevant conference proceedings with the full list of the conferences were included in Supplementary Table 1 (Supporting Information S1: Table S1). Publications reporting on epidemiological, clinical, humanistic, and economic outcomes among adults ( ≥ 18 years) with HIV with HPV infections or HPV‐related diseases were included. Randomized controlled trials, case studies or case reports, and review articles were excluded. Full eligibility criteria are in Supporting Information S1: Table S2.

The search was limited to publications from January 1, 2012, to June 5, 2023. Given the wealth of evidence identified following full‐text review, the time horizon for eligibility was shortened to publications from 2018 to 2023 for most recent and relevant outcomes, by excluding publications before 2018 from the analysis. Search strategies are presented in Supporting Information S1: Tables S3–S5. The main study outcomes of interest included the prevalence and incidence of HPV infection and HPV‐related diseases at different anatomical sites such as cervical, anogenital, oral, and other anatomical sites. In addition, the review captured study characteristics including age, gender, sexual orientation, and region.

### 2.2 Data Collection Procedure

Abstract and full‐text screening were conducted by two independent reviewers, with any differences resolved by a third reviewer. Data extraction was conducted by one reviewer and verified by a second reviewer, using a standardized data extraction form designed to capture study characteristics, methods, study population, subgroup characteristics, and reported outcomes. Assessment of study quality was conducted using Joanna Briggs Institute (JBI) Critical Appraisal Tools [11]. Assessment scales used according to study type are in Supporting Information S1: Figures S1–S3 and critical assessment is in Supporting Information S1: Figure S4.

### 2.3 Data Synthesis

Data captured from studies were synthesized narratively. The primary outcomes summarized and reported includes prevalence and incidence of HPV infections at different anatomical sites, and HPV‐related disease outcomes including cervical, anogenital, oral, and other anatomical sites related diseases. The synthesized prevalence and incidence of HPV infection and HPV‐related outcomes were presented by figures for visualization of data. Values from studies reporting on the same outcome type were used to calculate means or ranges provided within this review. When a study had anomalous values in comparison to the rest of the data set, the weighted mean was calculated based on study sample sizes to mitigate the effect of the anomalous value on the final mean.

## 3 Results

### 3.1 Study Selection and Population Characteristics

**Figure 1:**
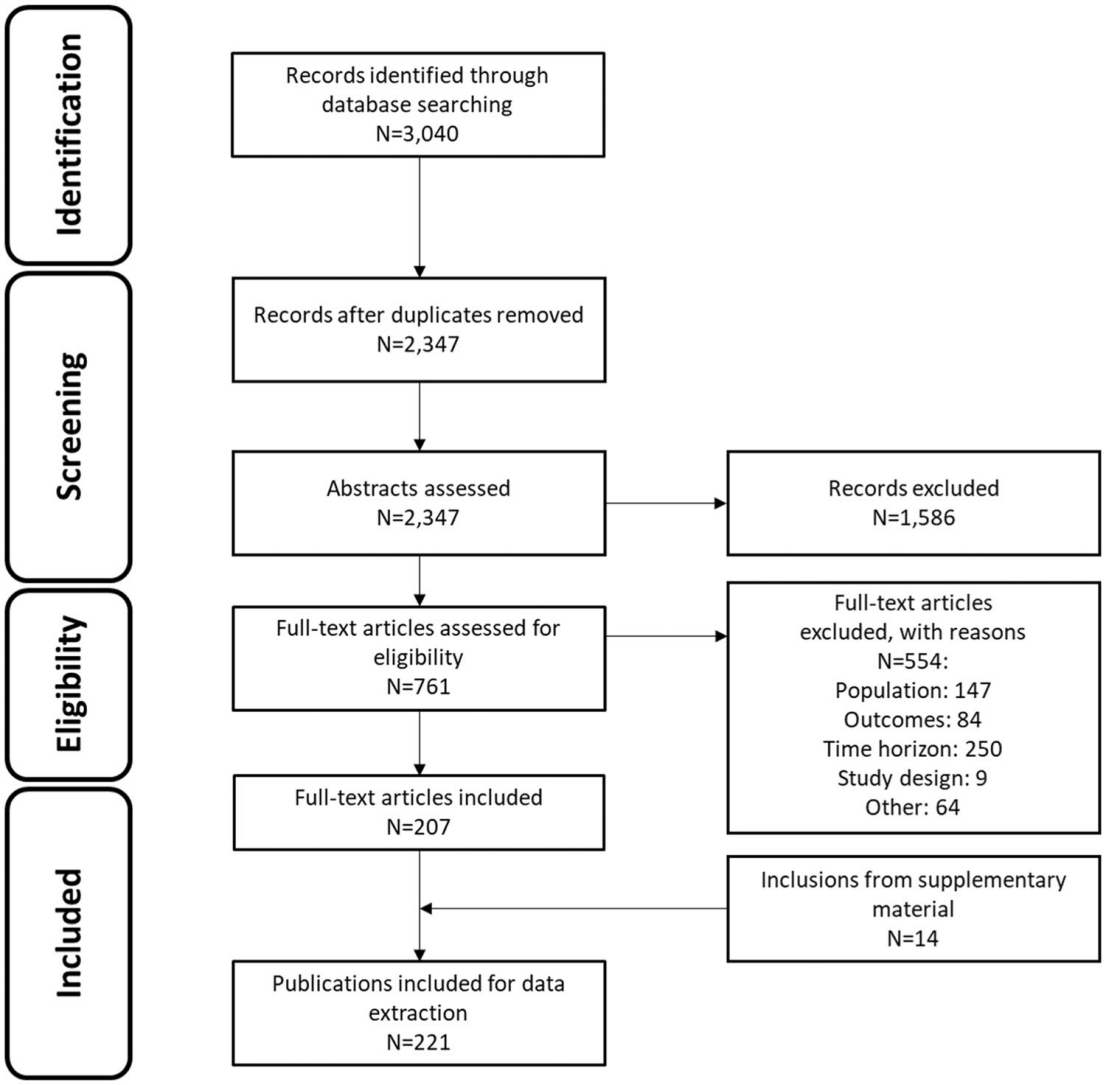
PRISMA flow diagram of included publications retrieved from databases and conferences.

A total of 221 publications were included (Figure 1), reporting on data collected in Africa (*n* = 65), Europe (*n* = 49), the US and Canada (*n* = 40), Asia (*n* = 33), Central and South America (*n* = 23) and the Middle East (*n* = 1). Although this review primarily focused on studies that reported HPV burden in PWH, a subset of comparative studies (*n* = 109) that compared PWH versus PWoH were also included in this report.

Several studies have also reported on subgroups, such as women with HIV (WWH), and men who have sex with men (MSM). Full details of included publications can be found within the supplementary materials.

### 3.2 Epidemiology of HPV Infections

Overall, 184 publications included prevalence data on HPV infections, of which, six included multinational data. Cervical (*n* = 100 publications), anal (*n* = 93), and oral (*n* = 38) anatomical sites were the most reported on (Supporting Information S1: Figure S5). Publications reporting on the epidemiology of HPV infection at anogenital sites beyond anal and cervical (including penile, vulval and vaginal) were captured and included in this review, however not reported on separately here due to heterogenous and insufficient data, limiting the synthesis of these results. There was a lack of recent incidence data, with only 20 publications reporting on data ranging between 1996 and 2019.

**Figure 2:**
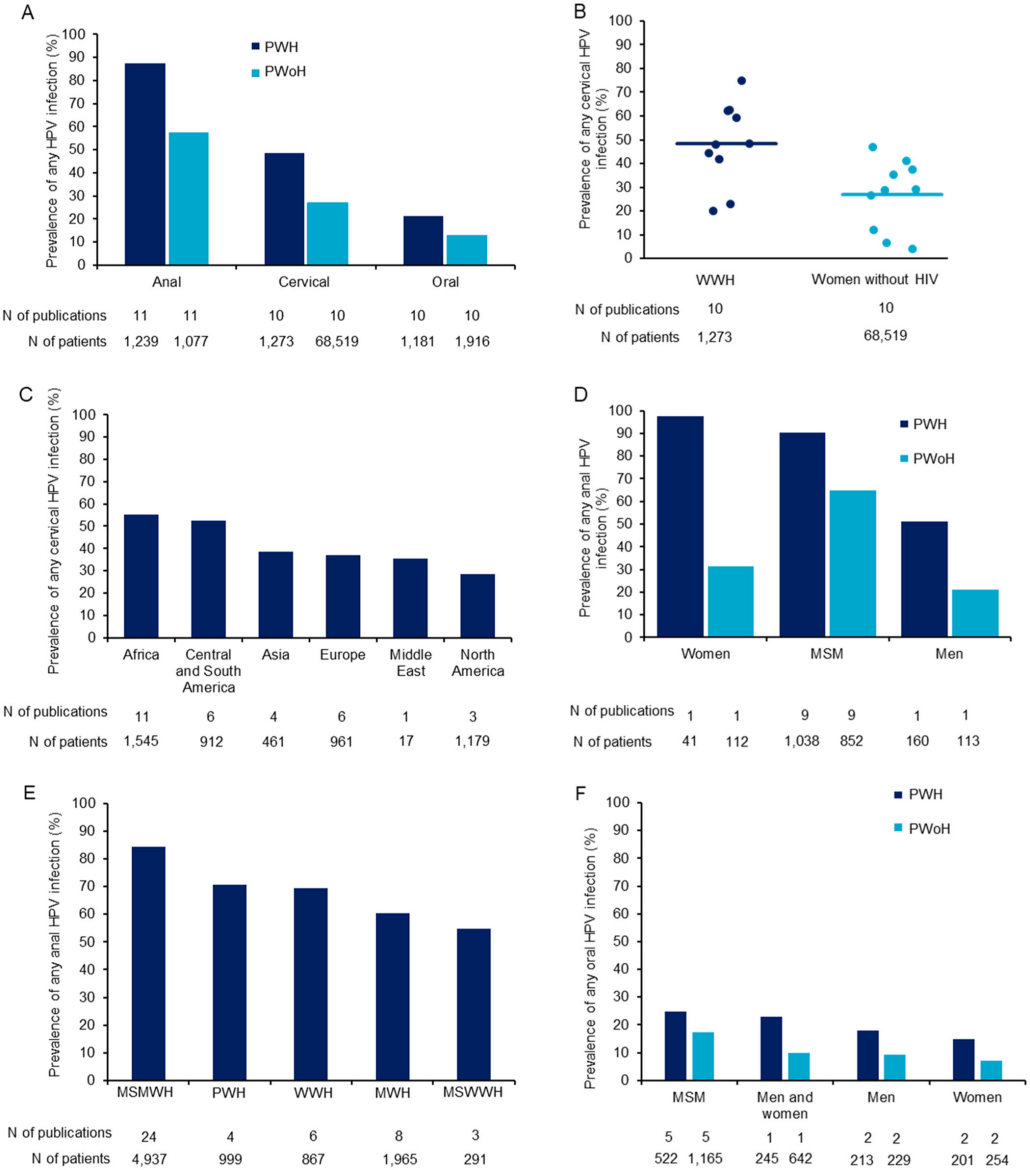
(A) Summary of HPV prevalence across anal, cervical and oral sites by HIV status. Includes comparative publications only [12, 13, 14, 15, 16, 17, 18, 19, 20, 21, 22, 23, 24, 25, 26, 27, 28, 29, 30, 31, 32, 33, 34]. Publications reporting on multiple subpopulations have been included as separate data points. (B) Average prevalence of cervical HPV infection from publications reporting comparative data [12, 13, 14, 25, 26, 27, 28, 29, 30, 31]. (C) Average weighted prevalence of cervical HPV infection among WWH, stratified by geographic region [12, 13, 14, 25, 26, 27, 28, 29, 35, 36, 37, 38, 39, 40, 41, 42, 43, 44, 45, 46, 47, 48, 49, 50, 51, 52, 53, 54, 55]. Weighted prevalence was calculated according to the total sample size of the population from each region. (D) Average prevalence of anal HPV infection, stratified by patient population and HIV status; reporting only from publications with comparative data [20, 21, 22, 56]. (E) Average prevalence of anal HPV infection stratified by subpopulation [15, 16, 17, 18, 19, 20, 21, 22, 23, 24, 52, 54, 56, 57, 58, 59, 60, 61, 62, 63, 64, 65, 66, 67, 68, 69, 70, 71, 72, 73, 74, 75, 76, 77, 78]. (F) Summary of any oral HPV infection, stratified by patient population and HIV status [16, 17, 19, 31, 32, 33, 34, 79]. HIV, human immunodeficiency virus; HPV, human papillomavirus; MWH, men with HIV; MSM, Men who have sex with men; MSMWH, men who have sex with men with HIV; MSW, Men who have sex with women; MSWWH, men who have sex with women with HIV; N, number; PWH, people with HIV; PWoH, people without HIV; WWH, women with HIV.

Generally, the prevalence and incidence of anal, cervical, and oral HPV infections are consistently higher in PWH than PWoH (Figure 2A). In addition, hrHPV types and concurrent infection with multiple HPV genotypes were more common in PWH than PWoH (supplementary tables).

### 3.3 Cervical HPV Infections

Overall, 89 publications investigated cervical HPV prevalence, of which 10 included comparative data (Supporting Information S1: Tables S6 and S7). The prevalence of cervical HPV infections is substantially higher in WWH (Figure 2B), ranging between 20% and 98%, and with reports of up to six times higher prevalence than in women without HIV [12, 13]. The wide range is potentially due to geographical differences and age. HPV infection was on average approximately two times more prevalent in African than North American WWH (Figure 2C). The lowest prevalence of 20.2% was from the US, based on medical records data from 68,096 women aged 30–64 years, of which 608 were WWH [14]. This prevalence was significantly higher (*p* < 0.001) than that in women without HIV (6.5%) [14]. The highest prevalence of infection with one or more HPV type was 98.4%, reported from Kenya among 245 pregnant WWH aged 17–42 years and 85.5% carried at least one hrHPV type [35].

The reported prevalence and incidence of cervical HPV infection also varied with age across studies. Two publications reported that African women aged 25–39 years have a higher prevalence of cervical HPV infection compared to older women [80, 81], and another from Morocco found the highest prevalence in women aged 30–40 years [82]. Teixeira et al. (2018) reported that the prevalence of hrHPV was higher in Brazilian women (*N* = 299) aged ≥ 36 years and peaked in women ≥ 50 years [83]. While there were limited age‐stratified incidence data on cervical HPV infection (Supporting Information S1: Table S8), a study from Tanzania reported that incidence of cervical hrHPV infection was higher among WWH aged 25–29 years and lowest in 50–60 years old; both WWH and women without HIV had decreasing HPV incidence rates with increasing age [84]. While incidence was higher among WWH than women without HIV in all age groups, the smallest difference in incidence rate was seen among women aged 25–29 years and the largest difference in the incidence rate was among women 50–60 years [84].

### 3.4 Anal HPV Infection

Studies comparing the prevalence and incidence of anal HPV infection in PWH and PWoH were sparse, except in the MSM population (Supporting Information S1: Tables S9–S12). The available data shows that the prevalence of anal HPV infection among PWH varied across subpopulations. Anal HPV prevalence was three times higher in WWH than in women without HIV, and over two‐fold higher in men with HIV (MWH) than men without HIV. MSM with HIV had a prevalence of anal HPV infection 1.4 times higher than MSM without HIV (Figure 2D). MSM with HIV had the highest prevalence whereas men with HIV who have sex with women the lowest (Figure 2E). Therefore, publications that report anal HPV infection data in PWH varied by the proportion of these subgroups included in the studies.

Regional analysis revealed that although MSM with HIV have comparable high prevalences across all geographical regions, those in Europe have the highest prevalence of any anal HPV infection with prevalence rates up to 91% [15, 16, 17, 18, 19, 57, 58, 59, 60, 61, 62, 63, 64], compared with Central and South America (86.1%) [85], North America (83.5%) [65, 86], Africa (83.3%) [20, 21, 22, 87], Asia (77.8%) [23, 66, 67, 68], and Oceania (74.0%) [69].

As with anal HPV infection prevalence, the incidence is high among MSM with HIV (Supporting Information S1: Table S13) [69, 88, 89]. One US study found the incidence rate of anal infections with hrHPV genotypes covered by the nonavalent vaccine (HPV16/18/31/33/45/52/58) in MSM with HIV was about two times higher than in MSW with HIV (15.6 vs. 7.6 per 100 person‐years) [90]. Furthermore, the incidence of anal HPV infection was over two‐fold greater in men and transgender women with HIV than their counterparts without HIV [91, 92].

The association of age with anal HPV infection varied by region and sexual orientation. One study found anal HPV infection peaked at ages 25–29 years in Italian MSM with HIV. Another study noted there was no significant difference in anal hrHPV infection between French MSM with HIV aged 35–45 and ≥ 55 years. PWH from Colombia with a history of anal intercourse aged > 50 years had lower risk for hrHPV infection than those aged < 30 years [61, 93, 94].

### 3.5 Oral HPV Infection

Few publications reported oral HPV infection prevalence in PWH compared to those without HIV (Supporting Information S1: Table S14 and S15). Oral HPV infection was 1.5–3 times more prevalent in PWH than PWoH (Figure 2F). Most publications reported a lower overall prevalence of oral HPV infections among PWH compared to anal or cervical HPV infection prevalence, ranging from 5.6% to 15.0% (Figure 2A). However, two studies from Mexico reported higher prevalence of oral HPV infection of up to 92.5% of 174 Mexican WWH, and 55.7% of 97 Mexican PWH using a highly sensitive assay [95, 96].

Two studies found that incident oral HPV infections were higher among PWH than PWoH, particularly in MSM [96, 97], however additional studies are needed to draw meaningful conclusions (Supporting Information S1: Table S16).

### 3.6 High‐Risk HPV Genotype Distribution

#### 3.6.1 Cervical hrHPV Infections in WWH

**Figure 3:**
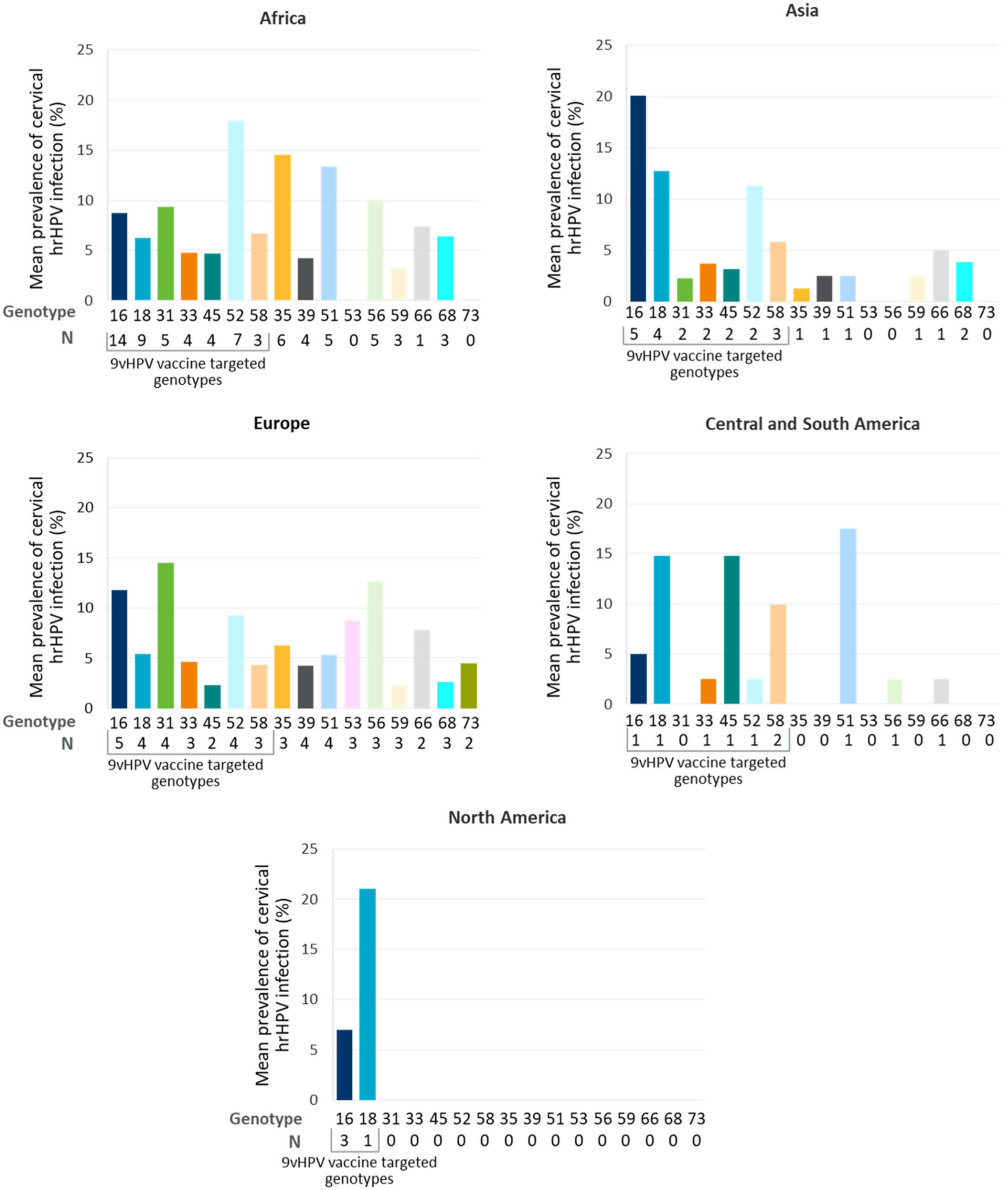
Average cervical hrHPV genotype distribution among WWH across regions. 9v, nonavalent; HPV, human papillomavirus; hrHPV, high‐risk human papillomavirus; N, number of publications; WWH, women with HIV.

Variability exists across studies on the number of HPV genotypes tested and in general, data indicated notable differences in genotype distribution across regions. In Africa, HPV52 was the most prevalent, followed by 35 and 51, while HPV16 in Asia and HPV31 in Europe were the most prevalent genotypes (Figure 3).

#### 3.6.2 Anal hrHPV Infections in PWH

**Figure 4:**
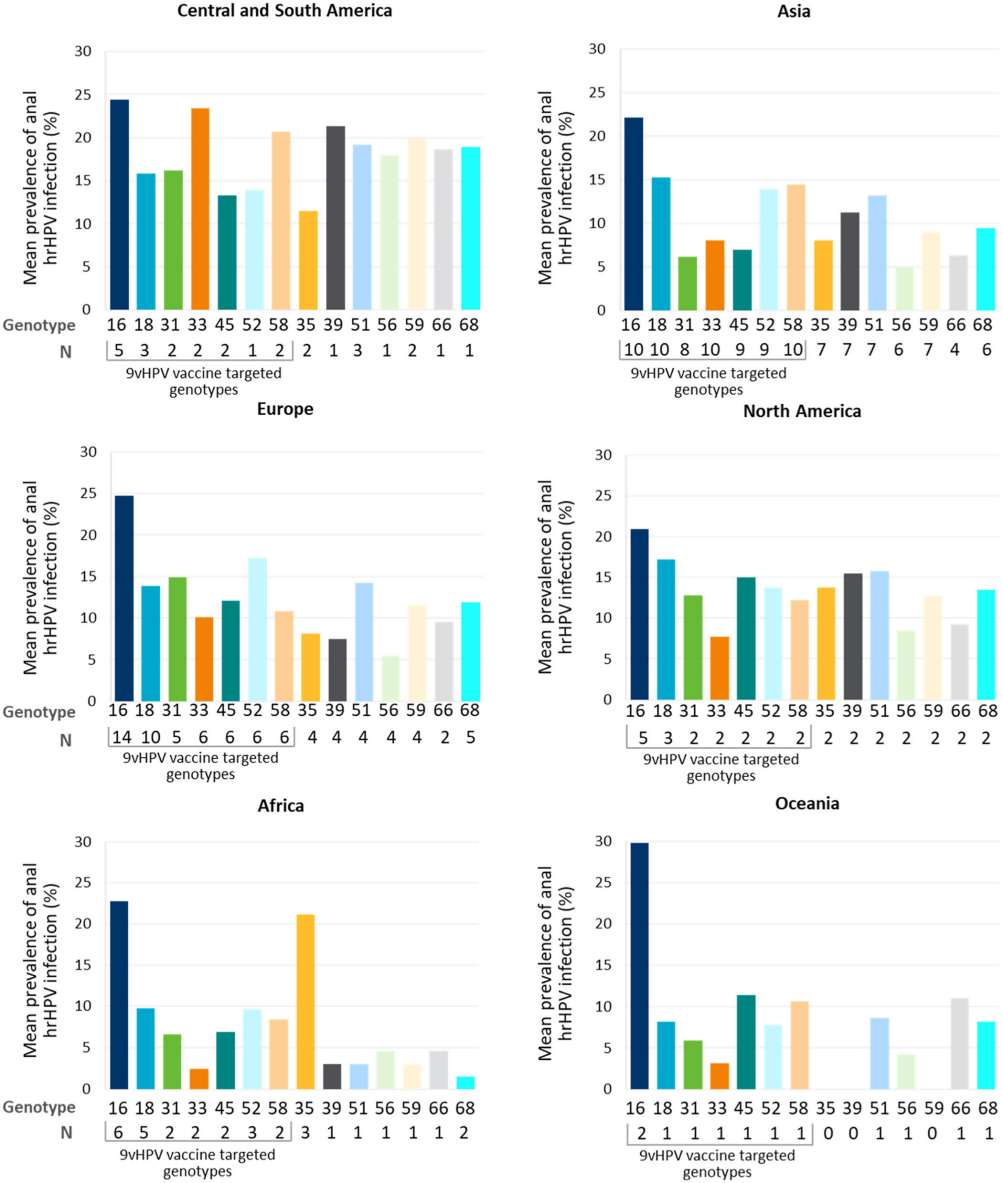
Average anal hrHPV genotype distribution among PWH across regions. 9v, nonavalent; HPV, human papillomavirus; hrHPV, high‐risk human papillomavirus; N, number of publications; PWH, people with HIV.

HPV16 is, on average, the most prevalent genotype globally. While there are differences in geographical distribution, the most notable was the higher prevalence of HPV35 among African PWH, similar to that of HPV16 (Figure 4).

#### 3.6.3 Oral hrHPV Infections in PWH

**Figure 5:**
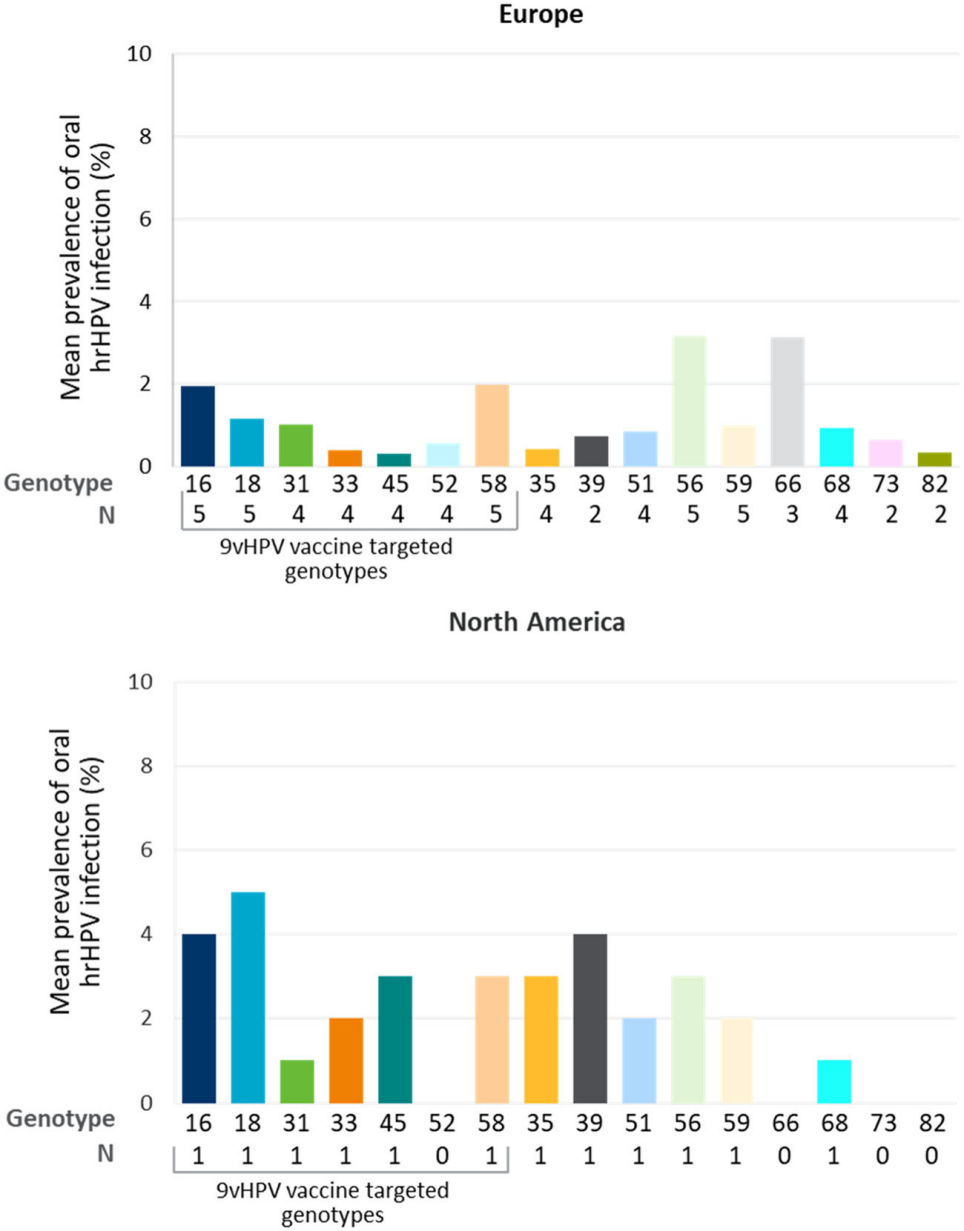
Average oral hrHPV genotype distribution among PWH across regions. 9v, nonavalent; HPV, human papillomavirus; hrHPV, high‐risk human papillomavirus; N, number of publications; PWH, people with HIV.

Although studies on oral HPV infection is limited, available evidence suggested that in Europe HPV56 and HPV66 and in North America HPV18, HPV16, and HPV39 were the most prevalent oral hrHPV types (Figure 5).

### 3.7 HPV Concurrent Infection With Multiple Genotypes

Multiple studies conducted across Europe [15, 16, 18], Africa [20, 21, 87, 98], and Asia [23, 24] suggest that MSM with HIV have a higher prevalence of anal HPV concurrent infection with multiple genotypes compared to MSM without HIV (Supporting Information S1: Table S17). Similar results were reported for cervical infections. One African based study that included four countries found that concurrent infection with multiple hrHPV genotypes were significantly more prevalent among WWH than women without HIV (43.0% vs. 21.6%, respectively; *p* = 0.01) [80]. While comparative data for oral HPV infection with multiple genotypes was limited, a recent US study reported that 9.0% of 245 PWH and 1.0% of 198 PWoH had oral infection with multiple genotypes [32].

### 3.8 Risk Factors

Although not the primary focus of this review, several publications cited low CD4 counts ( < 200 cells/μl) [39, 77, 99, 100], detectable HIV viral load ( > 50 copies/mL) [31, 44, 50, 59, 74, 95, 101, 102], and a shorter duration on antiretroviral therapy (ART) [59, 73, 89, 99, 103, 104] as risk factors for HPV infection and HPV‐related diseases in PWH and, therefore, contribute to increased disease burden of HPV in PWH. However, another study showed that even women receiving effective ART and with high CD4 counts are at increased risk of HPV infection and have higher prevalence of hrHPV infections [51]. Other reported risk factors for HPV infection and related diseases include high‐risk sexual behaviors such as sexual debut < 15 years [92, 100, 101, 105, 106, 107, 108], and having > 5 lifetime sexual partners [32, 52, 95, 100, 101, 104, 108, 109, 110, 111].

### 3.9 Burden of HPV‐Related Diseases

Several studies (*n* = 83) reported on the burden of HPV‐related diseases, including precancers, invasive cancers and anogenital warts (AGW), among which cervical abnormalities (*n *= 48) and cancers (*n* = 17) were the most reported, followed by anal abnormalities (*n* = 10) and cancers (*n* = 8). Supporting Information S1: Figure S6 shows a breakdown of disease burden by anatomical site. The burden of HPV‐related disease at anogenital sites beyond anal and cervical (including penile, vulval and vaginal) were captured in this review, however, were not reported on separately here due to heterogenous and insufficient data, limiting the synthesis of these results. Generally, the prevalence, incidence and risk of anal, cervical, and oral HPV‐related diseases was higher among PWH than PWoH.

### 3.10 HPV‐Related Cervical Precancers

**Figure 6:**
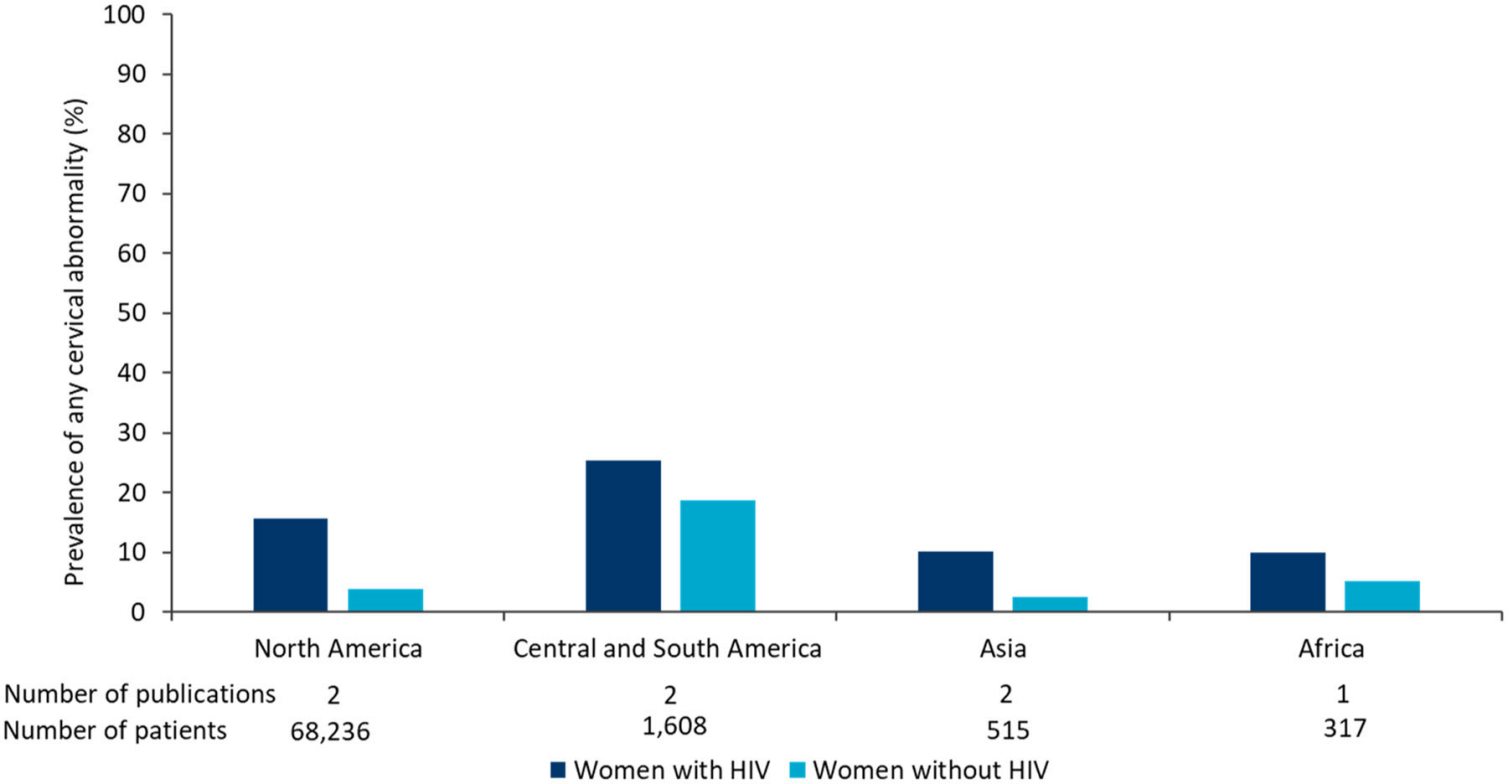
Average weighted prevalence of any cervical abnormality in women with HIV and women without HIV by region [12, 14, 31, 105, 110, 112, 113]. Europe not included due to lack of data. Any cervical abnormality across publications was defined by the inclusion of atypical squamous cells of undetermined significance (ASC‐US), low‐grade squamous intraepithelial lesions (LSIL), high‐grade squamous intraepithelial lesions (HSIL), atypical granular cells of undetermined significance (AG‐US), atypical squamous cells not excluding high grade (ASC‐H), except for one Asian study which did not specify what this encompassed [113]. Abbreviations: HIV: human immunodeficiency virus.

**Figure 7:**
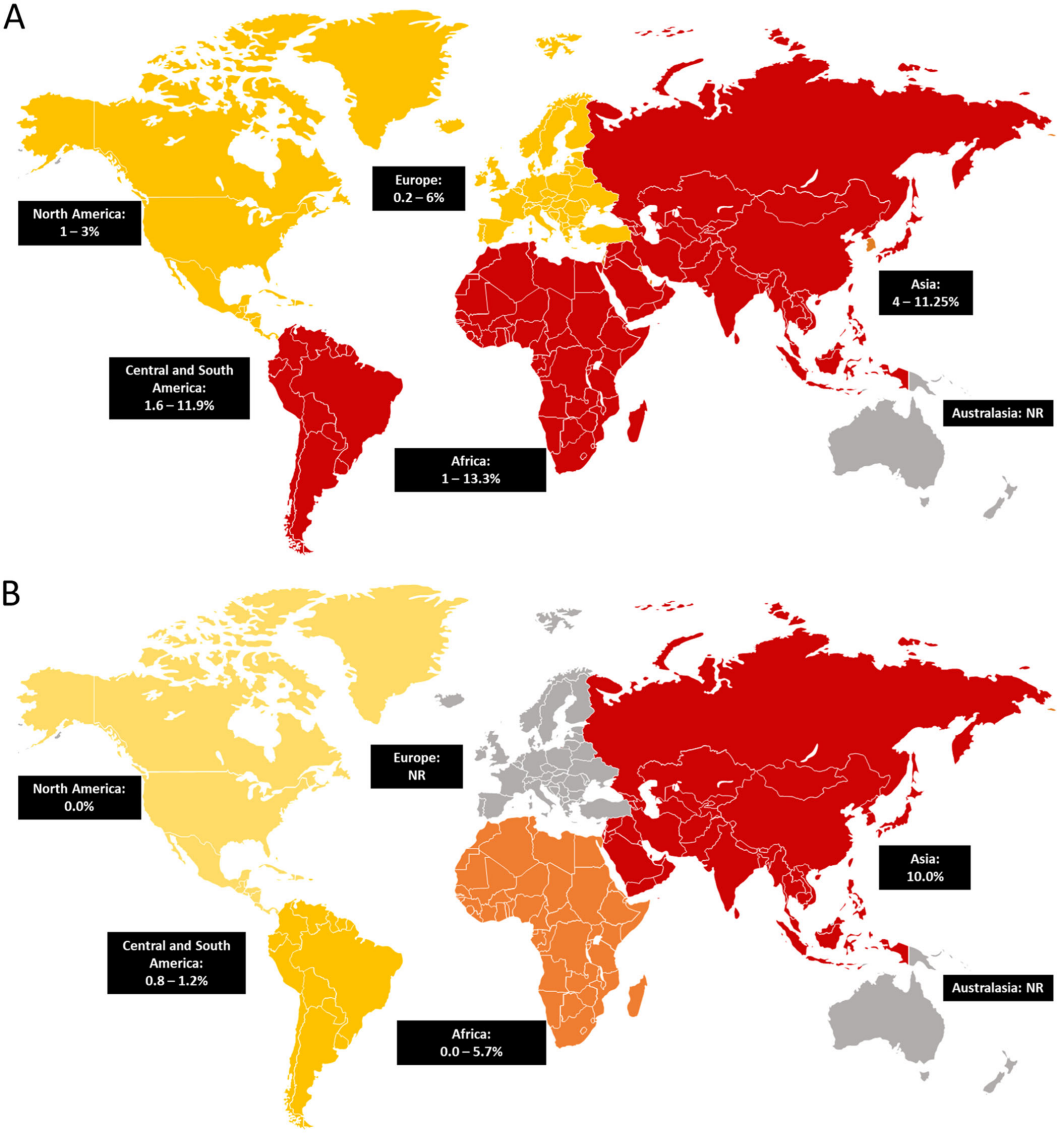
(A) Prevalence of high‐grade cervical abnormalities (HSIL, ASC‐H, CIN2 + ) among women with HIV; North America: US [112, 114]; Central and South America: Colombia [105], Brazil [31, 83, 115]; Europe: Italy [49, 116], Belgium [117], Spain [118]; Africa: South Africa [119], Tanzania [27, 108], Burundi [120], Nigeria [28, 121, 122], Asia: India [13, 46, 104, 123, 124], China [101, 125]; B: Prevalence of high‐grade cervical abnormalities (HSIL, ASC‐H, CIN2 + ) in women without HIV only; North America: US [112], Central and South America: Colombia [105], Brazil [31]; Africa: South Africa [119], Kenya [126], Tanzania [27, 108], Burundi [120], Nigeria [28, 121, 122]; Asia: India [13]. French data from Bouassa et al. (2019) not included due to study population being African migrants [127]. ASC‐H, atypical squamous cells not excluding high grade; CIN, cervical intraepithelial neoplasia; HSIL, high‐grade squamous intraepithelial lesions; NR, not reported.

Cervical precancer lesions was more prevalent in WWH than women without HIV, with the global prevalence of any cervical abnormality being, on average, 1.5–3 times higher in WWH (Supporting Information S1: Table S18 and S19). This prevalence ranged between 5.4% and 31.0% for WWH and 1.8%–20.4% for women without HIV, with higher prevalence reported in Central and South America than North America and Asia (Figure 6). Similar results were seen for high‐grade abnormalities only (Figure 7).

While age‐stratified data were scarce, of the 34 publications reporting prevalence of cervical precancer abnormalities, most cases were among WWH within the 30–45‐year age range, suggesting that cervical abnormalities may be more prevalent in younger age groups.

### 3.11 HPV‐Related Cervical Cancer

Incident cervical cancer was 1.5–6.6 times greater among WWH in comparison to their counterparts without HIV, dependent on the histological subtype of cancer investigated (Supporting Information S1: Tables S20 and S21) [128, 129].

The highest prevalence of cervical cancer were reported in those with the mean ages between 30 and 55 years [13, 27, 130]. While age‐stratified cervical cancer prevalence and incidence data in WWH were limited, three studies reported higher incidence and prevalence rates between 35 and 54 years of age than those < 35 years or > 54 years. Comparative data suggests that cervical cancer prevalence peaks among younger WWH (35–44 years) in comparison to women without HIV (peak at ≥ 55 years) [129].

### 3.12 HPV‐Related Anal Abnormalities

Comparative studies (*n* = 4) reported prevalence of HPV‐related anal abnormalities 1.7–2.1 times higher among PWH compared to PWoH, with variation due to the study subpopulation and type of anal abnormality investigated (Supporting Information S1: Figure S7 and Table S22). Sex differences influence the burden of anal abnormalities. MWH had higher prevalence and incidence of anal abnormalities than WWH in North America and Asia, although additional data may be required to confirm this finding [110, 131, 132]. People with HPV‐mediated anal lesions had faster rates of progression to carcinoma in situ (CIS) and to invasive carcinoma in people with versus without HIV, which was further enhanced by low CD4 counts [133].

### 3.13 HPV‐Related Anal Cancer

Two publications reported a higher incidence and risk of anal cancer among PWH than PWoH (Table S23). A study in the US showed age‐adjusted incidence rate of anal cancer among American women with HIV and without HIV was 12.6 (95% CI: 0–30.9) and 8.4 (95% CI: 0.8–15.9) per 100,000 person‐years, respectively [128]. Similarly, PWH were at higher risk of HPV‐related anal disease progression than PWoH (high‐grade squamous intraepithelial lesions [HSIL] to CIS or further; HR: 1.43; *p* = 0.02) and CIS to squamous cell carcinoma [SCC; HR: 2.08; *p* = 0.03]) [134]. Additional studies from the US (*n* = 3) reported that MWH had a higher prevalence and incidence of HPV‐related anal cancer than WWH [131, 135, 136]. There were no data reporting on the incidence of anal cancer among MSM with HIV.

### 3.14 HPV‐Related Oral and Head and Neck Cancer

There were limited comparative data reporting on oral and head and neck cancers (Table S24). A French study reported that incident head and neck cancer was 2.6 times higher among PWH than PWoH (79.1 vs. 30.7 per 100,000 person‐years), and noted an association with age [137]. PWH aged 50–59 years had the highest incidence rate (122.6 per 100,000 person‐years) compared to those aged 18–39 years, 40–49 years, and ≥ 60 years (0, 66.3, and 110.9 per 100,000 person‐years, respectively) [137].

The epidemiology of oral cancer differed with ethnicity; in the US, non‐Hispanic black individuals had a higher burden of oral cancer than Hispanic and non‐Hispanic white individuals [135, 136].

### 3.15 Genotype Distribution in HPV‐Related Diseases

HPV genotype distribution data among PWH with HPV‐related disease was identified as an important research gap within the current literature. One study highlighted that, in anal condyloma tissues, HPV16, HPV52 and HPV58 high‐risk genotypes occurred in higher frequencies among MSM with HIV versus without HIV [138].

Similar to HPV infections, available data indicated that PWH with HPV‐related diseases had an overall higher number of HPV genotypes [105]. American PWH with HPV‐mediated anal lesions, including anal intraepithelial neoplasia (AIN) 1, 2, 3, low‐grade squamous intraepithelial lesions (LSIL), HSIL or invasive SCC, had a higher overall genotype diversity than PWoH (7 vs. 4 median number of genotypes, respectively) [133]. This higher HPV strain diversity was associated with faster progression from AIN1/LSIL to AIN2‐3/HSIL [133]. Additionally, PWH with HPV‐mediated anal lesions had significantly higher infection rates with HPV strains 35, 39, 52, 53, 56, 58, 59, 66, and 68. HPV39, 58, 66, and 67 were associated with faster progression in both PWH and PWoH.

Kremer et al. (2018) noted that among 123 and 22 South African WWH with cervical intraepithelial neoplasia (CIN) 3 and invasive cervical cancer (ICC), respectively, HPV16 was the most prevalent genotype (CIN3: 31.7%; ICC: 45.5%), followed by HPV18 (CIN3: 13.6%; ICC: 13.6%), and HPV45, (CIN3: 13.6%; ICC: 13.6%) [139]. Tawe et al. (2020) also reported similar findings with the most prevalent genotype being HPV16 (47.7%) among 88 Botswanan WWH with ICC [140]. Teixeira et al. (2018) reported that among 21 and 10 Brazilian WWH with cervical LSIL and HSIL, respectively, 52.3% and 60.0% were infected with genotypes HPV56/59/66, respectively, while 23.8% and 50.0% were infected with HPV16, respectively [83]. A study from China reported that hrHPV infections increased with increasing severity of histological abnormalities from 17.6% in women with normal findings to 88.9% in those with LSIL and 92.0% for HSIL. HPV16, HPV52, and HPV58 were the most common hrHPV types in women with HSIL [101]. Another study from Belgium reported that the most common hrHPV types in abnormal cytology were HPV31, HPV52, and HPV66 [117].

## 4 Discussion

This study showed that PWH have a higher prevalence and incidence of HPV infection than PWoH across all anatomical sites. Prevalent cervical infections were up to six times higher among WWH than women without HIV depending on the region, while prevalent anal and oral HPV infections were about twice as high among PWH than PWoH. These results are consistent with previous systematic reviews and studies conducted across broader and earlier time periods. Previous systematic review that synthesized studies published between 1986 and 2017 reported that the prevalence of anal HPV infection was 1.3–1.4 times higher among PWH, while another systematic review conducted in 2015 reported that oral HPV infection prevalence 1.5 times higher among HIV‐positive MSM compared to HIV‐negative MSM [141, 142]. However, contrary to the findings of this review, a relatively older study conducted in 2008 reported comparable prevalences of cervical HPV infection between South African WWH and women without HIV [143].

Moreover, HPV‐related diseases were consistently higher among PWH than PWoH. Compared to women without HIV, cervical precancers were 1.5–3.0 times more prevalent in WWH, and incident cervical cancers were up to six times higher in WWH, perhaps suggesting a faster progression of precancer to invasive cervical cancer in PWH. Furthermore, the prevalence of anal abnormalities was about two‐fold higher among PWH than PWoH, and the incidence of head and neck cancer was also over two times higher among PWH than PWoH.

The prevalences and incidences of HPV infection and related diseases varied depending on subpopulations and anatomical sites. MSM with HIV had a higher prevalence of anal HPV infection compared to WWH or MWH who have sex with women. Average prevalence differences between MSM with and without HIV were smaller than differences in other subgroups (e.g., WWH vs. women without HIV), suggesting that sexual behavior has a greater influence on the prevalence of anal HPV infection than HIV status in this subpopulation. This aligns with findings from a recent meta‐analysis, which reported that the most important population‐level determinant of anal HPV infection, aside from HIV status, was sexual orientation [144].

As with HPV infections, the burden of HPV‐related anal diseases, including invasive cancer and both high‐ and low‐grade anal abnormalities, is higher among men with HIV than women with HIV, 2‐to‐7 fold higher anal precancers and 2‐to‐25 fold higher anal cancer, potentially due to the presence of MSM within male study populations [141]. The risk of progression from precancer to anal carcinoma in situ and to invasive anal carcinoma was reported to be about two‐fold higher in PWH versus PWoH. Receptive anal sex is a well‐documented risk factor for anal HPV infection in the literature and absence of formal guidelines recommending anogenital screening for men across most countries exacerbates the issue, leading to recurrent anal HPV infections and the development of HPV‐related anal diseases [135, 145].

The burden of HPV infection and HPV‐related disease among PWH varied by geographical region, however comparative studies are limited. For example, a higher prevalence of cervical HPV infection and high‐grade cervical abnormalities has been observed in Africa versus North America and Europe. Geographical differences in cervical HPV infection and related disease may be attributed to variations in availability of HPV vaccination, low HPV vaccine uptake, lack of cervical screening, and decreased access to healthcare facilities, leading to high rates of persistent HPV infections in certain countries [146]. Additional research into HPV infection and related diseases in PWH across geographical regions is required to categorize the burden.

The burden of HPV infection and HPV‐related disease also varies with age. Some studies reported a higher prevalence of cervical HPV infection among women aged 25–39 years than older women, while others found prevalent cervical infection with hrHPV increased among women aged ≥ 36 years and peaked in women aged ≥ 50 years. The reasons for variations with age are unclear due to limited data and heterogeneity of populations within the studies; however, it was suggested that the prevalence of hrHPV peaking among ≥ 50 years was attributed to higher rates of HPV persistence due to impaired clearance and reactivation of latent infection during the perimenopausal period rather than new HPV infection acquisition [83, 147]. Additionally, changes in sexual behavior during middle age may also play a role in prevalence increases among older women [83]. These findings suggests the importance of expanding vaccination to the older ages, beyond the current recommendations.

Alarmingly, incident cervical cancer rates are highest among younger WWH, specifically those aged 35–54 years, compared to those aged > 55 years. Comparative data also suggested that cervical cancer prevalence peaked among younger WWH (35–44 years) in contrast to women without HIV, for whom prevalence peaks at age ≥ 55 years. Similarly, high prevalence of precancer cervical lesions in WWH was among those aged within the 30–45‐years range, suggesting that cervical precancers and cancer may be more prevalent at younger age in WWH than in women without HIV, and perhaps indicating faster progression to cancer in WWH. A previous systematic review noted that incident cervical cancer was highest among women without HIV of age > 70 years in most geographical regions [148].

Analysis of hrHPV genotypes among PWH revealed a variable distribution across most geographical regions. In cervical HPV infections, HPV16 and HPV31 are the most prevalent in Asia, while HPV52 and HPV35 are the most prevalent in Africa. This is consistent with two published systematic reviews that identified HPV35 one of the most prevalent genotypes in cervical HPV infections among African women [149, 150]. Among PWH with anal HPV infection, HPV16 was the most prevalent genotype globally, whereas HPV35 had an elevated prevalence in Africa. Finally, there were no clear distribution trends among PWH with oral infection due to a paucity of data. Whilst our findings suggest genotype prevalence can vary, HPV35 remains a highly prevalent genotype in African populations, and it is not currently targeted by vaccination.

Genotype distribution data for PWH with HPV‐related disease were limited, indicating a significant gap in the evidence. It has been noted that high‐risk, vaccine‐preventable genotypes (HPV16, 18, and 45) and infection with multiple genotypes (HPV56/59/66) were the most prevalent among African and Mexican WWH with cervical disease [83, 117, 139]. Interestingly, among Asian MSM with anal condylomas, hrHPV genotypes were found at significantly higher frequencies in MSM with HIV than counterparts without HIV, despite the fact that anal condylomas are typically caused by low‐risk HPV types [138].

PWH with HPV‐related anal diseases were concurrently infected with a wider diversity of genotypes compared to PWoH. This is may be due to a multitude of reasons, such as immunosuppression resulting from low CD4 cell counts, increased high‐risk sexual behavior, or an immune response to anal lesions that may in turn mediate infection with rare HPV types [133]. Further research into the associated genotypes with HPV‐related diseases can provide key evidence to support vaccination programs aimed at alleviating the disease burden.

Several publications have reported that low CD4 counts, detectable HIV viral load, and a shorter duration on ART are risk factors for HPV infection and HPV‐related diseases in PWH. However, some research has also found that WWH who are receiving effective ART and have high CD4 counts still face an increased risk of HPV, indicating that other factors are at play in addition to immune depression [51].

The findings of this review indicate substantial heterogeneity in reporting across anatomical sites and patient subpopulations. Therefore, careful interpretation of results is required. Notably, heterogeneity in the reporting of stratified results, particularly among subpopulations such as MSM and MSW, has limited our conclusions on the impact that HIV may have on these groups. Heterogeneity may stem from differences in data collection methods‐ many publications captured genotype distribution data pertaining only to genotypes in the 9‐valent vaccine or fewer‐ as well as diversity in methodology (e.g., diagnosis via cytological/histological tests or visual inspections), sociodemographic characteristics and risk exposures within study populations. Furthermore, there is a lack of consensus on the definitions within the literature, including on the risk status and oncogenicity of various HPV genotypes. This has resulted to variations in prevalence and incidence figures across publications, with the same genotype being classified as high risk in one study and low risk in another. Publications investigating HPV‐related diseases often lacks standardized criteria for defining anogenital abnormalities, such as atypical squamous cells of undetermined significance. Moreover, some studies did not specify whether oral disease burden was HPV‐related, resulting in limited data for this anatomical site. Geographical differences should be interpreted with caution, as they may include populations who have recently migrated, affecting the burden of HPV infection and disease across countries [127].

## 5 Conclusion

This systematic review confirms that PWH have a significantly higher burden of HPV infections as well as HPV‐related precancers and invasive cancers than PWoH. The burden varies with age, gender, sexual orientation, and geographic region. Comparisons with studies from earlier time periods suggest that the disparity in the burden of cervical HPV infection rates has increased in recent years among WWH in comparison to women without HIV. This trend may be attributed to the success of HPV vaccination programs that have primarily focused on vaccinating younger women, with less emphasis on increasing vaccination access in those living with HIV. To address this disparity, HPV prevention strategies, such as vaccination should be bolstered among PWH, especially in regions with high HIV prevalence. Populations that warrant targeted HPV prevention strategies with vaccination include WWH due to their increased cervical hrHPV infection prevalence and cervical cancer incidence, as well as MSM with HIV due to an elevated risk of anal HPV infection, compared to other subgroups [147]. Among WWH with cervical HPV infections, vaccine preventable genotypes were found to be the most prevalent. The prevalence of these genotypes suggests that the current vaccination and screening strategy may be insufficient for WWH. Future prevention measures should also target prevalent oncogenic genotypes that are not currently covered by vaccines, such as HPV35, which has been frequently reported among PWH in Africa. Furthermore, the high prevalence of concurrent infection with multiple HPV genotypes underscores the need to roll out a higher valent HPV vaccine to gain maximum protection. Lastly, implementation of HPV vaccination by integrating it into routine HIV care and clinics providing HIV prevention for high‐risk groups may provide a valuable opportunity to increase HPV vaccine uptake while also promoting efficiency in delivery.

## Author Contributions

Alisa Chowdhary, Jia Pan, and Ana Costa developed the study protocol, performed the literature search, drafted the study results, developed the first draft of the manuscript, and critically reviewed the manuscript drafts. Xuedan You and Ya‐Ting Chen were involved in the conception of the study, and review of the protocol development. Bekana K. Tadese, Tidiane Ndao, Xuedan You, Ya‐Ting Chen, and Joseph E. Tota were involved in the review of the study results, interpretations, and critical review of versions of the manuscript drafts. Nelly Mugo was involved in the review of the study results and critical review of the manuscript drafts. All authors reviewed the results from the systematic literature review, reviewed manuscript drafts, and approved the final version for submission.

## Conflicts of Interest

B.K.T., X.Y., T.N., J.E.T., and Y.C. are employees of Merck & Co. Inc. Rahway, NJ, USA. Alisa Chowdhary, Jia Pan, and Ana Costa are employees of Adelphi Values PROVE and were funded by Merck Sharp & Dohme LLC, a subsidiary of Merck & Co. Inc. Rahway, NJ, USA to conduct this research. Nelly Mugo is employee of Kenya Medical Research Institute, Nairobi City, Kenya.

## Supporting information

Supporting information.

## Data Availability

Data for this manuscript was from published studies, all of which have been cited appropriately in text and tables.
