# Supplementary material for: The Burden of HPV Infections and HPV‐Related Diseases Among People With HIV: A Systematic Literature Review
Source: J Med Virol. 2025 Apr 2;97(4):e70274. doi: 10.1002/jmv.70274 (PMC11963496; doi:10.1002/jmv.70274)
Supplement: Supplementary file 1 — Supporting information. [file JMV-97-e70274-s001.docx]

# **Supplementary material**

## **Supplementary Figures**

### Supplementary Figure 1. JBI critical appraisal tool checklist for cross sectional studies

### Supplementary Figure 2. JBI critical appraisal tool checklist for studies reporting prevalence data

### Supplementary Figure 3. JBI critical appraisal tool checklist for case-control studies

### Supplementary Figure 4. Quality assessment results using the JBI critical appraisal tool


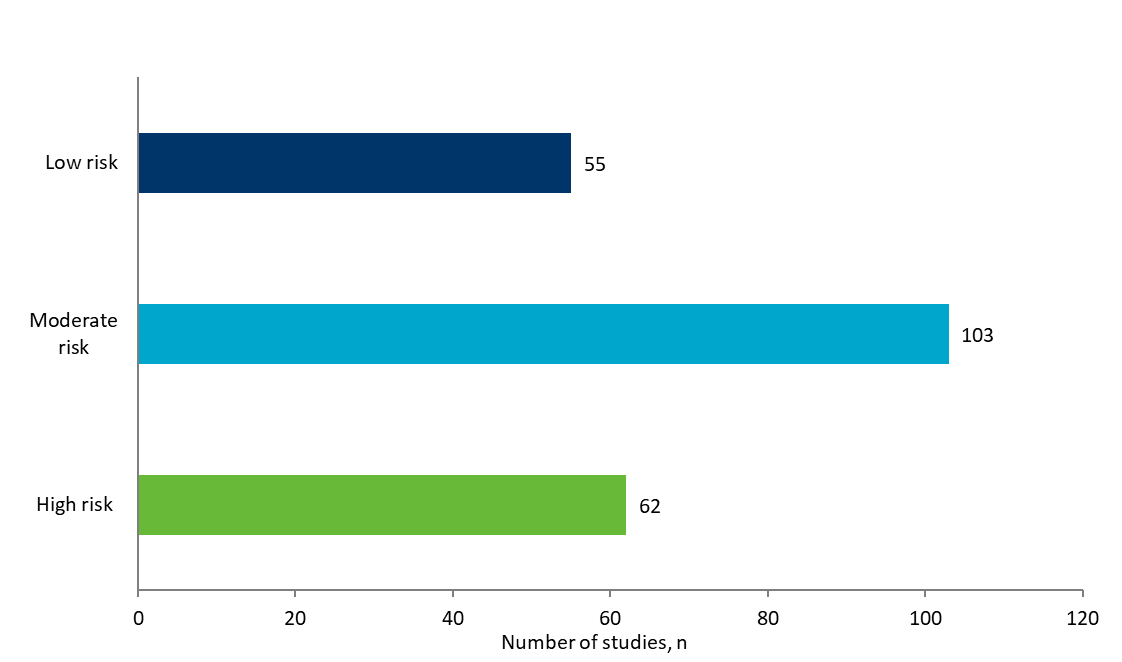


### Supplementary Figure 5. Breakdown of studies identified in the database search reporting prevalence of HPV infection (N=184), by anatomical site*

**
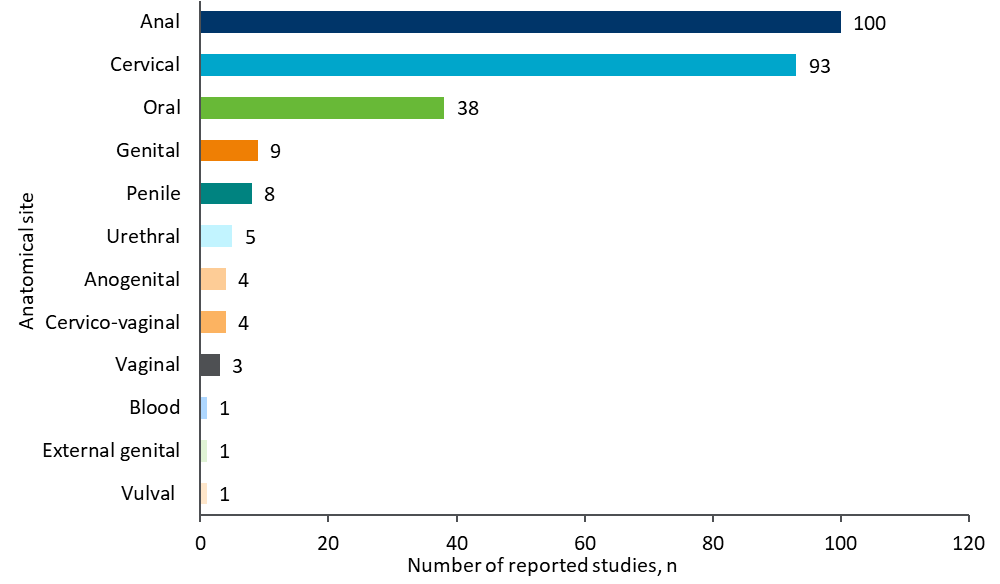
**

*Number does not add up to 184 as anatomical sites include concurrent infections and studies reporting multisite infections.

### Supplementary Figure 6. Breakdown of studies identified in the database search reporting on the burden of the type of HPV-related disease and anatomical site (N=83)*

**
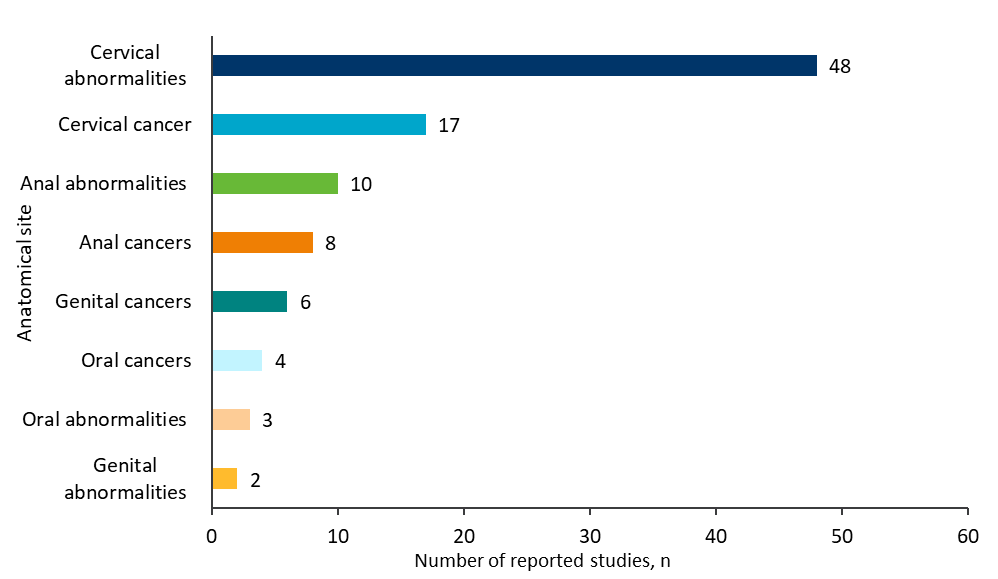
**

*Number does not add up to 83 as individual studies may have reported on more than one anatomical site

### Supplementary Figure 7. Prevalence of any anal abnormality in PWH and PWoH


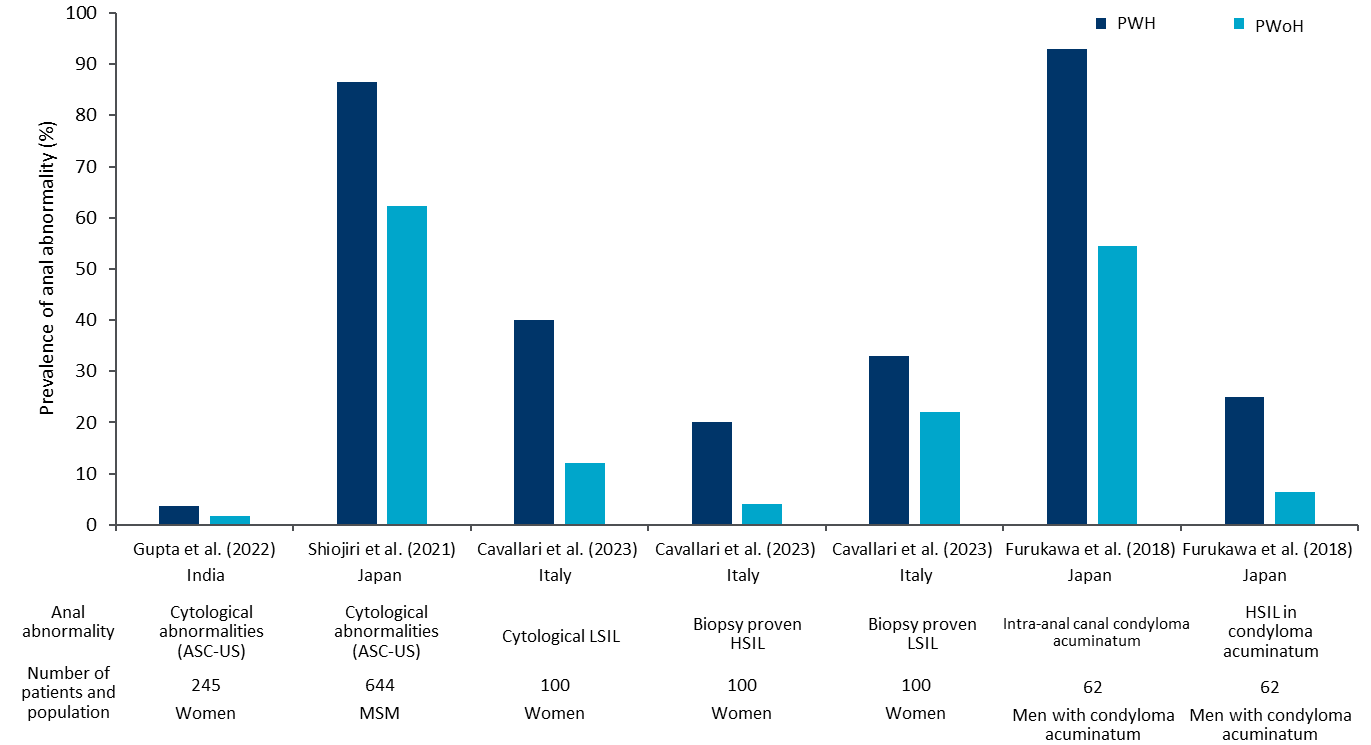


Abbreviations: ASC-US: Atypical squamous cells of undetermined significance; HIV: human immunodeficiency virus; HPV: human papillomavirus; HSIL: high-grade squamous intraepithelial lesion; LSIL: low-grade squamous intraepithelial lesion; MSM: men who have sex with men, PWH: people with HIV; PWoH: people without HIV.

## **Supplementary Tables**

### Supplementary Table 1. Search terms used for gray literature searches

| **Gray literature** | **Year** | **Search term** | **Number of hits** |
| --- | --- | --- | --- |
| **Conferences** | | | |
| **International papillomavirus conference** | 2023 | HIV and HPV | 2 |
|  |  | HIV with HPV | 1 |
| **EUROGIN** | 2023 | HIV and HPV | 0 |
|  |  | HIV with HPV | 0 |
|  |  | HIV | 6 |
|  | 2022 | HIV | 11 |
|  | 2021 | HIV | 0 |
| **International AIDS society conference** | 2023 | NA | 23 |
|  | 2022 | NA | 8 |
|  | 2021 | NA | 7 |
| **Epidemiological reports** | | | |
| **WHO** | 2023 | HPV with HIV | 0 |
|  |  | HIV and HPV | 0 |
|  |  | HIV HPV | 0 |
| **CDC** | 2023 | HPV with HIV | 0 |
|  |  | HIV and HPV | 0 |
|  |  | HIV HPV | 0 |
| **ECDC** | 2023 | HIV and HPV | 4 |

Abbreviations: AIDS: Acquired immunodeficiency syndrome; CDC: Centers for Disease Control and Prevention; ECDC: The European Centre for Disease Prevention and Control; HIV: human immunodeficiency virus; HPV: human papillomavirus; WHO: World Health Organization.

### Supplementary Table 2. PICOTS eligibility criteria

| Topic | Inclusion criteria | Exclusion criteria |
| --- | --- | --- |
| Population(s) | - Adults aged ≥18 years of age with HIV with HPV infections or HPV-related diseases - HPV infections of anogenital or oral regions - HPV-related diseases will include: - AGWs - Precancers or cancers of anogenital regions (including cervix, vulva, vagina, penis, or anus) - Precancers or cancers of head and neck (including oral cavity, larynx, or oropharyngeal regions) - RRP | - Children aged <18 years of age - Populations of mixed ages where results related to adults (>18 years) are not disaggregated |
| Intervention | - No interventional data expected | - N/A |
| Comparators | - No comparator data expected | - N/A |
| Outcomes | - Risk factors including: - Sexual behavior exposure (e.g., number of lifetime sexual partners, age at first sex, sex workers) - Behavioral risk (e.g., smoking or use of contraceptives) - Sociodemographic characteristics (e.g., age, education level, socioeconomic status, ethnicity) - HIV disease control (e.g., low CD4 count, history of AIDS stage) - Co-infection with other STIs - Other medical conditions - Access to HPV prevention - Epidemiology outcomes including: - Prevalence - Incidence - Mortality - Clinical outcomes including: - Disease burden - Morbidity - Hospitalization rates - Humanistic outcomes including: - QoL (including but not limited to physical and mental health perceptions, functional status, social support, and socioeconomic status) - Economic outcomes including: - Direct and indirect medical costs - Healthcare resource utilization | - Publications not reporting outcomes of interest - Publications where results related to PWH are not disaggregated from PWoH |
| Time horizon | - January 1st, 2018–June 5th, 2023 | - Publications published pre-2018 |
| Study design | - Observational studies (retrospective and prospective) - Longitudinal studies - Cross-sectional studies - Registry studies - Cohort studies - Case-control studies - Cost-effectiveness studies (input data only) | - RCTs - Case studies or case reports - Editorials - Reviews (including SLRs) |
| Other | - Human studies only - English language studies only - Worldwide (all geographies) | - Animal studies - Non-English language studies - Conference abstracts that report the same data as a subsequent full-text publication will be marked as duplicates and excluded |

Abbreviations: AIDS: Acquired immunodeficiency syndrome; AGW: anogenital wart; HIV: human immunodeficiency virus; HPV: human papillomavirus; N/A: not applicable; PWH: people with HIV; PWoH: people without HIV; QoL: quality of life; RCT: randomized controlled trial; RRP: recurrent respiratory papillomatosis; SLR: systematic literature review; STI: sexually transmitted infection.

### Supplementary Table 3. Search strategy for EMBASE (12th June 2023)

| **#** | **Category** | **Query** | **Results** |
| --- | --- | --- | --- |
| 1 | Population | papillomavirus infection/ | 22,132 |
| 2 |  | (human papilloma virus or human papillomavirus or hpv).tw. | 82,476 |
| 3 |  | or/1-2 | 85,832 |
| 4 | Sub-population | exp HIV/ or exp acquired immune deficiency syndrome/ | 332,674 |
| 5 |  | exp HIV Infections/ | 412,518 |
| 6 |  | (((HIV or human immunodeficien* or human immun# deficien*) adj3 (patient? or individual? or people)) or AIDS).tw. | 290,742 |
| 7 |  | or/4-6 | 613,788 |
| 8 |  | 3 and 7 | 6,505 |
| 9 | Epidemiology | epidemiology/ | 156,954 |
| 10 |  | Incidence/ | 571,244 |
| 11 |  | prevalence/ | 940,652 |
| 12 |  | mortality/ | 865,385 |
| 13 |  | morbidity/ or *disease burden/ | 411,998 |
| 14 |  | (epidemiolog* or incidence* or prevalen* or mortalit* or morbid* or burden).tw. | 4,447,629 |
| 15 |  | or/9-14 | 5,004,375 |
| 16 | Humanistic burden | quality of life/ | 617,085 |
| 17 |  | patient-reported outcome/ | 53,421 |
| 18 |  | patient preference/ | 26,310 |
| 19 |  | psychological well being/ | 30,253 |
| 20 |  | caregiver burden/ | 10,593 |
| 21 |  | (qol or hrqol or quality of life).tw. | 596,043 |
| 22 |  | ((patient* or caregiver* or carer* or parent* or population? or people or person* or individual*) adj3 (satisf* or perspective* or view* or prefer* or attitude* or outcome* or burden or stress* or well being or perce* or pressur* or anxi* or depress*)).tw. | 1,390,154 |
| 23 |  | or/16-22 | 2,058,047 |
| 24 | Costs and resource use | cost of illness/ | 21,182 |
| 25 |  | disease burden/ | 44,649 |
| 26 |  | Sick Leave/ | 7,575 |
| 27 |  | healthcare financing/ | 13,862 |
| 28 |  | productivity/ | 47,490 |
| 29 |  | caregiver burden/ | 10,593 |
| 30 |  | length of stay/ | 265,348 |
| 31 |  | health care utilization/ | 92,621 |
| 32 |  | ((economic or societ* or socioeconomic or socio economic or illness or disease or patient* or caregiver* or carer* or productiv* or employ*) adj3 (burden or impact or consequence?)).tw. | 273,621 |
| 33 |  | (productivity or cost* or expenditure* or expens* or financ* or visit* or inpatient* or outpatient* or hospitali* or financ* or (length adj5 stay)).tw. | 2,656,802 |
| 34 |  | (resource* adj2 ("use" or usage or utili* or allocat*)).tw. | 79,247 |
| 35 |  | (leave adj2 (medical or sick or disability)).tw. | 8,211 |
| 36 |  | or/24-35 | 3,040,096 |
| 37 | Total before any exclusion limits | 8 and 15 | 3,485 |
| 38 |  | 8 and 23 | 295 |
| 39 |  | 8 and 36 | 1,001 |
| 40 |  | or/37-39 | 3,970 |
| 41 | Exclusion terms | exp case study/ or exp case report/ or exp letter/ or exp editorial/ or exp preliminary communication/ or exp note/ | 4,978,232 |
| 42 |  | (exp animal/ or exp invertebrate/ or nonhuman/ or animal experiment/ or animal tissue/ or animal model/ or exp plant/ or exp fungus/) not (exp human/ or human tissue/) | 6,388,778 |
| 43 |  | 40 not (41 or 42) | 3,734 |
| 44 |  | exp conference paper/ or conference abstract/ or (conference adj (abstract or paper or review or proceeding)).pt. | 5,581,260 |
| 45 |  | limit 44 to yr="2000 - 2019" | 4,218,213 |
| 46 |  | 43 not 45 | 2,803 |
| 47 | Total after language limit | limit 43 to english language | 3,596 |
| 48 | Total after time limit | limit 47 to yr="2012 -Current" | 2,626 |
| 49 |  | exp conference paper/ or conference abstract/ or (conference adj (abstract or paper or review or proceeding)).pt. | 5,581,260 |
| 50 |  | limit 49 to yr="2012 - 2019" | 3,084,142 |
| 51 |  | 48 not 50 | 1,877 |
| 52 | Total after duplicates removed | remove duplicates from 51 | 1,792 |

### Supplementary Table 4. Search strategy for Medline (12th June 2023)

| **#** | **Category** | **Query** | **Results** |
| --- | --- | --- | --- |
| 1 | Population | papillomavirus infection/ | 33,096 |
| 2 |  | (human papilloma virus or human papillomavirus or hpv).tw. | 60,509 |
| 3 |  | or/1-2 | 64,052 |
| 4 | Sub-population | exp HIV/ or exp Acquired Immunodeficiency Syndrome/ | 174,752 |
| 5 |  | exp HIV Infections/ | 315,072 |
| 6 |  | (((HIV or human immunodeficien* or human immun# deficien*) adj3 (patient? or individual? or people)) or (Acquired Immunodeficiency Syndrome or AIDS)).tw. | 244,722 |
| 7 |  | or/4-6 | 427,578 |
| 8 |  | 3 and 7 | 3,405 |
| 9 | Epidemiology | epidemiology/ | 12,576 |
| 10 |  | Incidence/ | 300,439 |
| 11 |  | prevalence/ | 342,574 |
| 12 |  | mortality/ | 49,397 |
| 13 |  | morbidity/ or *disease burden/ | 48,009 |
| 14 |  | (epidemiolog* or incidence* or prevalen* or mortalit* or morbid* or burden).tw. | 3,160,025 |
| 15 |  | or/9-14 | 3,326,325 |
| 16 | Humanistic burden | quality of life/ | 266,870 |
| 17 |  | patient-reported outcome/ | 13,432 |
| 18 |  | patient preference/ | 10,649 |
| 19 |  | psychological well being/ | 173 |
| 20 |  | caregiver burden/ | 589 |
| 21 |  | (qol or hrqol or quality of life).tw. | 363,118 |
| 22 |  | ((patient* or caregiver* or carer* or parent* or population? or people or person* or individual*) adj3 (satisf* or perspective* or view* or prefer* or attitude* or outcome* or burden or stress* or well being or perce* or pressur* or anxi* or depress*)).tw. | 902,357 |
| 23 |  | or/16-22 | 1,259,311 |
| 24 | Costs and resource use | cost of illness/ | 31,508 |
| 25 |  | disease burden/ | 31,508 |
| 26 |  | Sick Leave/ | 6,741 |
| 27 |  | healthcare financing/ | 1,248 |
| 28 |  | productivity/ | 15,557 |
| 29 |  | caregiver burden/ | 589 |
| 30 |  | length of stay/ | 102,260 |
| 31 |  | health care utilization/ | 54,738 |
| 32 |  | ((economic or societ* or socioeconomic or socio economic or illness or disease or patient* or caregiver* or carer* or productiv* or employ*) adj3 (burden or impact or consequence?)).tw. | 168,702 |
| 33 |  | (productivity or cost* or expenditure* or expens* or financ* or visit* or inpatient* or outpatient* or hospitali* or financ* or (length adj5 stay)).tw. | 1,853,092 |
| 34 |  | (resource* adj2 ("use" or usage or utili* or allocat*)).tw. | 52,526 |
| 35 |  | (leave adj2 (medical or sick or disability)).tw. | 6,452 |
| 36 |  | or/24-35 | 2,094,023 |
| 37 | Total before any exclusion limits | 8 and 15 | 1,868 |
| 38 |  | 8 and 23 | 120 |
| 39 |  | 8 and 36 | 470 |
| 40 |  | or/37-39 | 2,056 |
| 41 | Exclusion terms | exp case study/ or exp case report/ or exp letter/ or exp editorial/ or exp preliminary communication/ or exp note/ | 3,970,203 |
| 42 |  | (exp animal/ or exp invertebrate/ or nonhuman/ or animal experiment/ or animal tissue/ or animal model/ or exp plant/ or exp fungus/) not (exp human/ or human tissue/) | 5,555,659 |
| 43 |  | 40 not (41 or 42) | 1,993 |
| 44 | Total after language limit | limit 43 to english language | 1,906 |
| 45 | Total after time limit | limit 44 to yr="2012 -Current" | 1,183 |
| 46 | Total after duplicates removed | remove duplicates from 45 | 1,183 |

### Supplementary Table 5. Search strategy for Evidence-Based Medicine Reviews (EBMR: ACP Journal Club, Database of Abstracts of Reviews of Effects, Cochrane Central Register of Controlled Trials, Health Technology Assessment, Cochrane Database of Systematic Reviews, National Health Service Economic Evaluation, Cochrane Methodology register databases; 12th June 2023)

| **#** | **Category** | **Query** | **Results** |
| --- | --- | --- | --- |
| 1 | Population | papillomavirus infection/ | 212 |
| 2 |  | (human papilloma virus or human papillomavirus or hpv).tw. | 4,454 |
| 3 |  | or/1-2 | 4,471 |
| 4 | Sub-population | exp HIV/ or exp acquired immune deficiency syndrome/ | 5,860 |
| 5 |  | exp HIV Infections/ | 16,486 |
| 6 |  | (((HIV or human immunodeficien* or human immun# deficien*) adj3 (patient? or individual? or people)) or (Acquired Immunodeficiency Syndrome or AIDS)).tw. | 20,596 |
| 7 |  | or/4-6 | 29,075 |
| 8 |  | 3 and 7 | 182 |
| 9 | Epidemiology | epidemiology/ | 302 |
| 10 |  | Incidence/ | 15,442 |
| 11 |  | prevalence/ | 9,054 |
| 12 |  | mortality/ | 4,628 |
| 13 |  | morbidity/ or *disease burden/ | 3,218 |
| 14 |  | (epidemiolog* or incidence* or prevalen* or mortalit* or morbid* or burden).tw. | 321,878 |
| 15 |  | or/9-14 | 327,751 |
| 16 | Humanistic burden | quality of life/ | 43,837 |
| 17 |  | patient-reported outcome/ | 555 |
| 18 |  | patient preference/ | 1,228 |
| 19 |  | psychological well being/ | 276 |
| 20 |  | caregiver burden/ | 161 |
| 21 |  | (qol or hrqol or quality of life).tw. | 152,350 |
| 22 |  | ((patient* or caregiver* or carer* or parent* or population? or people or person* or individual*) adj3 (satisf* or perspective* or view* or prefer* or attitude* or outcome* or burden or stress* or well being or perce* or pressur* or anxi* or depress*)).tw. | 189,605 |
| 23 |  | or/16-22 | 313,020 |
| 24 | Costs and resource use | cost of illness/ | 1,657 |
| 25 |  | disease burden/ | 72 |
| 26 |  | Sick Leave/ | 706 |
| 27 |  | healthcare financing/ | 14 |
| 28 |  | productivity/ | 252 |
| 29 |  | caregiver burden/ | 161 |
| 30 |  | length of stay/ | 11,221 |
| 31 |  | health care utilization/ | 249 |
| 32 |  | ((economic or societ* or socioeconomic or socio economic or illness or disease or patient* or caregiver* or carer* or productiv* or employ*) adj3 (burden or impact or consequence?)).tw. | 22,667 |
| 33 |  | (productivity or cost* or expenditure* or expens* or financ* or visit* or inpatient* or outpatient* or hospitali* or financ* or (length adj5 stay)).tw. | 329,399 |
| 34 |  | (resource* adj2 ("use" or usage or utili* or allocat*)).tw. | 13,558 |
| 35 |  | (leave adj2 (medical or sick or disability)).tw. | 2,121 |
| 36 |  | or/24-35 | 346,637 |
| 37 | Total before any exclusion limits | 8 and 15 | 100 |
| 38 |  | 8 and 23 | 37 |
| 39 |  | 8 and 36 | 71 |
| 40 |  | or/37-39 | 128 |
| 41 | Exclusion terms | exp case study/ or exp case report/ or exp letter/ or exp editorial/ or exp preliminary communication/ or exp note/ | 595 |
| 42 |  | (exp animal/ or exp invertebrate/ or nonhuman/ or animal experiment/ or animal tissue/ or animal model/ or exp plant/ or exp fungus/) not (exp human/ or human tissue/) | 6,432 |
| 43 |  | 40 not (41 or 42) | 128 |
| 47 | Total after language limit | limit 43 to english language [Limit not valid in CDSR,ACP Journal Club,DARE,CCA,CLCMR; records were retained] | 127 |
| 48 | Total after time limit | limit 47 to yr="2012 -Current" [Limit not valid in DARE; records were retained] | 69 |
| 52 | Total after duplicates removed | remove duplicates from 51 | 65 |

### Supplementary Table 6. Summary of findings reported in the literature related to the prevalence of cervical HPV among WWH

| **Publication, Country** | **Date of data collection** | **Sample size** | **Any cervical HPV (%)** | **hrHPV (%)** | **lrHPV (%)** | **Vaccine types (%)** | **Multiple/single HPV (%)** |
| --- | --- | --- | --- | --- | --- | --- | --- |
| **Africa** | | | | | | | |
| **Adebamowo *et al.,* 2019^1^**  **Nigeria** | 2012–2014 | WWH (N=270) | NR | Any hrHPV: 32.6%  HPV16: 2.9%  HPV18: 5.5%  HPV31: 3.7%  HPV33: 4.8%  HPV35: 8.1%  HPV39: 1.8%  HPV45: 1.8%  HPV51: 2.9%  HPV52: 9.2%  HPV56: 1.8%  HPV58: 9.2%  HPV59: 2.5% | NR | NR | NR |
| **Chachage *et al.,* 2022^2^**  **Multinational Africa** | January 2015–March 2020 | WWH (N=868) | NR | Any hrHPV: 50.9%  HPV16: 13.5%  HPV35: 9.3%  HPV52: 9.4% | NR | NR | NR |
|  |  | hrHPV-positive WWH (N=442) | NR | NR | NR | NR | Single hrHPV: 57.0%  Multiple hrHPV: 43.0% |
|  |  | Women without HIV (N=134) | NR | Any hrHPV: 38.1%  HPV16: 6.0%  HPV31: 5.2%  HPV52: 6.7% | NR | NR | NR |
|  |  | hrHPV-positive women without HIV (N=51) | NR | NR | NR | NR | Single hrHPV: 78.4%  Multiple hrHPV: 21.6% |
| **Ferre *et al.,* 2019^3^**  **Togo** | August 2017–October 2017 | FSW with HIV (N=33) | NR | Any hrHPV: 48.5% | NR | NR | NR |
| **Guthrie *et al.,* 2020^4^**  **Kenya** | September 2007–December 2009 | WWH in HIV-discordant couples (N=283) | 62.0% | Any hrHPV: 47.0%  HPV16/18: 12.4%  HPV16: 6.0%  HPV18: 7.1%  HPV31: 3.9%  HPV33: 3.9%  HPV35: 8.5%  HPV39: 3.9%  HPV45: 4.2%  HPV51: 9.2%  HPV52: 20.5%  HPV56: 6.0%  HPV58: 5.7%  HPV59: 6.7%  HPV66: 7.4%  HPV68: 7.1% | Any lrHPV: 15.6% | NR | Multiple: 41.0%  Single lrHPV: 10.3%  Multiple lrHPV only: 5.3%  Single hrHPV: 19.8%  Multiple hrHPV: 26.9% |
|  |  | HPV-positive WWH (N=176) | NR | NR | NR | NR | Multiple: 66.0% |
| **Kremer *et al.,* 2018^5^**  **South Africa** | February 2013–March 2023 | WWH (N=463) | NR | Any hrHPV: 55.7% | NR | NR | Multiple: 30.2% |
| **Luckett *et al.,* 2021^6^**  **Botswana** | NR | WWH (N=237) | NR | Any hrHPV: 28.0%  HPV16: 5.0%  HPV18: 6.0%  Other hrHPV: 21.0% | NR | NR | NR |
| **Mandiriri *et al.,* 2020^7^**  **Zimbabwe** | September 2017–November 2018 | WWH (N=321) | NR | Any hrHPV: 24.9%  HPV16: 11.2%  HPV18/45: 12.5%  Other hrHPV: 65% | NR | NR | Multiple, HPV16 and other hrHPV: 5.0%  Multiple, HPV18 and other hrHPV: 6.3% |
| **Megersa *et al.,* 2023^8^**  **Southern Ethiopia** | February 2022–March 2022 | WWH (N=406) | NR | Any hrHPV: 35.2%  HPV16: 15.0%  HPV18: 6.0% | NR | NR | NR |
| **Nyasenu *et al.,* 2019^9^**  **Togo** | September 2014–September 2105 | WWH receiving cART (N=221) | 22.2% | HPV16: 1.3%  HPV18: 8.6% | NR | NR | NR |
|  |  | HPV-positive WWH (N=49) | NR | NR | NR | NR | Single: 71.4%  Multiple: 28.6% |
| **Rais *et al.,* 2023^10^**  **Algeria** | May 2018–September 2018 | WWH (N=100) | 32.0% | Any hrHPV: 32.0% | NR | NR | NR |
|  |  | hrHPV-positive WWH (N=32) | NR | HPV16: 16.0%  HPV18: 16.0%  HPV31: 12.5%  HPV33: 6.2%  HPV52: 25%  HPV58: 16.0% | Any lrHPV: 25.0%  HPV42: 9.3%  HPV54: 6.0%  CP6108: 6.0% | NR | NR |
| **Uwamungu *et al.,* 2023^11^**  **Rwanda** | January 2017–March 2018 | WWH (N=50) | NR | Any hrHPV: 24.0% | Any lrHPV: 6.0% | NR | NR |
| **Vyankandondera *et al.,* 2019^12^**  **Kenya** | NR | Pregnant WWH (N=245) | 98.4% | Any hrHPV: 84.1%  HPV16: 18.0%  HPV18: 13.5%  HPV31: 7.4%  HPV33: 4.7%  HPV35: 13.5%  HPV39: 10.8%  HPV45: 4.9%  HPV51: 26.1%  HPV52: 43.8%  HPV56: 12.2%  HPV58: 5.3%  HPV59: 0.4%  HPV68: 5.0%  HPV69: 11.8% | HPV6: 8.6%  HPV11: 4.5% | 4vHPV: 38.4% | NR |
| **Yakub *et al.,* 2019^13^**  **Nigeria** | August 2016–May 2017 | WWH (N=220) | 54.1% | HPV16: 8.2%  HPV18: 2.7%  HPV31: 0.9%  HPV33: 5.5%  HPV35: 11.8%  HPV39: 0.5%  HPV45: 7.7%  HPV51: 0.5%  HPV52: 0.4%  HPV56: 1.8% | HPV6: 4.1%  HPV11: 2.7%  HPV40: 0.5%  HPV42: 1.8%  HPV43: 1.8%  HPV44: 0.5%  HPV66: 1.4%  HPV72: 2.7%  HPV81: 2.3% | NR | Multiple: 11.4% |
| **Asia** | | | | | | | |
| **Aziz *et al.,* 2023^14^**  **Pakistan** | March 2017–August 2019 | WWH (N=65) | 36.9% | Any hrHPV: 20.0%  HPV16: 4.6%  HPV18: 6.2%  HPV33: 1.5%  HPV45: 3.1%  HPV58: 3.1%  HPV68: 1.5% | Any lrHPV: 19.92%  HPV6: 4.6%  HPV11: 6.2%  HPV54: 1.5%  HPV55: 3.1%  HPV61: 1.5% | NR | NR |
| **Isaguliants *et al.,* 2021^15^**  **Russia** | NR | WWH with tuberculosis (N=58) | NR | Any hrHPV: 58.6%  HPV16: 38%  Other hrHPV: 34.5%  HPV58: 5.2% | NR | NR | Single hrHPV: 37.9%  Multiple hrHPV: 20.7% |
| **Pahwa *et al.,* 2022^16^**  **India** | May 2010–June 2015 | WWH (N=291) | 34.4% | NR | NR | NR | NR |
| **Qiao *et al.,* 2020^17^**  **China** | May 2015–August 2015 | WWH (N=695) | NR | Any hrHPV: 25.9% | NR | NR | NR |
|  |  | hrHPV-positive WWH (N=180) | NR | HPV16: 26.1%  HPV52: 19.5%  HPV58: 21.1% | NR | NR | Single hrHPV: 69.4%  Multiple hrHPV: 30.6% |
| **Risnawati *et al.,* 2020^18^**  **Indonesia** | December 2019–March 2020 | WWH receiving ART (N=55) | 53.0% | HPV16: 3.8%  HPV18: 3.8%  HPV26: 2.5%  HPV31: 1.3%  HPV35: 1.3%  HPV39: 2.5%  HPV51: 2.5%  HPV52: 7.7%  HPV53: 3.8%  HPV58: 5.1%  HPV59: 2.5%  HPV64: 1.3%  HPV66: 5.1%  HPV68: 6.3% | HPV6: 5.1%  HPV40: 2.5%  HPV42: 3.8%  HPV43: 6.3%  HPV44: 6.3%  HPV54: 2.5%  HPV55: 2.5%  HPV56: 2.5%  HPV61: 2.5%  HPV70: 3.8%  HPV72: 2.5%  HPV81: 2.5%  HPV84: 1.3%  HPV86: 1.3% | NR | NR |
| **Wang *et al.,* 2019^19^**  **China** | March 2014–August 2014 | WWH (N=183) | NR | HPV16: 3.8%  HPV18: 3.3%  HPV31: 3.3%  HPV33: 6.0%  HPV45: 3.3%  HPV52: 14.8%  HPV58: 9.3% | HPV6: 1.6%  HPV11: 0.0%  CP8304: 2.7% | NR | NR |
| **Central and South America** | | | | | | | |
| **Abel *et al.,* 2019^20^**  **French Antilles and French Guiana** | January 2011–May 2014 | WWH (N=439) | 50.1% | Any hrHPV: 31.9% | NR | NR | NR |
| **Badial *et al.,* 2018^21^**  **Brazil** | 2010–2012 | WWH (N=40; 80 samples) | 73.8% | Any hrHPV: 57.6% | Any lrHPV: 42.4% | NR | NR |
| **Kury *et al.,* 2020^22^**  **Brazil** | NR | WWH (N=109) | 36.0% | NR | NR | NR | NR |
| **Monteiro *et al.,* 2021^23^**  **Brazil** | March 2010– December 2012 | WWH (N=169) | 63.3% | NR | NR | NR | NR |
| **Ortiz-Gutierrez *et al.,* 2021^24^**  **Mexico** | NR | WWH (N=40) | 77.5% | Any hrHPV: 37.5%  HPV16: 5.0%  HPV33: 2.5%  HPV51: 17.5%  HPV52: 2.5%  HPV56: 2.5%  HPV58: 5.0%  HPV66: 2.5% | Any lrHPV: 40.0%  HPV6: 7.5%  HPV11: 5.0%  HPV54: 2.5%  HPV70: 5.0%  HPV81: 7.5%  HPV84: 2.5%  HPV86: 2.5%  HPV90: 2.5%  HPV97: 2.5%  HPV102: 2.5% | NR | NR |
| **Perez-Quintanilla *et al.,* 2020^25^**  **Mexico** | February 2014–February 2015 | WWH (N=174) | NR | NR | NR | NR | One hrHPV: 10.0%  Two hrHPV: 14.0%  Three hrHPV: 11.0%  Four hrHPV: 13.0%  Five hrHPV: 20.0%  Six hrHPV: 11.0%  Seven hrHPV: 13.0%  Eight hrHPV: 2.0%  One lrHPV: 29.0%  Two lrHPV: 5.0%  Three lrHPV: 1.0% |
| **Teixeira *et al.,* 2018^26^**  **Brazil** | May 2014–February 2015 | WWH (N=325) | NR | Any hrHPV: 31.1% | NR | NR | NR |
|  |  | hrHPV-positive WWH (N=93) | NR | HPV16: 19.4%  HPV18: 2.2%  HPV31: 11.8%  HPV45: 12.9%  HPV51: 9.6%  HPV52: 21.5% | NR | NR | Multiple: 43.0% |
| **Tosato Boldrini *et al.,* 2021^27^**  **Brazil** | February 2014–October 2015 | WWH (N=150) | NR | Any hrHPV: 48.3% | NR | NR | NR |
| **Europe** | | | | | | | |
| **Boudes *et al.,* 2021^28^**  **France** | 2014–2020 | WWH (N=29) | 86.2% | HPV16: 14.0%  HPV18: 6.9%  HPV26: 0.0%  HPV31: 10.0%  HPV33: 6.9%  HPV35: 6.9%  HPV39: 3.4%  HPV51: 3.4%  HPV52: 14.0%  HPV58: 3.4%  HPV59: 0.0% | HPV6: 10.0%  HPV11: 0.0%  HPV40: 3.4%  HPV42: 0.0%  HPV43: 0.0%  HPV44: 0.0%  HPV45: 6.9%  HPV53: 10.0%  HPV54: 0.0%  HPV56: 3.4%  HPV61: 17.0%  HPV62: 0.0% | NR | NR |
| **Cambrea *et al.,* 2020^29^**  **Romania** | September 2018–February 2020 | WWH (N=40) | NR | HPV16: 5.3%  HPV18: 5.3%  HPV31: 42.1%  HPV39: 10.5%  HPV51: 10.5%  HPV52: 10.5%  HPV53: 15.8%  HPV56: 31.8%  HPV68: 5.3%  HPV73: 5.3% | NR | NR | NR |
| **Carriero *et al.,* 2018^30^**  **Italy** | 2009–2014 | WWH (N=48) | 23.0% | Any hrHPV: 14.6% | Any lrHPV: 6.3% | NR | NR |
| **Elliott *et al.,* 2022^31^**  **UK** | NR | WWH (N=57) | NR | Any hrHPV: 30.4% | NR | NR | NR |
|  |  | hrHPV-positive WWH (N=14) | NR | HPV16: 14.3%  HPV18/45: 0.0% |  | NR | Multiple: 75.0% |
| **Metz *et al.,* 2022^32^**  **Germany** | October 2017–August 2021; February 2021–August 2021 | Pregnant WWH (N=81) | 54.3% | Any hrHPV: 45.7%  HPV16: 25.9%  HPV18: 6.2%  HPV31: 3.7%  HPV33: 3.7%  HPV35: 8.6%  HPV39: 1.2%  HPV45: 3.7%  HPV51: 2.5%  HPV53: 3.7%  HPV56: 3.7%  HPV58: 1.2%  HPV59: 6.1%  HPV66: 8.6%  HPV68: 1.2%  HPV73: 3.7%  HPV82: 2.5% | NR | NR | Multiple: 27.2% |
| **Orlando *et al.,* 2019^33^**  **Italy** | NR | WWH (N=565) | 27.1% | Any hrHPV: 22.5%  HPV16: 8.8%  HPV16/18: 20.3%  Other hrHPV: 58.2%  HPV52: 7.7%  HPV53: 6.7%  HPV66: 7.0% | NR | NR | NR |
|  |  | HPV-positive WWH (N=153) | NR | NR | NR | 4vHPV: 16.7%  9vHPV: 40.9% | Single: 48.4%  Multiple (2–8): 51.6% |
| **Thorsteinsson *et al.,* 2018^34^**  **Denmark** | February 2011–February 2012 | WWH (N=214) | 50.5% | Any hrHPV: 28.0%  HPV16: 5.1%  HPV18: 3.3%  HPV31: 2.3%  HPV33: 3.3%  HPV35: 3.3%  HPV39: 1.9%  HPV45: 0.9%  HPV51: 5.1%  HPV52: 5.1%  HPV56: 2.8%  HPV58: 8.4%  HPV59: 0.9%  HPV68: 1.4% | Any lrHPV: 39.7%  HPV6: 4.7%  HPV11: 1.4%  HPV26: 0.0%  HPV40: 0.5%  HPV42: 4.7%  HPV43: 0.0%  HPV44: 1.4%  HPV53: 7.9%  HPV54: 2.3%  HPV61: 7.0%  HPV62: 4.2%  HPV66: 5.1%  HPV70: 5.1%  HPV71: 0.5%  HPV72: 4.2%  HPV73: 0.0%  HPV81: 5.6%  HPV82: 3.3%  HPV83: 2.3%  HPV84: 2.3%  HPV85: 0.5%  HPV89: 0.5% | 9vHPV: 73.3% | Multiple: 27.1% |
| **Middle East** | | | | | | | |
| **Al-Lawati *et al.,* 2020^35^**  **Oman** | September 2014–April 2014 | WWH (N=17) | 35.3% | NR | NR | NR | NR |
| The US and Canada | | | | | | | |
| **Al Shaarani *et al.,* 2020^36^**  **US** | July 2012–July 2015 | WWH (N=335) | 29.9% | HPV16: 16.0%  HPV18: 21.0% | NR | NR | Multiple (HPV16 and/or 18 and other): 15.0% |
| **Cameron *et al.,* 2020^37^**  **US** | 1999–2004 | WWH (n=236) | 48.7% | Any hrHPV: 43.2%  HPV16: 1.7% | Any lrHPV: 22.5% | NR | Multiple: 24.2% |
| **Covarrubias *et al.,* 2020^38^**  **US** | 2009–2012 | WWH (N=162) | NR | NR | NR | NR | Single hrHPV: 32.0%  Multiple hrHPV: 8% |
| **Liu *et al.,* 2023^39^**  **US** | 2012–2019 | WWH (N=381) | NR | Any hrHPV: 4.0%  HPV16/18: 2.0% | NR | NR | NR |
| **Kaufman *et al.,* 2022^40^**  **Canada** | January 2012–July 2016 | WWH (N=150) | NR | HPV16: 3.3% | NR | NR | NR |

Abbreviations: 4vHPV: 4-Valent HPV vaccine; 9vHPV: 9-valent HPV vaccine; ART: anti-retroviral therapy; cART: combination anti-retroviral therapy; FSW: female sex workers; HIV: human immunodeficiency virus; HPV: human papillomavirus; hr: high-risk; lr: low-risk; NR: not reported; WWH: women with HIV.

### Supplementary Table 7. Summary of comparative findings reported in the literature related to the prevalence of cervical HPV among WWH and women without HIV

| **Publication, Country** | **Date of data collection** | **Sample size** | **Any cervical HPV (%)** | **hrHPV (%)** | **lrHPV (%)** | **Vaccine types (%)** | **Multiple/single HPV (%)** |
| --- | --- | --- | --- | --- | --- | --- | --- |
| **Africa** | | | | | | | |
| **Ermel *et al.,* 2019^41^**  **Kenya** | September 2015–October 2016 | WWH (N=115) | 59.1% | Any hrHPV: 47.0%  HPV16: 10.4%  HPV18: 3.5%  HPV68: 7.0% | Any lrHPV: 32.2% | NR | Multiple: 33.0%  Multiple hrHPV: 20.0% |
|  |  | Women without HIV (N=105) | 35.2% | Any hrHPV: 27.6%  HPV16: 2.9%  HPV18: 1.0%  HPV68: 0.0% | Any lrHPV: 17.1% | NR | Multiple: 10.5%  Multiple hrHPV: 6.7% |
| **Hopkins *et al.,* 2021^42^**  **South Africa** | June 2018–March 2019 | WWH (N=57) | NR | Any hrHPV: 59.7%  HPV16: 14.0%  HPV18: 3.5%  HPV16/18: 17.5%  Non-HPV16/18 hrHPV: 56.1% | NR | NR | Multiple, HPV16 and other hrHPV: 12.3%  Multiple, HPV18 and other hrHPV: 1.8% |
|  |  | Women without HIV (N=223) | NR | Any hrHPV: 37.2%  HPV16: 8.1%  HPV18: 4.5%  HPV16/18: 12.6%  Non-HPV16/18 hrHPV: 32.7% | NR | NR | Multiple, HPV16 and other hrHPV: 4.9%  Multiple, HPV18 and other hrHPV: 3.1% |
| **Jary *et al.*, 2021^43^**  **Mali** | May–June 2018 | WWH (N=44) | NR | Any hrHPV: 77.0%  HPV31: 31.0%  HPV35: 36.0%  HPV51: 28.0%  HPV52: 28.0%  HPV56: 28.0% | NR | NR | Multiple hrHPV: 55.0% |
|  |  | Women without HIV (N=100) | NR | Any hrHPV: 55.0%  HPV31: 41.0%  HPV52: 22.0%  HPV56: 36.0% | NR | NR | Multiple hrHPV: 36.0% |
| **Karani *et al.,* 2020^44^**  **Kenya** | July 2018–July 2019 | HPV-infected WWH (N=42) | NR | HPV16: 16.7% | NR | NR | Multiple, HPV16 and HPV18/45: 11.9% |
|  |  | HPV-infected women without HIV (N=42) | NR | HPV16: 14.3% | NR | NR | Multiple, HPV16 and HPV18/45: 10.7% |
| **Kinotia *et al.,* 2022^45^**  **Kenya** | 2019 | WWH (N=106) | 23.0% | HPV16: 0.1% | HPV11: 2.2% | NR | Single: 19.2%  Multiple: 3.5% |
|  |  | Women without HIV (N=211) | 4.1% | NR | NR | NR | Single: 3.2%  Multiple: 1.2% |
| **Lieber *et al.*, 2018^46^**  **Liberia** | June 2018–August2018 | WWH (N=21) | 62.0% | NR | NR | NR | NR |
|  |  | Women without HIV (N=86) | 41.0% | NR | NR | NR | NR |
| **Mbuya *et al.,* 2020^47^**  **Tanzania** | 2013–2017 | WWH (N=100) | 75.0% | NR | NR | NR | NR |
|  |  | Women without HIV (N=113) | 47.0% | NR | NR | NR | NR |
| **Mchome *et al.,* 2021^48^**  **Tanzania** | August 2015–July 2016 | WWH (N=609) | NR | Any hrHPV: 33.7% | NR | NR | NR |
|  |  | Women without HIV (N=2,807) | NR | Any hrHPV: 15.6% | NR | NR | NR |
| **Njue *et al.,* 2021^49^**  **Kenya** | January 2019–December 2019 | WWH (N=161) | NR | HPV16: 0.1% | HPV11: 2.2% | NR | Single: 19.2%  Multiple: 3.5% |
|  |  | Women without HIV (N=156) | NR | NR | NR | NR | Single: 3.2%  Multiple: 1.2% |
| **Okoye *et al.,* 2020^50^**  **Nigeria** | April 2017–June 2018 | WWH (N=105) | 41.9% | NR | NR | NR | NR |
|  |  | Women without HIV (N=105) | 26.7% | NR | NR | NR | NR |
| **Omire *et al.,* 2020^51^**  **Kenya** | NR | WWH (N=29) | 48.3% | NR | NR | NR | Multiple: 10.3%  Single hrHPV: 10.3%  Single lrHPV: 3.4%  Multiple hrHPV: 24.1% |
|  |  | Women without HIV (N=188) | 28.7% | NR | NR | NR | Multiple: 4.3%  Single hrHPV: 8.5%%  Single lrHPV: 5.3%  Multiple hrHPV: 3.7% |
| **Swai *et al.,* 2020^52^**  **Tanzania** | August 2015–October 2017 | WWH (N=604) | NR | Any hrHPV: 33.9% | NR | NR | NR |
|  |  | Women without HIV (N=2,786) | NR | Any hrHPV: 15.8% | NR | NR | NR |
| **Sweet *et al.,* 2020^53^**  **Kenya** | August 2009–March 2011 | FSW with HIV (N=84) | 32.1% | Any hrHPV: 27.4%  HPV52: 14.3% | Any lrHPV: 4.8% | NR | NR |
|  |  | FSW without HIV (N=264) | 20.8% | Any hrHPV: 18.2%  HPV52: 8.7% | Any lrHPV: 2.7% | NR | NR |
| **Taku *et al.,* 2021^54^**  **South Africa** | September 2017–August 2018 | WWH (N=155) | NR | Any hrHPV: 40.7% | NR | NR | NR |
|  |  | Women without HIV (N=262) | NR | Any hrHPV: 21.4% | NR | NR | NR |
| **Asia** | | | | | | | |
| **Jain *et al.,* 2021^55^**  **India** | NR | WWH (N=50) | 48.0% | HPV16: 50.0%  HPV18: 37.5% | NR | NR | NR |
|  |  | Women without HIV (N=50) | 12.0% | HPV16: 83.3%  HPV18: 16.7% | NR | NR | NR |
| **Peyyela *et al.,* 2021^56^**  **India** | December 2016–October 2018 | WWH (N=110) | NR | Any hrHPV: 20.9% | NR | NR | NR |
|  |  | Women without HIV (N=110) | NR | Any hrHPV: 3.6% | NR | NR | NR |
| **Central and South America** | | | | | | | |
| **Suehiro *et al.,* 2020^57^**  **Brazil** | September 2017–May 2018 | WWH (N=115) | 44.4% | Any hrHPV: 20.0%  HPV18: 14.8%  HPV45: 14.8%  HPV58: 14.8% | Any lrHPV: 32.2%  HPV6: 17.5% | NR | Multiple: 24.3% |
|  |  | Women without HIV (N=139) | 37.4% | Any hrHPV: 23.7%  HPV16: 11.9%  HPV18: 14.2%  HPV68: 11.9% | Any lrHPV: 20.9%  HPV54: 16.1%  HPV70: 16.1%  HPV81: 29.0% | NR | Multiple: 20.9% |
| **Europe** | | | | | | | |
| **Nijhawan *et al.,* 2023^58^**  **Italy** | NR | WWH (N=24) | 62.5% | Any hrHPV: 46.0%  hrHPV only: 25.0% | lrHPV only: 17.0% | NR | hr and lrHPV: 21.0% |
|  |  | Women without HIV (N=34) | 29.0% | Any hrHPV: 27.0%  hrHPV only: 15.0% | lrHPV only: 3.0% | NR | hr and lrHPV: 12.0% |
| **The US and Canada** | | | | | | | |
| **Castle *et al., 2*022^59^**  **US** | January 2003–February 2021 | WWH (N=608) | 20.2% | NR | NR | NR | NR |
|  |  | Women without HIV (N=67,488) | 6.5% | NR | NR | NR | NR |
| **Klein *et al.,* 2020^60^**  **US** | January 2003–April 2019 | WWH (N=70) | NR | Any hrHPV: 43% | NR | NR | NR |
|  |  | Women without HIV (N=70) | NR | Any hrHPV: 19% | NR | NR | NR |

Abbreviations: FSW: Female sex workers; HIV: human immunodeficiency virus; HPV: human papillomavirus; hr: high-risk; lr: low-risk; WWH: women with HIV.

### Supplementary Table 8. Summary of findings reported in the literature related to the incidence of cervical HPV

| **Publication, Country** | **Date of data collection** | **Median time to follow-up from baseline** | **Sample size** | **Incidence** |
| --- | --- | --- | --- | --- |
| **Luckett *et al.,* 2021^6^**  **Botswana** | NR | 13 months (range: 12–15 months) | WWH (N=300) | Any hrHPV: 9.0%  HPV16: 2.0%  HPV18: 1.0%  Non-HPV16/18: 8.0% |
| **Mchome *et al.,* 2021^61^**  **Tanzania** | August 2015–September 2016 | 17.3 months (range: NR) | WWH (N=340) | Any hrHPV: 75.2 per 1,000 person-years  HPV16: 14.2 per 1,000 person-years  HPV18: 4.1 per 1,000 person-years  HPV31: 2.0 per 1,000 person-years  HPV33: 0.0 per 1,000 person-years  HPV35: NR  HPV39: 4.1 per 1,000 person-years  HPV45: 8.1 per 1,000 person-years  HPV51: 4.5 per 1,000 person-years  HPV52: 14.2 per 1,000 person-years  HPV56: 10.2 per 1,000 person-years  HPV58: 8.1 per 1,000 person-years  HPV59: 4.1 per 1,000 person-years  HPV68: 2.0 per 1,000 person-years |
|  |  |  | Women without HIV (N=1,913) | Any hrHPV: 50.9 per 1,000 person-years  HPV16: 4.9 per 1,000 person-years  HPV18: 2.8 per 1,000 person-years  HPV31: 3.8 per 1,000 person-years  HPV33: 1.7 per 1,000 person-years  HPV35: 5.2 per 1,000 person-years  HPV39: 2.1 per 1,000 person-years  HPV45: 4.2 per 1,000 person-years  HPV51: 6.1 per 1,000 person-years  HPV52: 9.4 per 1,000 person-years  HPV56: 3.5 per 1,000 person-years  HPV58: 4.2 per 1,000 person-years  HPV59: 4.9 per 1,000 person-years  HPV68: 4.2 per 1,000 person-years |
| **Murenzi *et al.,* 2021^62^**  **Rwanda** | NR | 22 months (range: NR) | WWH (N=298) | hrHPV: 6.7%  HPV16: 4.7%  HPV18: 1.7%  HPV31: 1.7%  HPV33: 1.7%  HPV35: 2.0%  HPV39: 2.0%  HPV45: 1.7%  HPV51: 2.0%  HPV52: 4.0%  HPV56: 2.4%  HPV58: 2.0%  HPV59: 1.7%  HPV66: 1.3%  HPV68: 2.0% |
| **Orlando *et al.,* 2019^33^**  **Italy** | NR | 33 months (IQR: 20–40 months) | WWH (N=412) | Any HPV: 37.4% |
|  |  |  | WWH with incident cervical HPV (N=154) | Any hrHPV: 69.6%  HPV16: 8.9%  HPV16/18: 17.9%  HPV31: 5.0%  HPV52: 5.0%  HPV53: 9.3%  HPV66: 7.0%  4vHPV: 19.5%  9vHPV: 40.9% |
| **Sweet *et al.,* 2020^53^**  **Kenya** | August 2009–March 2011 | 368 days (range: 327–501 days) | WWH (N=84) | Any HPV: 46.7 per 100,000 person-months  Any lrHPV: 19.6 per 100,000 person-months  HPV6: 0.0 per 100,000 person-months  HPV11: 5.3 per 100,000 person-months  HPV44: 1.7 per 100,000 person-months  HPV53: 1.7 per 100,000 person-months  HPV54: 7.7 per 100,000 person-months  HPV70: 0.0 per 100,000 person-months  HPV74: 0.0 per 100,000 person-months  Any hrHPV: 29.3 per 100,000 person-months  HPV16: 7.2 per 100,000 person-months  HPV18: 1.8 per 100,000 person-months  HPV31: 5.6 per 100,000 person-months  HPV33: 0.0 per 100,000 person-months  HPV35: 3.7 per 100,000 person-months  HPV39: 0.0 per 100,000 person-months  HPV45: 0.0 per 100,000 person-months  HPV51: 1.8 per 100,000 person-months  HPV52: 8.0 per 100,000 person-months  HPV56: 0.0 per 100,000 person-months  HPV58: 0.0 per 100,000 person-months  HPV66: 1.8 per 100,000 person-months |
|  |  |  | Women without HIV (N=207) | Any HPV: 27.6 per 100,000 person-months  Any lrHPV: 10.9 per 100,000 person-months  HPV44: 0.5 per 100,000 person-months  HPV53: 0.5 per 100,000 person-months  HPV54: 5.7 per 100,000 person-months  HPV70: 0.5 per 100,000 person-months  HPV74: 1.1 per 100,000 person-months  HPV6: 2.1 per 100,000 person-months  HPV11: 0.5 per 100,000 person-months  Any hrHPV: 22.8 per 100,000 person-months  HPV16: 2.1 per 100,000 person-months  HPV18: 0.0 per 100,000 person-months  HPV31: 3.8 per 100,000 person-months  HPV33: 1.1 per 100,000 person-months  HPV35: 1.6 per 100,000 person-months  HPV39: 0.5 per 100,000 person-months  HPV45: 2.7 per 100,000 person-months  HPV51: 3.9 per 100,000 person-months  HPV52: 5.4 per 100,000 person-months  HPV56: 1.1 per 100,000 person-months  HPV58: 1.1 per 100,000 person-months  HPV66: 1.1 per 100,000 person-months |
| **Videla *et al.,* 2019^63^**  **Spain** | March 1999–January 2003 | 13.2 years (range: 7.4–17.1 years) | WWH (N=67) | Any HPV: 2.0 per 100 person-years |
| **Vyankandondera *et al.,* 2019^12^**  **Kenya** | NR | 3 months after childbirth (range: NR) | Pregnant WWH (N=245) | HPV6: 7.1%  HPV11: 1.7%  Any hrHPV: 62.5%  HPV16: 9.5%  HPV18: 4.3%  HPV31: 2.2%  HPV33: 1.9%  HPV35: 7.1%  HPV39: 3.0%  HPV45: 0.9%  HPV51: 11.1%  HPV52: 19.0%  HPV56: 4.7%  HPV58: 2.2%  HPV59: 0.4%  HPV68: 6.1%  HPV69: 4.2%  4vHPV: 18.4% |

Abbreviations: CIN: Cervical intraepithelial neoplasia; HIV: human immunodeficiency virus; HPV: human papillomavirus; hr: high-risk; ICC: invasive cervical cancer; IQR: interquartile range; lr: low-risk; WWH: women with HIV.

### Supplementary Table 9. Summary of findings reported in the literature related to the prevalence of anal HPV genotypes among men and women with HIV

| **Publication, country** | **Date of data collection** | **Population/sample size** | **Any anal HPV (%)** | **hrHPV (%)** | **lrHPV (%)** | **Vaccine type (%)** | **Multiple/single HPV (%)** |
| --- | --- | --- | --- | --- | --- | --- | --- |
| **Africa** | | | | | | | |
| **Chinyowa *et al.,* 2018^64^**  **Zimbabwe** | November 2014–June 2015 | PWH (N=122) | 44.3% | Any hrHPV: 20.5% | Any lrHPV: 19.7% | NR | Multiple hrHPV: 7.4% |
|  |  | MWH (N=49) | 20.4% | Any hrHPV: 10.2% | Any lrHPV: 10.2% | NR | Multiple hrHPV: 2.0% |
|  |  | WWH (N=73) | 60.3% | Any hrHPV: 27.4% | Any lrHPV: 26.0% | NR | Multiple hrHPV: 11.0% |
| **Uwamungu *et al.,* 2023^11^**  **Rwanda** | NR | MWH in a HIV-concordant relationship (N=50) | NR | Any hrHPV: 2.0% | Any lrHPV: 2.0% | NR | NR |
|  |  | WWH in a HIV-concordant relationship (N=50) | NR | Any hrHPV: 24.0% | Any lrHPV: 2.0% | NR | NR |
| **Europe** | | | | | | | |
| **Cavallari *et al.,* 2023^65^**  **Italy** | NR | WWH (N=26) | NR | 34.0% | 26.0% | NR | NR |
|  |  | Women without HIV (N=74) | NR | 14.0% | 45.0% | NR | NR |
| **Hidalgo-Tenorio *et al.,* 2023^66^**  **Spain** | NR | PWH (N=300) | 82.6% | Any hrHPV: 67.3%  HPV16: 14.7% | Any lrHPV: 59.8% | NR | NR |
| **Squillace *et al.,* 2021^67^**  **Italy** | March 2010–January 2019 | PWH (N=354) | 82.2% | HPV16/18: 42.8% | NR | NR | HPV16 and 18 co-infection: 7.2% |
|  |  | MSM with HIV (N=174) | 90.2% | Any hrHPV: 91.0%  HPV16/18: 46.5% | NR | 9vHPV: 82.4% | HPV16 and 18 co-infection: 9.6% |
|  |  | MSW with HIV (N=90) | 76.7% | Any hrHPV: 77.0%  HPV16/18: 31.9% | NR | 9vHPV: 77.0% | HPV16 and 18 co-infection: 2.9% |
|  |  | WWH (N=61) | 68.9% | Any hrHPV: 85.0%  HPV16/18: 43.9% | NR | 9vHPV: 58.3% | HPV16 and 18 co-infection: 4.9% |
| **North America** | | | | | | | |
| **Sigel *et al.,* 2022^68^**  **US** | January 2014–August 2020 | PWH <35 years (N=1,389) | NR | Any hrHPV: 85.0%  HPV16/18: 43.0% | NR | NR | NR |
| **Central and South America** | | | | | | | |
| **Conde-Ferraez *et al.,* 2023^69^**  **Mexico** | NR | HPV-positive PWH (N=112) | NR | HPV16: 14.7%  HPV18: 13.7%  HPV33: 35.3%  HPV45: 17.6%  HPV51: 13.7%  HPV66: 18.6% | HPV6/11: 36.3%  HPV24: 16.7%  HPV43: 22.5%  HPV44: 14.7% | NR | Multiple HPV: 80.2% |
| **Boldrini *et al.*, 2018^70^**  **Brazil** | March 2013– February 2016 | PWH (N=223) | 68.6% | Any hrHPV: 50.7% | NR | NR | NR |
|  |  | MWH (N=80) | 71.3% | NR | NR | NR | NR |
|  |  | WWH (N=143) | 67.1% | NR | NR | NR | NR |
| **Medina-Laabes *et al.,* 2018^71^**  **Puerto Rico** | June 2015–December 2017 | PWH (N=239) | NR | Any hrHPV: 78.7%  HPV16: 28.0%  HPV18: 16.3%  Other hrHPV: 73.0% | NR | NR | NR |
| **Posada *et al.,* 2018^72^**  **Colombia** | June 2016–December 2018 | PWH (N=300) | NR | Any hrHPV: 82.7%  HPV16: 32.7%  HPV18: 21.7%  Other hrHPV: 78.3% | NR | NR | ≥3 HPV: 10.3% |
|  |  | MWH (N=278) | NR | Any hrHPV: 83.8%  HPV16: 33.5%  HPV18: 22.3%  Other hrHPV excluding HPV16/18: 79.9% | NR | NR | ≥3 HPV: 10.8% |
|  |  | WWH (N=22) | NR | Any hrHPV: 68.2%  HPV16: 22.7%  HPV18: 13.6%  Other hrHPV: 59.1% | NR | NR | ≥3 HPV: 4.6% |

Abbreviations: 9v: Nine-valent; HIV: human immunodeficiency virus; HPV: human papillomavirus; hr: high-risk; lr: low-risk; MSM: men who have sex with men; MWH: men with HIV; PWH: people with HIV; WWH: women with HIV.

### Supplementary Table 10. Summary of findings reported in the literature related to the prevalence of anal HPV genotypes among MWH

| **Publication, country** | **Date of data collection** | **Population/sample size** | **Any anal HPV (%)** | **hrHPV (%)** | **lrHPV (%)** | **Vaccine types (%)** | **Multiple/single HPV (%)** |
| --- | --- | --- | --- | --- | --- | --- | --- |
| **Asia** | | | | | | | |
| **Popova *et al.,* 2020^73^**  **Russia** | February 2018–October 2019 | MSM with HIV (N=73) | NR | Any hrHPV: 79.5% | NR | NR | NR |
|  |  | MSM without HIV (N=66) | NR | Any hrHPV: 54.5% | NR | NR | NR |
|  |  | Heterosexual MWH (N=58) | NR | Any hrHPV: 22.4% | NR | NR | NR |
|  |  | Heterosexual men without HIV (N=59) | NR | Any hrHPV: 3.4% | NR | NR | NR |
| **Cheng *et al.,* 2018^74^**  **Taiwan** | March 2011–June 2016 | MWH (N=714) | NR | HPV16: 17.5%  HPV18: 13.9%  HPV31: 7.8%  HPV33: 6.9%  HPV45: 12.9%  HPV51: 18.3%  HPV52: 20.2%  HPV53: 15.7%  HPV58: 15.1%  HPV59: 14.0% | HPV6: 19.6%  HPV11: 22.3%  HPV81: 10.6%  HPV84: 13.4% | 4vHPV: 52.7%  9vHPV: 70.1% | NR |
| **Liu *et al.,* 2019^75^**  **China** | August 2016–October 2017 | MWH (N=160) | 51.2% | Any hrHPV: 46.9%  HPV16: 13.1%  HPV18: 6.9%  HPV31: 8.1%  HPV33: 6.9%  HPV35: 0.6%  HPV39: 11.3%  HPV45: 3.8%  HPV51: 13.8%  HPV52: 8.1%  HPV53: 4.4%  HPV56: 1.9%  HPV58: 1.9%  HPV59: 2.5%  HPV66: 6.9%  HPV68: 5.0% | HPV6: 15.6%  HPV11: 15.0%  HPV43: 1.3%  HPV44: 1.3%  HPV81: 5.1% | NR | Multiple: 37.0% |
|  |  | Men without HIV (N=113) | 21.2% | Any hrHPV: 15.0%  HPV16: 4.4%  HPV18: 1.8%  HPV31: 0.0%  HPV33: 0.0%  HPV35: 1.8%  HPV39: 2.7%  HPV45: 1.8%  HPV51: 0.9%  HPV52: 4.4%  HPV53: 1.8%  HPV56: 0.9%  HPV58: 0.9%  HPV59: 0.0%  HPV66: 1.8%  HPV68: 1.8% | HPV6: 3.5%  HPV11: 1.8%  HPV43: 1.8%  HPV44: 1.8%  HPV81: 0.9% | NR | Multiple: 6.2% |
| **Zhang *et al.,* 2022^76^**  **China** | 2016–2019 | MWH (N=766) | 48.3% | Any hrHPV: 40.3%  HPV16: 9.4%  HPV18: 6.0%  HPV31: 5.5%  HPV33: 5.4%  HPV35: 0.9%  HPV39: 8.4%  HPV45: 2.9%  HPV51: 8.5%  HPV52: 11.2%  HPV53: 7.6%  HPV56: 1.6%  HPV58: 10.2%  HPV59: 2.4%  HPV66: 4.2%  HPV68: 4.8% | Any lrHPV: 22.6%  HPV6: 12.3%  HPV11: 8.9%  HPV42: 0.7%  HPV43: 1.7%  HPV44: 3.0%  HPV81: 1.0% | NR | Single: 16.7%  Multiple: 29.0% |
| **Central and South America** | | | | | | | |
| **Ablanedo-Terrazas *et al.,* 2018^77^**  **Mexico** | January 2014– January 2016 | MWH (N=107) | NR | Any hrHPV: 75.7% | NR | NR | NR |
| **Cuba *et al.,* 2019^78^**  **Peru** | January 2012–May 2014 | MWH (N=222) | 61.5% | NR | NR | NR | NR |
| **Europe** | | | | | | | |
| **Revollo *et al.,* 2019^79^**  **Spain** | NR | MWH (N=297) | 61.0% | NR | NR | NR | NR |
|  |  | MSM with HIV (N=147) | 77.0% | Any hrHPV: 69.0% | NR | NR | NR |
|  |  | MSW with HIV (N=105) | 29.0% | Any hrHPV: 23.0% | NR | NR | NR |
| **Santinelli *et al.,* 2020^80^**  **Italy** | NR | Caucasian MWH (N=86) | 83.7% | NR | NR | NR | NR |
|  |  | HPV-positive MWH (N=72) | NR | Any hrHPV: 51.4%  HPV16: 11.1% | HPV6: 26.4% | NR | NR |
| **Vergori *et al.,* 2018^81^**  **Italy** | February 2015–June 2016 | MWH (N=305) | 85.2% | NR | NR | NR | NR |
|  |  | HPV-positive MWH (N=260) | NR | NR | NR | NR | Multiple: 79.2% |
| **North America** | | | | | | | |
| **Nelson *et al.,* 2019**  **Canada** | 2011–2013 | MWH (N=46) | NR | Any hrHPV: 43.5% | NR | NR | NR |
|  |  | Men without HIV (N=440) | NR | Any hrHPV: 4.8% | NR | NR | NR |
| **Patel *et al.,* 2020^82^**  **US** | March 2004–June 2006 | MSM with HIV (N=325) | NR | HPV16: 36.0%  HPV18: 22.0%  HPV31: 22.0%  HPV33: 16.0%  HPV35: 19.0%  HPV39: 18.0%  HPV45: 24.0%  HPV51: 24.0%  HPV52: 21.0%  HPV56: 12.0%  HPV58: 17.0%  HPV59: 18.0%  HPV66: 14.0%  HPV68: 18.0% | NR | 9vHPV: 74.0% | NR |
|  |  | MSW with HIV (N=65) | NR | HPV16: 8.0%  HPV18: 6.0%  HPV31: 2.0%  HPV33: 0.0%  HPV35: 9.0%  HPV39: 14.0%  HPV45: 5.0%  HPV51: 6.0%  HPV52: 6.0%  HPV56: 5.0%  HPV58: 6.0%  HPV59: 7.0%  HPV66: 5.0%  HPV68: 11.0% | NR | 9vHPV: 25.0% | NR |
| **Patel *et al.,* 2018^83^**  **US** | March 2004–June 2006 | MSM with HIV (N=403) | 95.0% | Any hrHPV: 85.0%  HPV16: 38.0%  HPV18: 24.0%  HPV31: 22.0%  HPV33: 14.0%  HPV35: 18.0%  HPV39: 20.0%  HPV45: 24.0%  HPV51: 24.0%  HPV52: 22.0%  HPV56: 12.0%  HPV58: 17.0%  HPV59: 19.0%  HPV66: 15.0%  HPV68: 16.0%  HPV73: 14.0% | HPV6: 29.0%  HPV11: 16.0%  HPV26: 3.0%  HPV40: 4.0%  HPV42: 18.0%  HPV53: 22.0%  HPV54: 17.0%  HPV55: 18.0%  HPV61: 18.0%  HPV62: 20.0%  HPV64: 1.0%  HPV67: 5.0%  HPV69: 6.0%  HPV70: 16.0%  HPV71: <1.0%  HPV72: 8.0%  HPV81: 8.0%  HPV82: 7.0%  HPV83: 10.0%  HPV84: 25.0%  HPV89: 22.0% | NR | NR |
|  |  | MSW with HIV (N=96) | 59.0% | Any hrHPV: 48.0%  HPV16: 14.0%  HPV18: 8.0%  HPV31: 5.0%  HPV33: 1.0%  HPV35: 9.0%  HPV39: 10.0%  HPV45: 7.0%  HPV51: 9.0%  HPV52: 6.0%  HPV56: 5.0%  HPV58: 9.0%  HPV59: 7.0%  HPV66: 3.0%  HPV68: 9.0%  HPV73: 3.0% | HPV6: 11.0%  HPV11: 4.0%  HPV26: 0.0%  HPV40: 3.0%  HPV42: 4.0%  HPV53: 3.0%  HPV54: 5.0%  HPV55: 7.0%  HPV61: 5.0%  HPV62: 3.0%  HPV64: 0.0%  HPV67: 3.0%  HPV69: 3.0%  HPV70: 1.0%  HPV71: 1.0%  HPV72: 5.0%  HPV81: 1.0%  HPV82: 1.0%  HPV83: 5.0%  HPV84: 10.0%  HPV89: 4.0% | NR | NR |
| **Elfituri *et al.,* 2020^84^**  **US** | November 2017 – March 2020 | MWH (N=31) | NR | Any hrHPV: 84.0%  HPV16: 7.0%  HPV18: 6.0%  Other hrHPV: 45.0% | NR | NR | NR |

Abbreviations: 4v: Four-valent; 9v: nine-valent; HIV: human immunodeficiency virus; HPV: human papillomavirus; hr: high-risk; lr: low-risk; MSM: men who have sex with men; MSW: men who have sex with women; MWH: men with HIV.

### Supplementary Table 11. Summary of findings reported in the literature related to the prevalence of anal HPV genotypes among MSM with HIV

| **Publication, country** | **Date of data collection** | **Population/sample size** | **Any anal HPV (%)** | **hrHPV (%)** | **lrHPV (%)** | **Vaccine types (%)** | **Multiple/single HPV (%)** |
| --- | --- | --- | --- | --- | --- | --- | --- |
| **Africa** | | | | | | | |
| **Bouassa *et al.,* 2018^85^**  **Central African Republic** | NR | MSM with HIV (N=29) | 72.4% | Any hrHPV: 62.1%  HPV16: 13.8%  HPV18: 10.3%  HPV31: 10.3%  HPV33: 3.4%  HPV45: 13.8%  HPV52: 3.4%  HPV58: 13.8% | Any lrHPV: 48.2%  HPV6: 6.9%  HPV11: 3.4% | NR | Multiple: 65.5% |
|  |  | MSM without HIV (N=13) | 61.5% | hrHPV: 46.2%  HPV16: 0.0%  HPV18: 0.0%  HPV31: 15.4%  HPV33: 7.8%  HPV45: 0.0%  HPV52: 0.0%  HPV58: 15.4% | Any lrHPV: 46.2% | 4vHPV: 0.0%  9vHPV: 38.5% | Multiple: 46.2%  Multiple 9vHPV: 15.4% |
| **Ferre *et al.,* 2019^3^**  **Togo** | August 2017–October 2017 | MSM with HIV (N=54) | 88.9% | hrHPV: 85.2%  HPV16: 26.0%  HPV18: 18.5%  HPV35: 33.3% | HPV6: 50.0%  HPV42: 44.4% | NR | Multiple: 85.2% |
|  |  | MSM without HIV (N=153) | 31.9% | hrHPV: 30.7% | NR | NR | Multiple: 28.7% |
| **Ferre *et al.,* 2023^86^**  **Togo** | June 2021–December 2021 | MSM with HIV (N=100) | NR | hrHPV: 84.0%  HPV16/18: 27.0%  HPV35: 24.0%  HPV52: 24.0% | HPV6/11: 40.0% | High-risk 9vHPV: 75.0% | Multiple: 69.0% |
|  |  | MSM without HIV (N=100) | NR | hrHPV: 67.7%  HPV16/18: 19.0% | HPV6/11: 32.3% | High-risk 9vHPV: 56.7% | Multiple: 55.2% |
| **Koyalta *et al.,* 2021^87^**  **Mali** | April 2019–September 2019 | MSM with HIV (N=16) | 87.5% | Any hrHPV: 75.0%  HPV16: 43.7%  HPV18: 18.7% | NR | 4vHPV: 62.5%  9vHPV: 56.2% | Multiple: 81.3%  Multiple hrHPV: 56.3%  Multiple 4vHPV: 37.5%  Multiple 9vHPV: 56.2% |
|  |  | MSM without HIV (N=34) | 61.8% | Any hrHPV: 47.1%  HPV16: 14.7%  HPV18: 11.8% | NR | 4vHPV: 41.2%  9vHPV: 47.0% | Multiple: 44.1%  Multiple hrHPV: 20.6%  Multiple 4vHPV: 17.6%  Multiple 9vHPV: 35.3% |
| **Murenzi *et al.,* 2020^88^**  **Rwanda** | March 2016–October 2017 | MSM with HIV (N=67) | NR | Any hrHPV: 19.7%  HPV16: 10.6%  HPV18: 6.1%  HPV31: 3.0%  HPV33: 1.5%  HPV35: 6.1%  HPV39: 3.0%  HPV45: 0.0%  HPV51: 3.0%  HPV52: 1.5%  HPV53: 6.1%  HPV56: 4.6%  HPV58: 3.0%  HPV59: 3.0%  HPV66: 4.6%  HPV68: 1.5% | NR | High-risk 9vHPV: 16.7% | NR |
|  |  | MSM without HIV (N=278) | NR | Any hrHPV: 21.0%  HPV16: 5.9%  HPV18: 1.5%  HPV31: 2.6%  HPV33: 1.1%  HPV35: 2.2%  HPV39: 1.5%  HPV45: 1.1%  HPV51: 2.9%  HPV52: 1.5%  HPV53: 2.9%  HPV56: 0.7%  HPV58: 2.2%  HPV59: 1.5%  HPV66: 4.8%  HPV68: 2.9% | NR | High risk 9vHPV: 11.8% | NR |
| **Nyitray *et al.,* 2022^89^**  **Tanzania** | 2011–2012 | MSM with HIV (N=39) | NR | Any hrHPV: 89.7%  HPV16: 35.9% | NR | NR | NR |
|  |  | MSM without HIV (N=77) | NR | Any hrHPV: 36.4%  HPV16: 7.8% | NR | NR | NR |
| **Oo *et al.,* 2023^90^**  **Kenya** | 2016 | MSM with HIV (N=51) | 84.3% | Any hrHPV: 51.0%  HPV16: 15.7%  HPV18: 3.9% | Any lrHPV: 49.0% | 4vHPV: 48.8%  9vHPV: 62.8% | Multiple: 35.3%  Multiple hrHPV: 19.6%  Multiple 4vHPV: 2.3%  Multiple 9vHPV: 11.6% |
|  |  | MSM without HIV (N=61) | 24.6% | Any hrHPV: 16.4%  HPV16: 3.3%  HPV18: 0.0% | Any lrHPV: 13.1% | 4vHPV: 33.3%  9vHPV: 60.0% | Multiple: 14.8%  Multiple hrHPV: 1.6%  Multiple 4vHPV: 13.3%  Multiple 9vHPV: 13.3% |
| **Asia** | | | | | | | |
| **Beliakov *et al.*, 2021^91^**  **Russia** | November 2018–October 2019 | MSM with HIV (N=60) | 66.7% | Any hrHPV: 73.3%  HPV16: 31.7%  HPV18: 33.3%  HPV33: 21.7%  HPV39: 18.3%  HPV58: 18.3% | NR | NR | Multiple HPV infections: 43.3%  Single HPV infection: 23.3% |
|  |  | MSM without HIV (N=60) | NR | Any hrHPV: 61.7%  HPV16: 13.3%  HPV18: 5.0%  HPV33: 8.3%  HPV39: 6.7%  HPV58: 3.3% | NR | NR | NR |
| **Ejaz *et al.,* 2021^92^**  **Pakistan** | March 2016–November 2017 | HPV-positive MSM with HIV and TGW with HIV (N=194) | 58.8% | HPV16: 44.7%  HPV18: 26.3%  HPV31: 7.5%  HPV33: 11.4%  HPV35: 21.9%  HPV45: 5.3%  HPV52: 3.5%  HPV56: 7.0%  HPV58: 11.4%  HPV59: 8.8% | HPV6/11: 36.8% | 4vHPV: 82.4%  9vHPV: 92.1% | NR |
| **Ejaz *et al.,* 2021^93^**  **Pakistan** | March 2016–November 2017 | MSM with HIV and TGW with HIV (N=118) | NR | NR | NR | NR | Single: 22.9%  2 HPV infections: 39.8%  3 HPV infections: 20.3%  4 HPV infections: 4.3% |
| **Lin *et al.,* 2018^94^**  **Taiwan** | January 2013–December 2016 | MSM with HIV (N=68) | NR | Any hrHPV: 64.7% | Any lrHPV: 20.6% | NR | NR |
|  |  | MSM without HIV (N=15) | NR | Any hrHPV: 33.3% | Any lrHPV: 40.0% | NR | NR |
| **Shiojiri *et al.,* 2021^95^**  **Japan** | January 2019–August 2019 | MSM with HIV (N=425) | NR | Any hrHPV: 68.9%  HPV16: 19.1%  HPV18: 10.1%  HPV31: 7.5%  HPV33: 10.4%  HPV35: 8.2%  HPV39: 8.0%  HPV45: 7.5%  HPV51: 10.8%  HPV52: 25.6%  HPV56: 8.7%  HPV58: 20.0%  HPV59: 5.4%  HPV68: 7.8% | NR | NR | Multiple: 42.8% |
|  |  | HPV-positive MSM with HIV (N=293) | NR | NR | NR | 4vHPV: 28.0%  9vHPV: 84.0% |  |
|  |  | MSM without HIV (N=207) | NR | Any hrHPV: 40.6%  HPV16: 13.5%  HPV18: 3.4%  HPV31: 6.8%  HPV33: 4.3%  HPV35: 1.9%  HPV39: 2.4%  HPV45: 2.4%  HPV51: 6.3%  HPV52: 4.8%  HPV56: 2.4%  HPV58: 7.7%  HPV59: 5.8%  HPV68: 2.4% | NR | NR | Multiple: 14.0% |
|  |  | HPV-positive MSM without HIV (N=84) | NR | NR | NR | 4vHPV: 14.0%  9vHPV: 78.6% | NR |
| **Somia *et al.,* 2018^96^**  **Indonesia, Thailand, and Malaysia** | June 2013–April 2015 | MSM with HIV (N=239) | 89.8% | Any hrHPV: 76.6%  HPV16: 22.6%  HPV18: 14.2%  HPV31: 6.7%  HPV33: 7.1%  HPV35: 4.6%  HPV39: 12.1%  HPV45: 8.4%  HPV51: 13.8%  HPV52: 10.9%  HPV56: 6.3%  HPV58: 19.3%  HPV59: 16.3%  HPV66: 9.2%  HPV68: 13.8%  HPV73: 7.1% | Any lrHPV: 76.1%  HPV6: 15.1%  HPV11: 20.1%  HPV26: 4.6%  HPV40: 5.0%  HPV42: 5.4%  HPV53: 9.2%  HPV54: 6.3%  HPV55: 8.4%  HPV61: 6.3%  HPV62: 13.0%  HPV64: 0.4%  HPV67: 2.1%  HPV69: 5.4%  HPV70: 9.6%  HPV71: 2.9%  HPV72: 6.3%  HPV81: 7.5%  HPV82: 4.6%  HPV83: 3.8%  HPV84: 10.0%  CP6108: 7.5%  IS39: 2.9% | NR | lr and hrHPV: 61.1% |
|  |  | MSM without HIV (N=142) | 65.3% | Any hrHPV: 53.5%  HPV16: 11.5%  HPV18: 9.2%  HPV31: 2.8%  HPV33: 1.4%  HPV35: 1.4%  HPV39: 4.9%  HPV45: 5.6%  HPV51: 9.2%  HPV52: 12.7%  HPV56: 2.8%  HPV58: 8.5%  HPV59: 9.9%  HPV66: 2.8%  HPV68: 11.3%  HPV73: 3.5% | Any lrHPV: 43.7%  HPV6: 8.5%  HPV11: 11.3%  HPV26: 4.2%  HPV40: 0.0%  HPV42: 7.0%  HPV53: 6.3%  HPV54: 3.5%  HPV55: 4.2%  HPV61: 2.8%  HPV62: 4.2%  HPV64: 0.0%  HPV67: 0.7%  HPV69: 0.7%  HPV70: 5.6%  HPV71: 0.0%  HPV72: 1.4%  HPV81: 2.8%  HPV82: 1.4%  HPV83: 1.4%  HPV84: 3.2%  CP6108: 2.8%  IS39: 1.4% | NR | lr and hrHPV: 31.7% |
| **Wang *et al.,* 2019^97^**  **Taiwan** | 2013–2014 | MSM with HIV (N=288) | 75.0% | NR | NR | NR | NR |
| **Wu *et al.,* 2020^98^**  **Taiwan** | March 2015–July 2016 | MSM with HIV (N=101) | NR | Any hrHPV: 72.8%  HPV16: 23.5%  HPV18: 16.0%  HPV31: 1.3%  HPV33: 3.7%  HPV45: 9.9%  HPV52: 17.3%  HPV58: 18.5% | Any lrHPV: 65.4%  HPV6: 46.9%  HPV11: 4.9% | NR | NR |
|  |  | MSM without HIV (N=62) | NR | Any hrHPV: 25.9%  HPV16: 1.7%  HPV18: 1.7%  HPV31: 1.7%  HPV33: 6.9%  HPV45: 1.7%  HPV52: 6.9%  HPV58: 1.7% | Any lrHPV: 22.4%  HPV6: 13.8%  HPV11: 0.0% | NR | NR |
| **Yunihastuti *et al.,* 2020^99^**  **Multinational: Thailand, Indonesia, and Malaysia** | January 2011–November 2012 and June 2013–April 2015 | MSM with HIV (N=163) | NR | Any hrHPV: 66.5%  HPV16: 18.9%  HPV18: 13.2%  HPV31: 4.7%  HPV33: 6.1%  HPV35: 4.3%  HPV39: 8.0%  HPV45: 7.1%  HPV51: 14.2%  HPV52: 7.6%  HPV56: 4.3%  HPV58: 11.8%  HPV59: 13.7%  HPV68: 9.9% | NR | NR | NR |
|  |  | MSM without HIV (N=101) | NR | Any hrHPV: 66.5%  HPV16: 13.6%  HPV18: 9.9%  HPV31: 1.5%  HPV33: 0.0%  HPV35: 0.8%  HPV39: 6.1%  HPV45: 5.3%  HPV51: 11.4%  HPV52: 12.9%  HPV56: 1.5%  HPV58: 6.9%  HPV59: 8.3%  HPV68: 7.6% | NR | NR | NR |
| **Zhou *et al.,* 2020^100^**  **China** | January 2017–August 2017 | MSM with HIV (N=39) | 79.5% | Any hrHPV: 64.1%  HPV16: 20.5%  HPV18: 12.8%  HPV33: 10.3%  HPV35: 10.3%  HPV39: 12.8%  HPV45: 5.1%  HPV51: 12.8%  HPV52: 20.5%  HPV58: 18.0%  HPV66: 5.1%  HPV67: 7.7%  HPV68: 15.4%  HPV73: 10.3% | Any lrHPV: 53.9%  HPV6: 18.0%  HPV11: 12.8%  HPV40: 12.8%  HPV42: 12.8%  HPV43: 5.1%  HPV69: 5.1%  HPV70: 5.1%  HPV82: 5.1%  HPV84: 15.4% | 4vHPV: 41.0%  9vHPV: 53.8% | Single: 35.9%  2 infections: 20.5%  3 infections: 12.8%  ≥4 infections: 17.9% |
| **Central and South America** | | | | | | | |
| **Méndez-Martínez *et al.,* 2020^101^**  **Mexico** | NR | Anal HPV16-positive MSM with HIV (N=102) | NR | HPV16: 100% | Any lrHPV: 80.4%  HPV6: 39.2%  HPV11: 65.7% | NR | NR  HPV16 and HPV18: 50.0%  HPV16: HPV52: 56.9% |
| **Jalil *et al.,* 2021^102^**  **Brazil** | August 2015–January 2016 | TGW with HIV (N=122) | 86.1% | Any hrHPV: 71.7%  HPV16: 22.1%  HPV18: 11.5%  HPV31: 9.8%  HPV33: 11.5%  HPV35: 11.5%  HPV39: 21.3%  HPV45: 9.0%  HPV51: 13.9%  HPV52: 13.9%  HPV56: 18.0%  HPV58: 18.9%  HPV59: 14.8%  HPV68: 18.9%  HPV73: 7.4% | HPV6: 17.2%  HPV11: 9.0%  HPV40: 9.0%  HPV42: 22.1%  HPV43: 5.7%  HPV44: 32.0%  HPV53: 19.7%  HPV55: 32.0%  HPV66: 11.5%  HPV70: 13.1%  HPV82: 15.6% | 4vHPV: 45.1%  9vHPV: 66.4% | Multiple: 85.7% |
|  |  | TGW without HIV (N=150) | 71.3% | Any hrHPV: 41.1%  HPV16: 14.0%  HPV18: 1.3%  HPV31: 2.7%  HPV33: 5.3%  HPV35: 2.0%  HPV39: 8.7%  HPV45: 2.0%  HPV51: 9.3%  HPV52: 8.7%  HPV56: 8.0%  HPV58: 4.0%  HPV59: 4.7%  HPV66: 8.0%  HPV68: 11.3%  HPV73: 0.0% | HPV6: 14.7%  HPV11: 6.7%  HPV40: 4.7%  HPV42: 10.0%  HPV43: 6.0%  HPV44: 16.7%  HPV53: 7.3%  HPV55: 16.7%  HPV70: 5.3%  HPV82: 6.0% | 4vHPV: 30.7%  9vHPV: 38.7% | Multiple: 58.9% |
| **Europe** | | | | | | | |
| **Alberts *et al.*, 2020^103^**  **France** | December 2014–June 2016 | MSM with HIV (N=438) | NR | Any hrHPV: 71.0%  HPV16: 29.0%  HPV18: 12.0%  Other hrHPV: 64.0% | NR | NR | NR |
| **Biała *et al.*, 2022^104^**  **Poland** | NR | MSM with HIV (N=54) | 76.0% | Any hrHPV: 72.0%  HPV16: 22.0%  HPV18: 15.0% | 30% | NR | Multiple: 61.0% |
| **Borena *et al.,* 2019^105^**  **Austria** | May 2015–October 2016 | MSM with HIV (N=222) | 89.0% | Any hrHPV: 73.0% | NR | NR | Multiple: 75.3% |
| **Bruzzesi *et al.,* 2022^106^**  **Italy** | January 2015–December 2019 | MSM with HIV (N=1,352) | 88.0% | Any hrHPV: 79.0%  HPV16: 22.8%  HPV18: 10.9%  HPV26: 0.8%  HPV31: 17.0%  HPV33: 15.8%  HPV35: 13.3%  HPV39: 10.4%  HPV45: 8.6%  HPV51: 12.3%  HPV52: 7.2%  HPV56: 9.4%  HPV58: 24.1%  HPV59: 8.9%  HPV66: 10.5%  HPV68: 18.1%  HPV73: 13.0% | HPV6: 21.5%  HPV11: 12.6%  HPV40: 6.2%  HPV42: 24.4%  HPV43: 9.3%  HPV44: 16.6%  HPV53: 27.2%  HPV54: 10.4%  HPV61: 12.4%  HPV69: 5.5%  HPV70: 13.8%  HPV82: 8.6% | NR | NR |
| **Callejo *et al.,* 2022^107^**  **Spain** | May 2019–May 2021 | MSM with HIV (N=103) | 88.3% | Any hrHPV: 65.0%  HPV16: 27.2%  HPV18: 3.9% | Any lrHPV: 9.0% | NR | Multiple: 63.1% |
| **Combes *et al.,* 2018^108^**  **France** | December 2014–January 2016 | MSM with HIV (N=490) | NR | Any hrHPV: 70.0%  HPV16: 29.0%  HPV33: 13.3%  HPV68: 16.3% | NR | NR | Multiple: 46.7% |
| **Dona *et al.,* 2018^109^**  **Italy** | August 2009–June 2017 | MSM with HIV (N=321) | 95.0% | Any hrHPV: 77.9%  HPV16 and/or 18: 34.3% | Any lrHPV: 76.0% | NR | Multiple: 79.8% |
|  |  | MSM without HIV (N=452) | 72.8% | Any hrHPV: 51.5%  HPV16 and/or 18: 23.0% | Any lrHPV: 51.1% | NR | Multiple: 49.1% |
| **Giuliani *et al.,* 2018^110^**  **Italy** | August 2009–March 2016 | MSM with HIV (N=313) | 95.2% | HPV16: 26.5% | HPV6: 21.7% | 4vHPV: 49.5%  9vHPV: 71.2% | Multiple: 79.5%  1 9vHPV: 34.5%  2 9vHPV: 22.0%  3 9vHPV: 9.6%  4 9vHPV: 2.9%  5 9vHPV: 2.2%  ≥6 9vHPV: 0.0% |
| **Hidalgo-Tenorio *et al.,* 2019^111^ and Hidalgo-Tenorio *et al*., 2021^112^**  **Spain** | May 2010–December 2018 | MSM with HIV (N=405) | NR | Any hrHPV: 76.9%  HPV16: 27.7%  HPV18: 12.9%  HPV26: 1.5%  HPV31: 14.0%  HPV33: 7.4%  HPV35: 9.1%  HPV39: 11.7%  HPV45: 12.7%  HPV51: 14.0%  HPV52: 12.7%  HPV56: 7.9%  HPV58: 5.8%  HPV59: 10.7%  HPV66: 8.6%  HPV68: 10.7%  HPV73: 9.4% | Any lrHPV: 73.1%  HPV6: 18.0%  HPV11: 18.0%  HPV12: 0.3%  HPV40: 1.8%  HPV42: 18.3%  HPV43: 2.5%  HPV48: 0.3%  HPV53: 9.1%  HPV54: 6.6%  HPV55: 16.2%  HPV61: 7.6%  HPV62: 14.2%  HPV64: 0.3%  HPV69: 3.6%  HPV70: 8.1%  HPV71: 0.3%  HPV72: 7.1%  HPV81: 12.9%  HPV82: 4.3%  HPV83: 1.3%  HPV84: 7.6%  HPV89: 0.3%  CP6108: 3.3% | NR | hr and lrHPV: 58.1% |
| **Marra *et al.,* 2019^113^**  **The Netherlands** | 2010–2011 | MSM with HIV (N=193) | 87.6% | HPV16: 37.0%  HPV18: 24.0%  HPV31: 40.0%  HPV33: 20.0%  HPV45: 23.0%  HPV52: 40.0%  HPV58: 12.0% | HPV6: 34.0%  HPV11: 17.0% | NR | NR |
| **Nemcova *et al.,* 2022^114^**  **Czech Republic** | May 2017–December 2020 | MSM with HIV (N=172) | 97.7% | Any hrHPV: 76.0%  HPV16: 24.4% | Any lrHPV: 12.8% | NR | Multiple: 82.5% |
|  |  | MSM without HIV (N=17) | 88.2% | Any hrHPV: 52.9%  HPV16: 35.3% | Any lrHPV: 36.4% | NR | Multiple: 58.8% |
| **Parisi *et al.,* 2019^115^**  **Italy** | January 2013–December 2013 | MSM with HIV (N=165) | 89.1% | Any hrHPV: 65.4%  HPV16: 12.7%  HPV18: 17.0%  HPV31: 8.5%  HPV33: 5.5%  HPV45: 10.9%  HPV52: 15.2%  HPV58: 3.0% | Any lrHPV: 23.6% | NR | NR |
| **Rollo *et al.,* 2021**  **Italy** | NR | MSM with HIV (N=85) | 94.1% | Any hrHPV: 80.0%  HPV16: 37.6% | NR | NR | NR |
|  |  | MSM without HIV (N=76) | 78.9% | Any hrHPV: 63.1%  HPV16: 22.4% | NR | NR | NR |
| **Szab****ó *et al.,* 2018^116^**  **Hungary** | NR | MSM with HIV (N=80) | 97.5% | Any hrHPV: 88.8%  HPV16: 42.5%  HPV18: 22.5%  HPV31: 6.3%  HPV33: 7.5%  HPV35: 5.0%  HPV39: 6.3%  HPV45: 13.8%  HPV51: 21.3%  HPV52: 23.8%  HPV56: 1.3%  HPV58: 11.3%  HPV59: 22.5%  HPV68: 11.3%  HPV73: 15.0% | Any lrHPV: 75.0%  HPV6: 23.8%  HPV11: 16.3%  HPV40: 2.5%  HPV42: 1.3%  HPV53: 12.5%  HPV54: 6.3%  HPV55: 18.8%  HPV61: 10.0%  HPV62: 15.0%  HPV69: 3.8%  HPV70: 8.8%  HPV71: 1.3%  HPV72: 5.0%  HPV81: 5.0%  HPV82: 3.8%  HPV83: 3.8%  HPV84: 17.5%  CP6108: 23.8%  IS39: 1.3% | NR | Only lrHPV: 5.0%  Only hrHPV: 20.0%  lr and hrHPV: 67.5%  1 infection: 15.0%  2 infections: 17.5%  3 infections: 17.5%  4 infections: 13.7% |
|  |  | MSM without HIV (N=12) | 58.3% | Any hrHPV: 33.3%  HPV16: 25.0%  HPV18: NR  HPV31: NR  HPV33: 8.3%  HPV45: NR  HPV52: NR  HPV58: NR  HPV59: 8.3%  HPV73: 8.3% | Any lrHPV: 58.3%  HPV6: 8.3%  HPV11: 8.3%  HPV42: 8.3%  HPV53: 16.7%  HPV54: 8.3%  HPV61: 25.0%  HPV70: 8.3%  HPV83: 8.3%  HPV84: 25.0%  CP6108: 8.3% | NR | Only lrHPV: 16.7%  Only hrHPV: 0.0%  lr and hrHPV: 33.3%  1 infection: 8.3%  2 infections: 8.3%  3 infections: 33.3%  4 infections: 0.0% |
| **Ucciferri *et al.,* 2018^117^**  **Italy** | NR | MSM with HIV (N=45) | 96.3% | NR | NR | NR | NR |
|  |  | MSM without HIV (N=45) | 70.6% | NR | NR | NR | NR |
| **Oceania** | | | | | | | |
| **Ong *et al.,* 2019^118^**  **Australia** | 2013–2014 | MSM with HIV (N=255) | 74.0% | Any hrHPV: 55.0%  HPV16: 23.9%  HPV18: 8.2%  HPV31: 5.9%  HPV33: 3.1%  HPV45: 11.4%  HPV51: 8.6%  HPV52: 7.8%  HPV56: 4.3%  HPV58: 10.6%  HPV59: 11.0%  HPV68: 8.2% | NR | NR | NR |
|  |  | HPV-positive MSM with HIV (N=189) | NR | NR | NR | NR | Multiple: 83.0% |
| **Poynten *et al.,* 2021^119^**  **Australia** | 2010–2015 | GBM with HIV (N=220) | NR | Any hrHPV: 83.9%  HPV16: 35.8%  Other hrHPV: 48.2% | NR | NR | NR |
|  |  | GBM without HIV (N=397) | NR | Any hrHPV: 72.4%  HPV16: 31.9%  Other hrHPV: 40.5% | NR | NR | NR |
| **The US and Canada** | | | | | | | |
| **Bhardwaj *et al.*, 2022^120^**  **US** | 2015–2020 | MSM with HIV (N=516) | NR | hrHPV: 87.0%  HPV16/18: 44.0%  Non-16/18 HPV: 83.0% | NR | NR | NR |
| **Harfouch *et al.,* 2022^121^**  **US** | April 2021–December 2021 | TGW with HIV (N=30) | 72.0% | NR | NR | NR | NR |
|  |  | TGW without HIV (N=11) | 28.0% | NR | NR | NR | NR |

Abbreviations: 4vHPV: Four-valent HPV vaccine; 9vHPV: nine-valent HPV vaccine; GBM: gay and bisexual men; HIV: human immunodeficiency virus; HPV: human papillomavirus; hr: high-risk; HSIL: high-grade squamous intraepithelial lesion; lr: low-risk; MSM: men who have sex with men; MWH: men with HIV; TGW: transgender women.

### Supplementary Table 12. Summary of findings reported in the literature related to the prevalence of anal HPV genotypes among WWH

| **Population, country** | **Date of data collection** | **Population/sample size** | **Any anal HPV (%)** | **hrHPV (%)** | **lrHPV (%)** | **Vaccine types (%)** | **Multiple/single HPV (%)** |
| --- | --- | --- | --- | --- | --- | --- | --- |
| **Asia** | | | | | | | |
| **Gupta *et al.,* 2022^122^**  **India** | October 2019–October 2021 | WWH (N=135) | NR | Any hrHPV: 20.7% | NR | NR | NR |
|  |  | Women without HIV (N=160) | NR | Any hrHPV: 8.7% | NR | NR | NR |
| **Central and South America** | | | | | | | |
| **Rodrigues *et al.*, 2023^123^**  **Brazil** | August 2015–August 2016 | WWH (N=41) | 97.6% | NR | NR | NR | NR |
|  |  | HPV-positive WWH (N=40) | NR | Any hrHPV: 75.0%  HPV31: N=9  HPV51: N=12  HPV58: N=9  HPV59: N=10 | NR | NR | Single: 15.0%  Multiple: 85.0% |
|  |  | Women without HIV (N=112) | 31.3% | NR | NR | NR | NR |
|  |  | HPV-positive women without HIV (N=35) | NR | Any hrHPV: 42.9%  HPV16: N=6  HPV18: N=5 | NR | NR | Single: 97.0%  Multiple: 3.0% |
| **Europe** | | | | | | | |
| **Thorsteinsson *et al.,* 2018^34^**  **Denmark** | February 2011–February 2012 | WWH (N=214) | 63.1% | Any hrHPV: 39.3%  HPV16: 7.0%  HPV18: 7.0%  HPV31: 3.7%  HPV33: 4.2%  HPV35: 5.1%  HPV39: 1.4%  HPV45: 3.3%  HPV51: 9.3%  HPV52: 4.7%  HPV56: 3.3%  HPV58: 8.9%  HPV59: 4.2%  HPV68: 2.8%  HPV73: 0.0% | Any lrHPV: 46.3%  HPV6: 6.5%  HPV11: 0.9%  HPV26: 0.0%  HPV40: 1.4%  HPV42: 1.4%  HPV43: 0.0%  HPV44: 3.3%  HPV53: 13.6%  HPV54: 1.4%  HPV61: 10.3%  HPV62: 5.6%  HPV66: 6.1%  HPV70: 8.9%  HPV71: 1.4%  HPV72: 2.8%  HPV81: 5.1%  HPV82: 4.7%  HPV83: 5.1%  HPV84: 3.3%  HPV85: 1.4%  HPV89: 0.0% | 9vHPV: 69.1% | Multiple: 34.1% |
| **The US and Canada** | | | | | | | |
| **Al Shaarani *et al.,* 2020^36^**  **US** | July 2012–July 2015 | WWH (N=335) | 66.6% | HPV16: 23.3%  HPV18: 26.0%  Other hrHPV: 91.0% | NR | NR | HPV16 and/pr 18 with other types: 35.4% |
| **Kaufman *et al.,* 2022^40^**  **Canada** | January 2012–July 2015 | WWH (N=150) | NR | HPV16: 15.3% | NR | NR | NR |
| **Liu *et al.,* 2023**  **US^39^** | 2012–2019 | WWH (N=381) | NR | Any hrHPV: 35.0%  HPV16: 11.0%  HPV16/18: 19.0%  Other hrHPV: 32.0% | NR | NR | NR |

Abbreviations: 9vHPV: Nine-valent HPV vaccine; HIV: human immunodeficiency virus; HPV: human papillomavirus; hr: high-risk; HSIL: high-grade squamous intraepithelial lesion; lr: low-risk; WWH: women with HIV.

### Supplementary Table 13. Summary of findings reported in the literature related to the incidence of anal HPV infections

| **Publication, Country** | **Date of data collection** | **Median time to follow-up from baseline** | **Population/sample size** | **Incidence** |
| --- | --- | --- | --- | --- |
| **Alberts *et al.,* 2020^103^**  **France** | December 2014–June 2016 | Annual 12-month follow-ups for study duration | MSM with HIV (N=438) | HPV16: 4.4 per 1,000 person-months  Cumulative incidence of HPV16 (%):  12 months: 7.1%  24 months: 9.5%  36 months: 15.6%  HPV18: 4.3 per 1,000 person-months  Cumulative incidence of HPV18 (%):  12 months: 6.0%  24 months: 10.6%  36 months: 10.6% |
| **Dona *et al.,* 2022^124^**  **Italy** | November 2009–December 2019 | 38 months (IQR: 19–57 months) | MSM with HIV (N=204) | hrHPV: 36.1 per 1,000 person-months  HPV16: 10.2 per 1,000 person-months  HPV18: 7.2 per 1,000 person-months  lrHPV: 32.3 per 1,000 person-months  HPV6: 3.5 per 1,000 person-months  HPV11: 2.2 per 1,000 person-months |
| **Ong *et al.,* 2019^118^**  **Australia** | 2013–2014 | 1.99 years (IQR: 1.84–2.17 years) | MSM with HIV (N=255) | HPV16: 4.9 per 100 person-years  HPV18: 2.5 per 100 person-years  HPV31: 3.7 per 100 person-years  HPV33: 1.5 per 100 person-years  HPV35: 2.4 per 100 person-years  HPV45: 3.7 per 100 person-years  HPV51: 4.5 per 100 person-years  HPV52: 4.2 per 100 person-years  HPV56: 1.5 per 100 person-years  HPV58: 2.1 per 100 person-years  HPV59: 1.4 per 100 person-years  HPV68: 3.3 per 100 person-years |
| **Patel *et al.,* 2020^82^**  **US** | March 2004–June 2012 | 59 months (IQR: 51–60 months) | MSM with HIV (N=325) | HPV16: 6.0 per 100 person-years  HPV18: 3.3 per 100 person-years  HPV31: 3.1 per 100 person-years  HPV33: 3.8 per 100 person-years  HPV35: 4.8 per 100 person-years  HPV39: 4.1 per 100 person-years  HPV45: 4.0 per 100 person-years  HPV51: 4.7 per 100 person-years  HPV52: 6.1 per 100 person-years  HPV56: 3.9 per 100 person-years  HPV58: 3.8 per 100 person-years  HPV59: 4.7 per 100 person-years  HPV66: 3.9 per 100 person-years  HPV68: 3.7 per 100 person-years  9vHPV: 15.6 per 100 person-years |
|  |  | 60 months (IQR: 58–62 months) | MSW with HIV (N=65) | HPV16: 1.6 per 100 person-years  HPV18: 0.7 per 100 person-years  HPV31: 1.1 per 100 person-years  HPV33: 1.4 per 100 person-years  HPV35: 0.8 per 100 person-years  HPV39: 2.1 per 100 person-years  HPV45: 2.0 per 100 person-years  HPV51: 3.2 per 100 person-years  HPV52: 0.7 per 100 person-years  HPV56: 1.9 per 100 person-years  HPV58: 1.5 per 100 person-years  HPV59: 1.9 per 100 person-years  HPV66: 0.7 per 100 person-years  HPV68: 0.8 per 100 person-years  9vHPV: 7.6 per 100 person-years |
| **Poynten *et al.,* 2022^125^**  **Australia** | 2010–2015 | Annual 12-month follow-ups over 3 years | GBM with HIV (N=220) | hrHPV: 3.1 per 100 person-years |
|  |  |  | GBM without HIV (N=397) | hrHPV: 1.2 per 100 person-years |
| **Poynten *et al.,* 2021^119^**  **Australia** | 2010–2015 | Annual 12-month follow-ups over 3 years | GBM with HIV (N=220) | HPV16: 4.3 per 100 person-years  HPV18: 3.9 per 100 person-years  HPV31: 2.2 per 100 person-years  HPV33: 3.4 per 100 person-years  HPV35: 4.7 per 100 person-years  HPV39: 3.3 per 100 person-years  HPV45: 6.9 per 100 person-years  HPV51: 4.5 per 100 person-years  HPV52: 7.9 per 100 person-years  HPV56: 5.1 per 100 person-years  HPV58: 6.8 per 100 person-years  HPV59: 4.6 per 100 person-years  HPV68: 7.5 per 100 person-years |
|  |  |  | GBM without HIV (N=397) | HPV16: 4.4 per 100 person-years  HPV18: 3.2 per 100 person-years  HPV31: 2.6 per 100 person-years  HPV33: 1.9 per 100 person-years  HPV35: 1.7 per 100 person-years  HPV39: 3.8 per 100 person-years  HPV45: 4.3 per 100 person-years  HPV51: 4.4 per 100 person-years  HPV52: 4.0per 100 person-years  HPV56: 2.6 per 100 person-years  HPV58: 3.0 per 100 person-years  HPV59: 4.3 per 100 person-years  HPV68: 4.7 per 100 person-years |
| **Squillace *et al.,* 2021^67^**  **Italy** | March 2010–January 2019 | 3.6 years (IQR: 2.1–5.7 years) | PWH (N=354) | hrHPV: 66.7%  9vHPV: 40.0% |
| **Yunihastuti *et al.,* 2020^99^**  **Multinational (Thailand, Indonesia, and Malasia)** | January 2011–November 2012 and June 2013–April 2015 | 6-month follow-ups for 12 years | MSM with HIV and TGW with HIV (N=163) | Any hrHPV: 28.4 per 1,000 person-months  HPV16: 6.8 per 1,000 person-months  HPV18: 4.4 per 1,000 person-months  HPV31: 0.6 per 1,000 person-months  HPV33: 1.2 per 1,000 person-months  HPV35: 0.6 per 1,000 person-months  HPV39: 2.4 per 1,000 person-months  HPV45: 0.6 per 1,000 person-months  HPV51: 3.2 per 1,000 person-months  HPV52: 4.2 per 1,000 person-months  HPV56: 1.7 per 1,000 person-months  HPV58: 3.1 per 1,000 person-months  HPV59: 6.3 per 1,000 person-months  HPV68: 1.2 per 1,000 person-months |
|  |  |  | MSM without HIV and TGW without HIV (N=101) | Any hrHPV: 13.9 per 1,000 person-months  HPV16: 2.8 per 1,000 person-months  HPV18: 1.8 per 1,000 person-months  HPV31: 0.8 per 1,000 person-months  HPV33: 0.0 per 1,000 person-months  HPV35: 0.0 per 1,000 person-months  HPV39: 0.0 per 1,000 person-months  HPV45: 0.0 per 1,000 person-months  HPV51: 1.8 per 1,000 person-months  HPV52: 0.0 per 1,000 person-months  HPV56: 0.0 per 1,000 person-months  HPV58: NR  HPV59: NR  HPV68: 2.6 per 1,000 person-months |

Abbreviations: GBM: Gay and bisexual men; HIV: human immunodeficiency virus; HPV: human papillomavirus, hr: high-risk; IQR: interquartile range; MSM: men who have sex with men; PWH: people with HIV; TGW: transgender women.

### Supplementary Table 14. Summary of findings reported in the literature related to the prevalence of oral HPV among individuals living with HIV

| **Publication, Country** | **Date of data collection** | **Sample size** | **Any oral HPV (%)** | **hrHPV (%)** | **lrHPV (%)** | **HPV vaccine types (%)** | **Multiple/single HPV (%)** |
| --- | --- | --- | --- | --- | --- | --- | --- |
| **PWH** | | | | | | | |
| **Castillejos-Garcia *et al.,* 2018^126^**  **Mexico** | September 2013–February 2015 | PWH (N=97) | 55.7% | Any hrHPV: 26.8%  HPV16: 9.3%  HPV18: 24.1%  HPV33: 1.8%  HPV45: 1.8%  HPV58: 13.0%  HPV66: 1.9% | Any lrHPV: 28.9%  HPV6: 11.1%  HPV11: 1.8%  HPV13: 22.2%  HPV38: 1.8%  HPV72: 2.7% | NR | NR |
| **Hidalgo-Tenorio *et al.,* 2023^66^**  **Spain** | NR | PWH (N=300) | 13.0% | Any hrHPV: 9.7%  HPV16: 2.3%  HPV18: 0.7%  HPV26: 0.3%  HPV31: 0.3%  HPV33: 0.7%  HPV35: 0.3%  HPV39: 1.3%  HPV51: 0.3%  HPV52: 0.3%  HPV53: 0.3%  HPV56: 0.7%  HPV58: 0.3%  HPV59: 1.0%  HPV66: 0.7%  HPV68: 1.7%  HPV82: 0.3% | Any lrHPV: 5.0%  HPV6: 1.0%  HPV11: 2.3%  HPV40: 1.0%  HPV42: 0.7%  HPV43: 0.3%  HPV44: 1.3%  HPV54: 0.7%  HPV61: 0.7%  HPV62/81: 0.3%  HPV69: 0.7%  HPV70: 0.7% | NR | Simultaneous lr and hrHPV: 1.7% |
| **Uwamungu *et al.,* 2023**  **Rwanda** | NR | Concordant opposite sex couples with HIV: MWH (N=50) | NR | Any hrHPV: 10.0% | Any lrHPV: 0.0% | NR | NR |
|  |  | Concordant opposite sex couples with HIV: WWH (N=50) | NR | Any hrHPV: 12.0% | Any lrHPV: 0.0% | NR | NR |
| **MWH** | | | | | | | |
| **Ablanedo-Terrazas *et al.,* 2018^77^**  **Mexico** | January 2014– January 2016 | MWH (N=107) | NR | Any hrHPV: 9.3%  HPV16: 1.9%  HPV18: 0.0%  Non-HPV16/18: 6.7% | NR | NR | NR |
| **Biala *et al.,* 2022^104^**  **Poland** | NR | MSM with HIV (N=54) | 18.5% | Any hrHPV: 15.0%  HPV16: 3.7%  HPV18: 1.9% | Any lrHPV: 7.4% | NR | Multiple: 5.6% |
| **Callejo *et al.,* 2022^107^**  **Spain** | May 2019–May 2021 | MSM with HIV (N=103) | 13.6% | HPV16: 1.9% | NR | NR | NR |
| **Gonçalves *et al.,* 2020^127^**  **Portugal** | January 2016–April 2016 | MWH (N=255) | 17.6% | Any hrHPV: 28.6%  HPV16: 1.6%  HPV18: 1.6%  HPV26: 0.4%  HPV31: 2.4%  HPV45: 0.4%  HPV53: 1.2%  HPV56: 13.7%  HPV58: 8.2%  HPV59: 0.8%  HPV66: 7.8%  HPV68: 0.4%  HPV73: 0.4%  HPV82: 0.4% | Any lrHPV: 28.6%  HPV6: 2.7%  HPV11: 2.7%  HPV40: 0.8%  HPV43: 0.4%  HPV44: 1.2%  HPV54: 1.6%  HPV61: 7.1%  HPV70: 12.3% | NR | Single: 2.4%  Single hrHPV: 2.0%  Single lrHPV: 0.4%  Multiple: 15.3%  Multiple hrHPV: 1.6%  Multiple lrHPV: 13.7% |
|  |  | MSM with HIV (N=142) | 17.6% | Any hrHPV: 31.0%  HPV16: 2.7%  HPV18: 0.9%  HPV26: 0.0%  HPV31: 1.8%  HPV45: 0.0%  HPV53: 1.4%  HPV56: 12.7%  HPV58: 9.7%  HPV59: 0.7%  HPV66: 9.2%  HPV68: 0.7%  HPV73: 0.7%  HPV82: 0.7% | Any lrHPV: 25.7%  HPV6: 2.8%  HPV11: 2.8%  HPV40: 1.4%  HPV43: 0.7%  HPV44: 2.1%  HPV54: 2.1%  HPV61: 7.0%  HPV70: 12.0% | NR | Single: 2.7%  Single hrHPV: 1.8%  Single lrHPV: 0.9%  Multiple: 15.1%  Multiple hrHPV: 0.9%  Multiple lrHPV: 14.2% |
|  |  | MSW with HIV (N=113) | 17.7% | Any hrHPV: 26.8%  HPV16: 0.7%  HPV18: 2.1%  HPV26: 0.7%  HPV31: 2.8%  HPV45: 0.7%  HPV53: 0.9%  HPV56: 15.0%  HPV58: 7.0%  HPV59: 0.9%  HPV66: 6.2%  HPV68: 0.0%  HPV73: 0.0%  HPV82: 0.0% | Any lrHPV: 31.0%  HPV6: 2.7%  HPV11: 2.7%  HPV40: 0.0%  HPV43: 0.0%  HPV44: 0.0%  HPV54: 0.9%  HPV61: 7.1%  HPV70: 12.4% | NR | Single: 2.1%  Single hrHPV: 2.1%  Single lrHPV: 0.0%  Multiple: 15.5%  Multiple hrHPV: 2.1%  Multiple lrHPV: 13.4% |
| **Parisi *et al.,* 2019^115^**  **Italy** | January 2013–December 2013 | MSM with HIV (N=106) | 28.3% | Any hrHPV: 9.4%  HPV16: 2.8%  HPV18: 0.9%  HPV31: 0.9%  HPV33: 0.0%  HPV35: 0.9%  HPV39: 0.0%  HPV45: 0.0%  HPV51: 0.9%  HPV52: 0.0%  HPV56: 0.9%  HPV58: 0.9%  HPV59: 0.9% | NR | NR | NR |
| **Vergori *et al.,* 2018^81^**  **Italy** | February 2015–June 2016 | MWH (N=305) | 20.9% | NR | NR | NR | NR |
|  |  | Oral HPV-positive MWH (N=64) | NR | Any hrHPV: 50.0%  HPV16: 4.7%  HPV18: 1.6%  HPV33: 7.8%  HPV35: 3.1%  HPV39: 1.6%  HPV45: 1.6%  HPV51: 1.6%  HPV56: 3.1%  HPV58: 3.1%  HPV59: 1.6%  HPV66: 10.9%  HPV73: 1.6%  HPV85: 6.3% | HPV6: 1.6%  HPV11: 7.8%  HPV13: 3.1%  HPV22: 4.7%  HPV32: 12.5%  HPV61: 7.8%  HPV62: 6.3%  HPV70: 6.3%  HPV71: 1.6%  HPV72: 9.4%  HPV74: 1.6%  HPV81: 3.1%  HPV82: 3.1%  HPV83: 3.1%  HPV84: 6.3%  HPV97: 3.1%  HPV107: 1.6%  HPV145: 1.6% | 9vHPV: 26.6% | Multiple: 28.1%  2 HPV: 25%  Three/four HPV: 3.1% |
| **WWH** | | | | | | | |
| **Perez-Quintanilla *et al.,* 2020^25^**  **Mexico** | February 2014–February 2015 | WWH (N=174) | 92.5% | NR | NR | NR | Single hrHPV: 5.0%  Two hrHPV: 8.0%  Three hrHPV: 9.0%  Four hrHPV: 14.0%  Five hrHPV: 13.0%  Six hrHPV: 18.0%  Seven hrHPV: 14.0%  Eight hrHPV: 9.0%  Single lrHPV: 22.0%  Two lrHPV: 5.0%  Three lrHPV: 1% |
| **Thorsteinsson *et al.,* 2018^34^**  **Denmark** | February 2011–February 2012 | WWH (N=214) | 5.6% | Any hrHPV: 3.7%  HPV16: 0.5%  HPV18: 0.0%  HPV26: 0.0%  HPV31: 0.5%  HPV33: 0.0%  HPV35: 0.5%  HPV39: 0.0%  HPV45: 0.0%  HPV51: 0.5%  HPV52: 1.4%  HPV56: 0.5%  HPV58: 0.5%  HPV59: 0.5%  HPV66: 0.9%  HPV68: 0.0% | Any lrHPV: 3.3%  HPV6: 0.5%  HPV11: 0.5%  HPV40: 0.5%  HPV42: 0.5%  HPV43: 0.0%  HPV44: 0.0%  HPV53: 1.9%  HPV54: 0.0%  HPV61: 0.5%  HPV62: 0.5%  HPV81: 0.0%  HPV70: 0.0%  HPV72: 0.0%  HPV73: 0.0%  HPV82: 0.0%  HPV83: 0.0%  HPV84: 0.0%  HPV85: 0.0%  HPV89: 0.0% | 9vHPV: 62.5% | Multiple: 1.4% |

Abbreviations: HIV: Human immunodeficiency virus; HPV: human papillomavirus; hr: high-risk; lr: low-risk; MSM: men who have sex with men; MWH: men with HIV; PWH: people with HIV; WWH: women with HIV.

### Supplementary Table 15. Summary of findings reported in the literature related to the prevalence of oral HPV among individuals living with or without HIV

| **Publication, Country** | **Date of data collection** | **Sample size** | **Any oral HPV (%)** | **hrHPV (%)** | **lrHPV (%)** | **HPV vaccine types (%)** | **Multiple/single HPV (%)** |
| --- | --- | --- | --- | --- | --- | --- | --- |
| **PWH** | | | | | | | |
| **Riddell *et al.,* 2022^128^**  **US** | 2015–2017 | PWH (N=245) | 23.0% | Any hrHPV: 18.0%  HPV16: 4.0%  HPV18: 5.0%  HPV31: 1.0%  HPV33: 2.0%  HPV35: 3.0%  HPV39: 4.0%  HPV45: 3.0%  HPV51: 2.0%  HPV52: 0.0%  HPV56: 3.0%  HPV58: 3.0%  HPV59: 2.0%  HPV68: 1.0% | HPV6: 4.0%  HPV11: 2.0%  HPV66: 5.0%  HPV73: 2.0%  HPV90: 2.0% | 9vHPV: 15.0%  High-risk 9vHPV: 11.0%  4vHPV: 10.0% | Single: 14.0%  Multiple: 9.0% |
|  |  | MWH (N=155) | 27.0% | Any hrHPV: 23.0%  HPV16: 5.0%  HPV18: 5.0%  HPV31: 1.0%  HPV33: 3.0%  HPV35: 2.0%  HPV39: 3.0%  HPV45: 3.0%  HPV51: 1.0%  HPV52: 0.0%  HPV56: 5.0%  HPV58: 3.0%  HPV59: 2.0%  HPV68: 0.0% | HPV6: 4.0%  HPV11: 3.0%  HPV66: 6.0%  HPV73: 1.0%  HPV90: 1.0% | 9vHPV: 19.0%  High-risk 9vHPV: 15.0%  4vHPV: 12.0% | Single: 17.0%  Multiple: 10.0% |
|  |  | WWH (N=86) | 15.0% | Any hrHPV: 9.0%  HPV16: 0.0%  HPV18: 3.0%  HPV31: 0.0%  HPV33: 1.0%  HPV35: 0.0%  HPV39: 3.0%  HPV45: 2.0%  HPV51: 1.0%  HPV52: 0.0%  HPV56: 0.0%  HPV58: 2.0%  HPV59: 1.0%  HPV68: 2.0% | HPV6: 5.0%  HPV11: 1.0%  HPV66: 2.0%  HPV73: 1.0%  HPV90: 3.0% | 9vHPV: 9.0%  High-risk 9vHPV: 6.0%  4vHPV: 7.0% | Single: 8.0%  Multiple: 7.0% |
|  |  | PWoH (N=198) | 10.0% | Any hrHPV: 7.0%  HPV16: 1.0%  HPV18: 2.0%  HPV31: 0.0%  HPV33: 0.0%  HPV35: 1.0%  HPV39: 1.0%  HPV45: 0.0%  HPV51: 0.0%  HPV52: 1.0%  HPV56: 0.0%  HPV58: 0.0%  HPV59: 0.0%  HPV68: 1.0% | HPV6: 2.0%  HPV11: 0.0%  HPV66: 3.0%  HPV73: 1.0%  HPV90: 0.0% | 9vHPV: 6.0%  High-risk 9vHPV: 4.0%  4vHPV: 5.0% | Single: 9.0%  Multiple: 1.0% |
|  |  | Men without HIV (N=83) | 16.0% | Any hrHPV: 12.0%  HPV16: 2.0%  HPV18: 4.0%  HPV31: 0.0%  HPV33: 0.0%  HPV35: 1.0%  HPV39: 1.0%  HPV45: 0.0%  HPV51: 0.0%  HPV52: 1.0%  HPV56: 0.0%  HPV58: 0.0%  HPV59: 0.0%  HPV68: 0.0% | HPV6: 2.0%  HPV11: 0.0%  HPV66: 5.0%  HPV73: 1.0%  HPV90: 0.0% | 9vHPV: 10.0%  High-risk 9vHPV: 7.0%  4vHPV: 8.0% | Single: 14.0%  Multiple: 1.0% |
|  |  | Women without HIV (N=115) | 5.0% | Any hrHPV: 3.0%  HPV16: 0.0%  HPV18: 1.0%  HPV31: 0.0%  HPV33: 0.0%  HPV35: 0.0%  HPV39: 1.0%  HPV45: 0.0%  HPV51: 0.0%  HPV52: 0.0%  HPV56: 0.0%  HPV58: 0.0%  HPV59: 0.0%  HPV68: 1.0% | HPV6: 2.0%  HPV11: 0.0%  HPV66: 1.0%  HPV73: 1.0%  HPV90: 0.0% | 9vHPV: 3.0%  High-risk 9vHPV: 1.0%  4vHPV: 3.0% | Single: 4.0%  Multiple: 1.0% |
| **MWH** | | | | | | | |
| **Gheit *et al.,* 2020^129^**  **Italy** | November 2014–February 2018 | MSM with HIV (N=117) | 23.9% | HPV16: 2.6%  HPV18: 2.6%  HPV26: 0.0%  HPV33: 0.9%  HPV35: 0.0%  HPV39: 1.7%  HPV45: 0.9%  HPV51: 1.7%  HPV56: 0.0%  HPV58: 0.0%  HPV59: 1.7%  HPV68: 1.7%  HPV73: 0.9% | HPV6: 0.9%  HPV11: 1.7%  HPV53: 0.9%  HPV54: 0.0%  HPV55: 3.4%  HPV61: 1.7%  HPV62: 1.7%  HPV66: 1.7%  HPV69: 0.9%  HPV70: 0.9%  HPV71: 0.9%  HPV72: 3.4%  HPV81: 0.9%  HPV82: 1.7%  HPV83: 0.0%  HPV84: 3.4%  CP6108: 2.6%  IS39: 3.4% | NR | Multiple: 35.7% |
|  |  | MSM without HIV (N=193) | 17.1% | HPV16: 4.1%  HPV18: 0.5%  HPV26: 0.5%  HPV33: 1.0%  HPV35: 0.5%  HPV39: 0.0%  HPV45: 1.5%  HPV51: 0.0%  HPV56: 2.1%  HPV58: 0.5%  HPV59: 0.5%  HPV68: 1.0%  HPV73: 0.5% | HPV6: 0.5%  HPV11: 0.5%  HPV53: 1.5%  HPV54: 0.5%  HPV55: 1.0%  HPV61: 0.0%  HPV62: 0.5%  HPV66: 1.5%  HPV69: 0.0%  HPV70: 0.5%  HPV71: 0.0%  HPV72: 0.5%  HPV81: 0.0%  HPV82: 0.5%  HPV83: 1.0%  HPV84: 1.0%  CP6108: 2.6%  IS39: 0.0% | NR | Multiple: 27.3% |
| **Nemcova *et al.,* 2022^114^**  **Czech Republic** | May 2017–December 2020 | MSM with HIV (N=187) | 24.7% | Any hrHPV: 7.0%  HPV16: 2.7% | Any lrHPV: 5.4% | 9vHPV: 6.5%  High-risk 9vHPV: 5.4% | Multiple: 2.7% |
|  |  | MSM without HIV (N=17) | 11.8% | Any hrHPV: 0.0%  HPV16: 0.0% | Any lrHPV: 11.8% | 9vHPV: 5.9%  High-risk 9vHPV: 0.0% | Multiple: 0.0% |
| **Lin *et al.,* 2018^94^**  **Taiwan** | January 2013–December 2016 | MSM with HIV (N=26) | NR | Any hrHPV: 18.5% | Any lrHPV: 16.7% | NR | NR |
|  |  | MSM without HIV (N=34) | NR | Any hrHPV: 3.8% | Any lrHPV: 38.5% | NR | NR |
| **Oliver *et al.,* 2018^130^**  **US** | July 2012–August 2014 | MSM with HIV (N=88) | 19.3% | NR | NR | 9vHPV: 10.2% | NR |
|  |  | MSM without HIV (N=834) | 8.4% | NR | NR | 9vHPV: 3.4% | NR |
| **Popova *et al.,* 2020^73^**  **Russia** | February 2018–October 2019 | MSM with HIV (N=73) | NR | Any hrHPV: 13.7% | NR | NR | NR |
|  |  | MSM without HIV (N=66) | NR | Any hrHPV: 6.1% | NR | NR | NR |
|  |  | MSW with HIV (N=58) | NR | Any hrHPV: 5.2% | NR | NR | NR |
|  |  | MSW without HIV (N=59) | NR | Any hrHPV: 1.7% | NR | NR | NR |
| **Rollo *et al.,* 2021^131^**  **Italy** | NR | MSM with HIV (N=85) | 25.9% | Any hrHPV: 11.8%  HPV16: 2.4% | NR | NR | NR |
|  |  | MSM without HIV (N=76) | 19.7% | Any hrHPV: 11.8%  HPV16: 9.2% | NR | NR | NR |
| **Tsikis *et al.,* 2018^132^**  **Greece** | July 2015–October 2015 | MWH (N=58) | 8.6% | NR | NR | NR | NR |
|  |  | Men without HIV (N=216) | 2.3% | NR | NR | NR | NR |
| **Ucciferri *et al.,* 2018^117^**  **Italy** | NR | MSM with HIV (N=45) | 29.6% | NR | NR | NR | NR |
|  |  | MSM without HIV (N=45) | 29.4% | NR | NR | NR | NR |
| **WWH** | | | | | | | |
| **Suehiro *et al.,* 2020^57^**  **Brazil** | September 2017–May 2018 | WWH (N=115) | 14.8% | Any hrHPV: 6.1% | Any lrHPV: 7.8% | NR | Multiple: 4.4% |
|  |  | HPV positive WWH (N=17) | NR | HPV18: 16.6%  HPV39: 33.3%  HPV45: 16.6%  HPV52: 16.6%  HPV68: 16.6% | HPV6: 28.6%  HPV62: 21.4%  HPV81: 21.4% | NR | NR |
|  |  | Women without HIV (N=139) | 9.4% | Any hrHPV: 3.6%  HPV51: 11.9%  HPV68: 11.9% | Any lrHPV: 3.6%  HPV6: 30.0%  HPV43: 20.0% | NR | Multiple: 2.9% |

Abbreviations: HIV: Human immunodeficiency virus; HPV: human papillomavirus; hr: high-risk; lr: low-risk; MSM: men who have sex with men; MWH: men with HIV; PWH: people with HIV; PWoH: people without HIV; WWH: women with HIV.

### Supplementary Table 16. Summary of findings reported in the literature related to the incidence of oral HPV

| **Publication, Country** | **Date of data collection** | **Median time to follow-up from baseline** | **Population and sample size** | **Incidence** |
| --- | --- | --- | --- | --- |
| **Castillejos-Garcia *et al.,* 2018^126^**  **Mexico** | September 2013–February 2015 | 91 days (Q1–Q3: 91–105 days) | PWH (N=97) | Three-month incident HPV: 33.9%  Three-month incident hrHPV: 22.0% |
| **Giuliani *et al.,* 2020^133^**  **Italy** | November 2014–February 2018 | 13 months (IQR: 10–19 months) | MSM with HIV (N=103) | Any HPV: 21.2 per 1,000 person-months  Any hrHPV: 15.0 per 1,000 person-months  HPV16: 0.8 per 1,000 person-months  HPV33: 1.5 per 1,000 person-months  HPV39: 0.7 per 1,000 person-months  HPV51: not estimable  HPV56: not estimable  HPV58: not estimable  HPV59: 0.8 per 1,000 person-months  HPV66: not estimable  HPV68: not estimable |
|  |  |  | MSM without HIV (N=141) | Any HPV: 15.0 per 1,000 person-months  Any hrHPV: 6.5 per 1,000 person-months  HPV16: 3.2 per 1,000 person-months  HPV33: 0.6 per 1,000 person-months  HPV39: 1.7 per 1,000 person-months  HPV51: not estimable  HPV56: 1.1 per 1,000 person-months  HPV58: not estimable  HPV59: not estimable  HPV66: 1.4 per 1,000 person-months  HPV68: 0.8 per 1,000 person-months |

Abbreviations: AIDS: Acquired immunodeficiency syndrome; HIV: human immunodeficiency virus; HPV: human papillomavirus; hr: high-risk; IQR: interquartile range; lr: low-risk; MSM: men who have sex with men; MWH: men with HIV; PWH: people with HIV; Q1: first quartile; Q3: third quartile; WWH: women with HIV.

### Supplementary Table 17. Summary of studies reported in the literature related to prevalence of concurrent HPV infection

| **Publication, Country** | **Date of data collection** | **Sample size** | **Infection type** | **Any HPV (%)** |
| --- | --- | --- | --- | --- |
| **Concurrent anal and oral HPV infection** | | | | |
| **Ablanedo *et al.,* 2018^77^**  **Mexico** | January 2014–January 2016 | MWH (N=107) | Concurrent anal and oral HPV infection | 7.5% |
|  |  |  | Concordant anal and oral HPV infection | 5.6% |
| **Callejo *et al.,* 2022^107^**  **Spain** | May 2019–May 2021 | MSM with HIV (N=103) | Concordant anal and oral HPV infection | 26.2% |
|  |  | MSM with HIV with oral HPV infection (N=14) | Concurrent anal HPV infection | 100% |
|  |  | MSM with HIV with oral HPV infection (N=14) | Concordant anal HPV infection | 28.6% |
| **Hidalgo-Tenorio *et al.,* 2023^66^**  **Spain** | NR | PWH (N=300) | Concurrent anal and oropharyngeal HPV infection | 8.9% |
| **Méndez-Martínez et al., 2020^101^**  **Mexico** | NR | Anal HPV16-positive MSM with HIV (N=102) | Concurrent oral HPV infection | Any lrHPV: 66.7%  HPV6: 34.3%  HPV11: 53.9%  HPV16: 80.4%  HPV18: 52.9%  HPV52: 49.0% |
|  |  |  | Concordant anal and oral HPV infection | HPV6: 14.7%  HPV11: 37.3%  HPV16: 80.4%  HPV18: 27.5%  HPV52: 26.5% |
| **Parisi *et al.,* 2019^115^**  **Italy** | January 2013–December 2013 | MSM with HIV with anal hrHPV infection (N=77) | Oral HPV infection | hrHPV: 9.1%  non-hrHPV: 19.5% |
| **Rollo *et al.,* 2021^131^**  **Italy** | NR | MSM with HIV (N=85) | Concurrent anal and oral HPV infection | 25.9% |
|  |  |  | Concordant anal and oral HPV infection | 11.8% |
|  |  | MSM without HIV (N=76) | Concurrent anal and oral HPV infection | 17.1% |
|  |  |  | Concordant anal and oral HPV infection | 6.6% |
| **Vergori *et al.,* 2018^81^**  **Italy** | February 2015–June 2016 | HPV-positive MSM with HIV (N=265) | Concurrent anal and oral HPV infection | 22.3% |
|  |  | MSM with HIV with concurrent infection (N=59) | Concordant anal and oral HPV infection | 13.6% |
|  |  | MSM with HIV with anal HPV infection (N=260) | Concurrent oral HPV infection | 22.7% |
| **Concurrent anal and cervical HPV infection** | | | | |
| **Al Shaarani *et al.,* 2020^36^**  **US** | July 2012–July 2015 | WWH (N=335) | Concurrent anal and cervical HPV infection | 27.8% |
|  |  | WWH with concurrent anal and cervical HPV infection (N=93) | Concordant anal and cervical HPV infection | 46% |
| **Gupta *et al.,* 2022^122^**  **India** | October 2019–October 2021 | WWH (N=141) | Concurrent anal and cervical HPV infection | Any hrHPV: 16.5% |
|  |  | Women without HIV (N=161) | Concurrent anal and cervical HPV infection | Any hrHPV: 3.1% |
| **Liu *et al.,* 2023^39^**  **US** | 2012–2019 | WWH (N=381) | Concurrent anal and cervical HPV infection | Any hrHPV: 26.0%  HPV16: 2.0%  HPV16/18: 6.0% |
| **Concurrent cervical and oral HPV infection** | | | | |
| **Perez-Quintanilla *et al.,* 2020^25^**  **Mexico** | February 2014–February 2015 | WWH (N=174) | Concurrent cervical and oral HPV infection | 89.1% |
| **Other concurrent HPV infections** | | | | |
| **Ucciferri *et al.,* 2018^117^**  **Italy** | NR | HPV-positive MSM with HIV (N=27) | Multisite HPV infection (anal, oral, and genital) | 56.0% |
|  |  |  | Concurrent HPV infection across all sites | 3.7% |
|  |  | HPV-positive MSM without HIV (N=17) | Multisite HPV infection (anal, oral, and genital) | 65.0% |
|  |  |  | Concurrent HPV infection across all sites | NR |

Abbreviations: 9v: Nine-valent; HIV: human immunodeficiency virus; HPV: human papillomavirus; hr: high-risk; MSM: men who have sex with men; MWH: men with HIV; NR: not reported; PWH: people with HIV; WWH: women with HIV.

### Supplementary Table 18. Summary of findings reported in the literature related to the prevalence of any cervical abnormality or lesion in WWH compared to women without HIV

| **Publication, Country** | **Date of data collection** | **Population** | **Outcome** |
| --- | --- | --- | --- |
| **North America** | | | |
| **Castle *et al.*, 2022^59^**  **US** | January 2003–February 2021 | WWH (N=608) and women without HIV (N=67,488)  Mean age: 44.56 years (range: 30–64 years) | **Prevalence of any abnormality:**   - With HIV: 84 (13.8%) - Without HIV: 2,615 (3.9%) - p<0.001   **Prevalence of low-grade lesions:**   - With HIV: 75 (12.3%) - Without HIV: 2,068 (3.1%) |
| **Klein *et al.*, 2020^60^**  **US** | January 2003–April 2019 | WWH (N=70) and matched HIV-controls (N=70)  Median age:   - WWH: 66 years (IQR: 65–68) - Without HIV: 66 years (IQR: 65–68) | **Prevalence of any abnormality:**   - WWH: NR (31.0%) - Without HIV: NR (10.0%) - p<0.0001   **Prevalence of ASC-US:**   - With HIV: NR (14.0%) - Without HIV: NR (7.0%)   **Prevalence of LSIL:**   - With HIV: NR (11.0%) - Without HIV: NR (7.0%)   **Prevalence of HSIL:**   - With HIV: NR (2.0%) - Without HIV: NR (0.0%)   **Prevalence of ASC-H:**   - With HIV: NR (<1.0%) - Without HIV: NR (0%) |
| **Europe** | | | |
| **Bouassa *et al.,* 2019^134^**  **France** | 2018 | WWH (N=37) and women without HIV (N=13)  Mean age: 41.7 years (range: 25–65 years) | **Prevalence of ASC-US:**   - With HIV: 2 (5.4%) - Without HIV: 1 (7.7%)   **Prevalence of low-grade lesions:**   - With HIV: 3 (8.1%) - Without HIV: 0 (0.0%)   **Prevalence of high-grade lesions:**   - With HIV: 2 (5.4%) - Without HIV: 0 (0%) |
| **Cavallari *et al.*, 2023^65^**  **Italy** | NR | WWH (N=26) and women without HIV (N=74)  Median age: 45 years (range: NR) | **Prevalence of LSIL:**   - With HIV: NR (40.0%) - Without HIV: NR (35.0%) |
| **Central and South America** | | | |
| **Camargo *et al.,* 2018^135^**  **Colombia** | February 2007–February 2013 | WWH (N=240) and women without HIV (N=1,114) Mean age:   - WWH: 37.3 years - Without HIV: 37.5 years | **Prevalence of any abnormality:**   - With HIV: 70 (29.1%; 95% CI: 23.4–35.3%) - Without HIV: 227 (20.4%; 95% CI: 18.0–22.8%)   **Prevalence of ASC-US:**   - With HIV: 26 (10.8%) - Without HIV: 121 (10.8%)   **Prevalence of HSIL:**   - With HIV: 4 (1.6%) - Without HIV: 13 (1.2%)   **Prevalence of LSIL:**   - With HIV: 40 (16.7%) - Without HIV: 93 (8.4%) |
| **Suehiro *et al.*, 2020^57^**  **Brazil** | September 2017–May 2018 | WWH (N=115) and controls without HIV (N=139)  Median age:   - WWH: 42.17 (SD: 10.18) - Without HIV: 41.4 (SD: 12.31) | **Prevalence of any abnormality:**   - WWH: NR (13.0%) - Without HIV: NR (5.0%) - p=0.04   **Prevalence of ASC-US:**   - WWH: NR (2.6%) - Without HIV: NR (1.4%) - p=0.66   **Prevalence of LSIL:**   - WWH: NR (7.8%) - Without HIV: NR (2.8%) - p=0.15   **Prevalence of HSIL:**   - WWH: NR (2.6%) - Without HIV: NR (0.8%) - p=0.33 |
| **Asia** | | | |
| **Gupta *et al.,* 2022^136^**  **India** | October 2019–October 2021 | WWH (N=141) and women without HIV (N=161)  Mean age: NR (range: NR)  [135 WWH and 160 women without HIV included within analysis] | **Prevalence of any abnormality:**   - With HIV: 19 (14.1%) - Without HIV: 5 (3.1%) - p<0.001 |
| **Jain *et al.*, 2021^55^**  **India** | NR | WWH (N=50) and controls without HIV (N=50)  Mean age: 32.7 years (range: 22–52) | **Prevalence of ASC-US:**   - With HIV: 3 (6.0%) - Without HIV: 1 (2.0%)   **Prevalence of LSIL:**   - With HIV: 8 (16.0%) - Without HIV: 6 (12.0%)   **Prevalence of HSIL:**   - With HIV: 2 (4.0%) - Without HIV: 5 (10.0%) |
| **Peyyala *et al.,* 2019^56^**  **India** | December 2016–October 2018 | WWH (N=110) and controls without HIV (N=110)  Mean age: NR (range: 30–59 years) | **Prevalence of any abnormality:**   - WWH: NR (5.4%) - Without HIV: NR (1.8%) |
| **Africa** | | | |
| **Hopkins *et al.*, 2021^42^**  **South Africa** | June 2018–March 2019 | WWH (N=57) and women without HIV (N=223)  Median age: 31 years (IQR: 25–40 years) | **Prevalence of ASC-US:**   - With HIV: 6 (10.5%) - Without HIV: 9 (4.0%) - p=0.05   **Prevalence of LSIL:**   - With HIV: 8 (14.0%) - Without HIV: 15 (6.7%) - p=0.07   **Prevalence of HSIL:**   - With HIV: 6 (10.5%) - Without HIV: 7 (3.1%) - p=0.01 |
| **Karani *et al.,* 2020^44^**  **Kenya** | July 2018–July 2019 | HPV-positive WWH (N=42) and HPV-positive controls without HIV (N=42)  Mean age:   - WWH: 40.36 years (SD: 11.318) - Without HIV: 35.21 years (SD: 9.495) | **Prevalence of ASC-US:**   - With HIV: 6 (7.14%) - Without HIV: 10 (11.90%) - p=0.09   **Prevalence of LSIL:**   - With HIV: 9 (10.71%) - Without HIV: 23 (27.38%) - p=0.002   **Prevalence of HSIL:**   - With HIV: 23 (27.38%) - Without HIV: 7 (8.33%) - p=0.006 |
| **Kinotia *et al.*, 2022^45^**  **Kenya** | 2019 | WWH (N=106) and women without HIV (N=211)  Mean age: 34.3 years (range: 18–46) | **Prevalence of any abnormality:**   - WWH: 65 (20.5%) - Without HIV: 13 (4.1%) - p=0.001 |
| **Lewis *et al.*, 2022^137^**  **Malawi** | June 2017–November 2019 | WWH (N=1,305) and women without HIV (N=100) receiving first time VIA screening  Mean age: NR (range: NR) | **Prevalence of abnormal result upon VIA screening:**   - WWH: 78 (6.0%) - Without HIV: 3 (3.0%)   **Prevalence of precancerous lesions upon VIA screening:**   - WWH: 65 (5.0%) - Without HIV: 3 (3.0%) |
| **Mbuya *et al.,* 2020^47^**  **Tanzania** | 2013–2017 | WWH (N=215) and women without HIV (N=253)  Median age: 38 years (IQR: 31–45)  [200 WWH and 240 women without HIV included within analysis] | **Prevalence of LSIL:**   - With HIV: 10 (5.0%) - Without HIV: 5 (2.0%)   **Prevalence of HSIL:**   - With HIV: 7 (4.0%) - Without HIV: 6 (3.0%) |
| **Nakisige *et al.*, 2022^138^**  **Uganda** | July 2017–February 2020 | WWH (N=188) and women without HIV (N=116) with cervical dysplasia detected using visual inspection with acetic acid (VIA)  Median age:   - WWH: 34 years (IQR: 28–40) - Without HIV: 33 years (IQR: 25–43) | **Prevalence of CIN1:**   - With HIV: 65 (35%) - Without HIV: 34 (29.0%)   **Prevalence of CIN2:**   - With HIV: 14 (7.0%) - Without HIV: 4 (3.0%)   **Prevalence of CIN3:**   - With HIV: 41 (22.0%) - Without HIV: 7 (6.0%) |
| **Ndizeye *et al.*, 2019^139^**  **Burundi** | May 2013–July 2013 in Kirundo, March 2016–May 2016 in Bujumbura | Urban WWH (N=151) and women without HIV (N=149)  Mean age:   - WWH: 41.1 years (SD: 9.7) - Without HIV: 39.7 years (SD: 8.7) | **Prevalence of ASC-H:**   - With HIV: 2 (1.3%) - Without HIV: 0 (0.0%)   **Prevalence of ASC-US:**   - With HIV: 13 (8.6%) - Without HIV: 2 (1.3%)   **Prevalence of HSIL:**   - With HIV: 3 (2.0%) - Without HIV: 2 (1.3%)   **Prevalence of LSIL:**   - With HIV: 16 (10.6%) - Without HIV: 3 (2.0%) |
| **Ogu *et al.*, 2019^140^**  **Nigeria** | July 2018–January 2019 | WWH on HAART (N=105) and women without HIV (N=104)  Mean age:   - WWH: 42 years (SD: 4) - Without HIV: 41.7 (SD: 5.5) | **Prevalence of ASC-US:**   - With HIV: 15 (14.3%) - Without HIV: 5 (4.8%)   **Prevalence of HSIL:**   - With HIV: 1 (1.0%) - Without HIV: 0 (0.0%)   **Prevalence of LSIL:**   - With HIV: 4 (3.8%) - Without HIV: 2 (1.9%) |
| **Ogunsowo *et al.*, 2022^141^**  **Nigeria** | NR | WWH (N=165) and women without HIV (N=165)  Mean age: 36.8 years (SD: 5.6) | **Prevalence of ASC-US:**   - With HIV: 12 (7.3%) - Without HIV: 7 (4.2%)   **Prevalence of HSIL:**   - With HIV: 8 (4.8%) - Without HIV: 2 (1.2%)   **Prevalence of LSIL:**   - With HIV: 21 (12.7%) - Without HIV: 3 (1.8%) |
| **Okoye *et al.,* 2020^50^**  **Nigeria** | April 2017–June 2018 | WWH (N=105) and women without HIV (N=105)  Mean age:   - WWH: 41.55 years (SD: 11.71) - Without HIV: 39.45 (SD: 11.16) | **Prevalence of ASC-US:**   - With HIV: 20 (19.9%) - Without HIV: 12 (11.4%)   **Prevalence of HSIL:**   - With HIV: 14 (13.3%) - Without HIV: 6 (5.7%)   **Prevalence of LSIL:**   - With HIV: 20 (19.9%) - Without HIV: 8 (7.6%) |
| **Swai *et al.,* 2020^52^**  **Tanzania** | August 2015–October 2017 | WWH (N=604) and women without HIV (N=2,786) aged 25–60 years  Mean age: 40.7 years (SD: 8.7) | **Prevalence of high-grade lesions:**   - With HIV: 60 (9.9%) - Without HIV: 63 (2.3%) |
| **Sweet *et al.*, 2020^53^**  **Kenya** | August 2009–March 2011 | FSW with (N=84) and without (N=264) HIV  Mean age: 28 years (range: 18–48) | **Prevalence of ASC-US/AG-US:**   - With HIV: 4 (4.8%) - Without HIV: 10 (3.8%) - p=0.75   **Prevalence of LSIL:**   - With HIV: 16 (19.1%) - Without HIV: 21 (8.0%) - p<0.01   **Prevalence of HSIL/SCC:**   - With HIV: 11 (13.1%) - Without HIV: 4 (1.5%) - p<0.01   **Prevalence of CIN2+:**   - With HIV: 14 (16.7%) - Without HIV: 5 (1.9%) - p<0.01 |

Abbreviations: AG-US: Atypical glandular cells of undetermined significance; ASC-H: atypical squamous cells not excluding high-grade; ASC-US: atypical squamous cells of undetermined significance; CI: confidence interval; CIN: cervical intraepithelial neoplasia; HAART: highly active antiretroviral therapy; HIV: human immunodeficiency virus; HPV: human papillomavirus; hrHPV: high-risk human papillomavirus; HSIL: high-grade squamous intraepithelial lesions; IQR: interquartile range; LSIL: low-grade squamous intraepithelial lesions; NR: not reported; SCC: squamous cell carcinoma; SD: standard deviation; US: United States; VIA: visual inspection with acetic acid; WWH: women with HIV.

### Supplementary Table 19. Summary of findings reported in the literature related to the prevalence or incidence of any cervical abnormality or lesion in WWH

| **Publication, country** | **Date of data collection** | **Population** | **Outcome** |
| --- | --- | --- | --- |
| **North America** | | | |
| **Massad *et al.*, 2020^142^**  **US** | 1994–1995; 2001–2002; 2011–2012; 2013–2015 | WWH (N=3133 [1994–1994: 1769; 2001–2002: 684; 2011–2012: 231; 2013–2015: 449])  Mean age: 36.9 years (SD: 8.3) | **Prevalence of ASC-US:**   - 1994–1995 cohort: 368 (21.0%) - 2001–2002 cohort: 118 (17.0%) - 2011–2012 cohort: 28 (12.0%) - 2013–2015 cohort: 33 (7.0%)   **Prevalence of LSIL:**   - 1994–1995 cohort: 268 (15.0%) - 2001–2002 cohort: 65 (10.0%) - 2011–2012 cohort: 16 (7.0%) - 2013–2015 cohort: 26 (6.0%)   **Prevalence of HSIL+:**   - 1994–1995 cohort: 43 (2.0%) - 2001–2002 cohort: 12 (2.0%) - 2011–2012 cohort: 2 (1.0%) - 2013–2015 cohort: 12 (3.0%) |
| **Ye *et al.*, 2020^143^**  **US** | January 2006–March 2018 | WWH ≥18 years (N=1,038)  Mean age: 41.8 years (SD: 10.6) | **Incidence of cervical LSIL:** 232.6 (95% CI: 197.7–267.4) per 10,000 person-years  **Incidence of cervical HSIL:** 108 (95% CI: 84.5–131.7) per 10,000 person-years |
| **Europe** | | | |
| **Carriero *et al.,* 2018^30^**  **Italy** | 2009–2014 | Pregnant WWH (N=48)  Mean age: 31 years (SD: 9) | **Prevalence of ASC-US:** 2 (4.0%)  **Prevalence of any SIL:** 11 (23.0%)  **Prevalence of LSIL:** NR (18.7%)  **Prevalence of HSIL:** NR (4.2%) |
| **Floridia *et al.,* 2022^144^**  **Italy** | April 2021 | Vaccinated pregnant WWH (N=8)  Median age: 36 years (IQR: 27.5–39.5 years) | **Prevalence of normal cytology:** 5 (83.4%)  **Prevalence of LSIL: 1 (16.7%)** |
|  |  | Non-vaccinated pregnant WWH (N=725)  Median age: 33 years (IQR: 28–37 years) | **Prevalence of normal cytology:** 459 (79.4%)  **Prevalence of ASC-US:** 10 (1.7%)  **Prevalence of atypical glandular cells:** 1 (0.2%)  **Prevalence of LSIL:** 64 (11.1%)  **Prevalence of HSIL:** 44 (7.6%) |
| **Fusco *et al.,* 2018^145^**  **Italy** | 2006–2016 | WWH (N=321)  Mean age: 41 years (range: 18–76 years)  [Cytology from 161 HPV-positive WWH] | **Prevalence of ASC-US:** 36 (22.0%)  **Prevalence of LSIL:** 21 (13.0%)  **Prevalence of HSIL:** 4 (2.0%) |
| **Hidalgo-Tenorio *et al.,* 2023^66^**  **Spain** | NR | PWLH (N=300)  Mean age: 45.1 years (SD: 11)  [64 WWH included] | **Prevalence of ASC-US:** 4 (1.3%)  **Prevalence of LSIL:** 3 (1.0%)  **Prevalence of CIN1:** 1 (0.7%) |
| **Menon *et al.*, 2018^146^**  **Belgium** | NR | WWH (N=593)  Median age: 30 years (IQR: 26–38 years) | **Prevalence of ASC-H:** 1 (0.2%)  **Prevalence of ASC-US:** 41 (6.9%)  **Prevalence of LSIL:** 90 (15.2%)  **Prevalence of HSIL:** 11 (1.9%) |
| **Videla *et al.,* 2019^63^**  **Spain** | Baseline visit: March 1999–January 2003  Follow-up: annually to 17 years (median: 13.2 years) | WWH participating in an annual PAP smear screening program (N=67)  Mean age: 36 years (SD: 6.5) | **Prevalence of baseline ASC-US:** 6 (9.0%)  **Prevalence of baseline LSIL:** 7 (10.0%)  **Prevalence of baseline HSIL:** 4 (6.0%)  **Prevalence of HSIL at follow-up:** 9 (13.0%) |
| **Central and South America** | | | |
| **Cambou *et al.*, 2015^147^**  **Brazil** | 2006–2012 with annual follow-ups (not all participants returned annually) | Non-vaccinated WWH aged ≥18 years living in urban areas; data collection in 2006 (N=102)  Mean age: NR (range: NR) | **Prevalence of ASC-H:** 0 (0.0%)  **Prevalence of ASC-US:** 10 (10.0%)  **Prevalence of atypical glandular cells:** 0 (0.0%)  **Prevalence of LSIL:** 15 (15.0%)  **Prevalence of HSIL:** 0 (0.0%) |
|  |  | Non-vaccinated WWH aged ≥18 years living in urban areas; data collection in 2007 (N=167)  Mean age: NR (range: NR) | **Prevalence of ASC-H:** 0 (0.0%)  **Prevalence of ASC-US:** 12 (7.0%)  **Prevalence of atypical glandular cells:** 1 (<1.0%)  **Prevalence of LSIL:** 18 (11.0%)  **Prevalence of HSIL**: 4 (2.0%) |
|  |  | Non-vaccinated WLWLH aged ≥18 years living in urban areas; data collection in 2008 (N=240)  Mean age: NR (range: NR) | **Prevalence of ASC-H:** 0 (0.0%)  **Prevalence of ASC-US:** 26 (11.0%)  **Prevalence of atypical glandular cells:** 0 (0.0%)  **Prevalence of LSIL:** 21 (9.0%)  **Prevalence of HSIL:** 2 (<1.0%) |
|  |  | Non-vaccinated WWH aged ≥18 years living in urban areas; data collection in 2009 (N=282)  Mean age: NR (range: NR) | **Prevalence of ASC-H:** 0 (0.0%)  **Prevalence of ASC-US:** 29 (10.0%)  **Prevalence of atypical glandular cells:** 0 (0.0%)  **Prevalence of LSIL:** 42 (15.0%)  **Prevalence of HSIL:** 8 (3.0%) |
|  |  | Non-vaccinated WWH aged ≥18 years living in urban areas; data collection in 2010 (N=256)  Mean age: NR (range: NR) | **Prevalence of ASC-H:** 0 (0.0%)  **Prevalence of ASC-US:** 24 (9.0%)  **Prevalence of atypical glandular cells:** 1 (<1.0%)  **Prevalence of LSIL:** 34 (13.0%)  **Prevalence of HSIL:** 2 (1.0%) |
|  |  | Non-vaccinated WWH aged ≥18 years living in urban areas; data collection in 2011 (N=260)  Mean age: NR (range: NR) | **Prevalence of ASC-H:** 0 (0.0%)  **Prevalence of ASC-US:** 28 (10.0%)  **Prevalence of atypical glandular cells:** 2 (1.0%)  **Prevalence of LSIL:** 33 (13.0%)  **Prevalence of HSIL:** 7 (3.0%) |
|  |  | Non-vaccinated WWH aged ≥18 years living in urban areas; data collection in 2012 (N=225)  Mean age: NR (range: NR) | **Prevalence of ASC-H:** 0 (0.0%)  **Prevalence of ASC-US:** 21 (9.0%)  **Prevalence of atypical glandular cells:** 0 (0.0%)  **Prevalence of LSIL:** 23 (10.0%)  **Prevalence of HSIL:** 1 (<1.0%) |
| **Badial *et al.*, 2018^21^**  **Brazil** | 2010–2012 | WWH (N=40)  Mean age: NR (range: NR) | **Prevalence of lesions:**   - 2010–2011: 9 (22.5%) - 2011–2012: 11 (27.5%) |
| **Ortiz-Gutierrez *et al.,* 2021^24^**  **Mexico** | NR | WWH (N=40)  Mean age: NR (range: NR) | **Prevalence of CIN1+:** 9 (22.5%) |
| **Teixeira *et al.*, 2018^26^**  **Brazil** | May 2014–February 2015 | WWH (N=325)  Median age: 40.7 (IQR: 33.1–46.2 years) | **Prevalence of ASC-US:** 9 (2.7%)  **Prevalence of ASC-H:** 5 (1.7%)  **Prevalence of LSIL:** 25 (8.1%)  **Prevalence of HSIL:** 10 (3.4%) |
| **Tosato Boldrini *et al.*, 2021^27^**  **Brazil** | February 2014–October 2015 | WWH that underwent cervical cytological testing (N=151)  Median age: 41 (SD: 10.8) | **Prevalence of ASC-US+:** NR (6.7%) |
|  |  | WWH that underwent cervical biopsy (N=28) | **Prevalence of CIN:** 28 (100% of sample that underwent biopsy or 18.5% of total sample)  **Prevalence of LSIL:** 10 (35.7% of sample that underwent biopsy or 6.6% of total sample)  **Prevalence of HSIL:** 18 (64.2% of sample that underwent biopsy or 11.9% of total sample) |
| **Asia** | | | |
| **Gupta *et al.,* 2022^136^**  **India** | October 2019–October 2021 | WWH (N=135)  Mean age: NR (range: NR) | **Prevalence of ASC-US:** 7 (5.2%)  **Prevalence of ASC-H:** 1 (0.9%)  **Prevalence of LSIL:** 8 (5.9%)  **Prevalence of HSIL:** 3 (2.2%)  **Prevalence of CIN2+:** NR (4.4%) |
| **Joshi *et al.,* 2022^148^**  **India** | 2010–2011 | WWH (N=1141)  Mean age: NR (range: NR) | **Prevalence of CIN at baseline:** 101 (8.9%) |
| **Pahwa *et al.,* 2022^16^**  **India** | May 2010–June 2015 | WWH (N=291)  Mean age: NR (range: NR) | **Prevalence of CIN1:** NR (6.2%; 95% CI: 3.7–9.6%)  **Prevalence of CIN2+:** NR (8.6%; 95% CI: 5.6–12.4%) |
| **Prathima *et al.,* 2021^149^**  **India** | September 2016–September 2017 | WWH aged 21–65 years (N=100)  Mean age: NR (range: 21–65 years) | **Prevalence of an inflammatory smear:** 48 (48.0%)  **Prevalence of abnormal cervical cytology:** 30 (30.0%)  **Prevalence of ASC-US:** 5 (5.0%)  **Prevalence of LSIL:** 15 (15.0%)  **Prevalence of HSIL:** 10 (10.0%) |
|  |  | WWH with LSIL (N=15) | **Prevalence of CIN1:** 7 (46.6%)  **Prevalence of CIN2:** 3 (20.0%)  **Prevalence of CIN3:** 0 (0.0%) |
|  |  | WWH with HSIL (N=10) | **Prevalence of CIN1:** 0 (0.0%)  **Prevalence of CIN2:** 5 (50.0%)  **Prevalence of CIN3:** 5 (50.0%) |
| **Qiao *et al.*, 2020^17^**  **China** | May 2015–August 2015 | WWH aged 18–49 years who underwent cervical screening (N=695)  Mean age: NR (range: 18–49 years)  [617 WWH included in cervical screening] | **Prevalence of LSIL:** 18 (2.9%)  **Prevalence of HSIL:** 25 (4.1%) |
| **Shin *et al.*, 2019^150^**  **India** | February 2018 –March 2018 | WWH receiving ART (N=598)  Median age: 37 years (IQR: 32–42 years) | **Prevalence of abnormal cytology:** 77 (12.9%)  **Prevalence of ASC-US:** NR (0.8%)  **Prevalence of LSIL:** NR (6.5%)  **Prevalence of HSIL:** NR (4.9%) |
| **Vasnik *et al.*, 2019^151^**  **India** | NR | WWH with CD4 counts >200 cells/mm^3^ (N=71)  Mean age: 33.34 years (range: 20–55 years) | **Prevalence of ASC-US:** 2 (NR)  **Prevalence of HSIL:** 1 (NR)  **Prevalence of LSIL:** 5 (NR) |
|  |  | WWH with CD4 counts <200 cells/mm^3^ (N=26)  Mean age: 33.34 years (range: 20–55 years) | **Prevalence of ASC-US:** 0 (NR)  **Prevalence of HSIL:** 2 (NR)  **Prevalence of LSIL:** 11 (NR) |
| **Wang *et al.*, 2019^19^**  **China** | March 2014–August 2014 | WWH (N=183) registered in maternal and child health centres of Hezhou county  Median age: 33 years (IQR: 29–37 years)  [80 WWH with detectable HPV included in screening] | **Prevalence of atypical cervical cytology:** NR (53.75%)  **Prevalence of CIN1 (including flat condyloma):** NR (12.50%)  **Prevalence of CIN2:** NR (6.25%)  **Prevalence of CIN3:** NR (5.00%) |
| **Africa** | | | |
| **Guthrie *et al.*, 2020^4^**  **Kenya** | September 2007–December 2009 | WWH in HIV-discordant couples (N=283)  Median age: 28 years (range: 18–50 years)  [268 WWH with cervical cytology results] | **Prevalence of ASC-US/LSIL:** 51 (19.0%)  **Prevalence of ASC-H/HSIL:** 16 (6.0%) |
| **Kremer *et al.*, 2018^5^**  **South Africa** | February 2013–March 2016 | WWH (N=463)  Mean age: NR (range: NR) | **Prevalence of CIN1:** 112 (24.2%)  **Prevalence of CIN2:** 54 (11.7%)  **Prevalence of CIN3:** 113 (24.4%) |
| **Ouladlahsen *et al.*, 2018^152^**  **Morocco** | February 2013–September 2016 | WWH (N=251)  Median age: 39 years (IQR: 32–47 years) | **Prevalence of ASC-US/LSIL/HSIL:** 34 (13.8%)  **Prevalence of LSIL:** NR (7.7%)  **Prevalence of non-dysplastic inflammation:** 125 (50.8%) |
| **Rais *et al.*, 2023^10^**  **Algeria** | May 2018–September 2018 | WWH taking ART (N=100)  Median age: 43 years (range: 24-76 years) | **Prevalence of abnormal cytology**: 66 (66.0%)  **Prevalence of ASC-US:** 10 (10.0%)  **Prevalence of LSIL:** 5 (5.0%)  **Prevalence of HSIL:** 13 (13.0%) |
|  |  | WWH with abnormal pap smears (N=66) | **Prevalence of inflammatory cervical cytology:** 56 (85.0%)  **Prevalence of ASC-US:** 10 (15.0%)  **Prevalence of LSIL:** 13 (19.7%)  **Prevalence of HSIL:** 5 (7.6%) |
| **Yakub *et al.,* 2019^13^**  **Nigeria** | August 2016–May 2017 | WWH attending an antiretroviral clinic with CD4 cell counts (N=193)  Mean age: NR (range: NR) | **Prevalence of unsatisfactory cervical cytology:** 19 (NR)  **Prevalence of cervical cytological inflammation:** 121 (NR)  **Prevalence of premalignant/malignant lesions:** 53 (NR) |
| **NR** | | | |
| **Smeltzer *et al.,* 2018^153^**  **NR** | January 2014–January 2015 | WWH (N=238)  Mean age: NR (range: NR) | **Prevalence of ASC-US/HPV-positivity:** 12 (5.0%)  **Prevalence of LSIL:** 55 (23.1%)  **Prevalence of HSIL:** 7 (2.9%) |

Abbreviations: AG-US: Atypical glandular cells of undetermined significance; ASC-US: atypical squamous cells of undetermined significance; ART: antiretroviral therapy; CI: confidence interval; CIN: cervical intraepithelial neoplasia; FSW: female sex workers; HIV: human immunodeficiency virus; HSIL: high-grade intraepithelial lesions; IQR: interquartile range; LSIL: low-grade squamous intraepithelial lesions; NR: not reported; SCC: squamous cell carcinoma; US: United States; WWH: women with HIV.

### Supplementary Table 20. Comparative and non-comparative studies reporting on the prevalence and incidence of cervical cancer in WWH and women without HIV

| **Publication, Country** | **Date of data collection** | **Population** | **Outcome** |
| --- | --- | --- | --- |
| **Comparative** | | | |
| **Clark *et al.,* 2020^154^**  **US** | October 1999–December 2016 | Veteran WWH (N=1,454) and veteran women without HIV (N=5,816)  Mean age: NR (range: NR) | **Prevalence of cervical cancer:**   - WWH: 22 (NR) - Without HIV: 24 (NR)   **Age-adjusted IR of cervical cancer:**   - WWH: 204.2 (95% CI: 83.8–324.7) per 100,000 person-years - Without HIV: 31.2 (95% CI: 17.9–44.5) per 100,000 person-years   **IRR of cervical cancer:** 6.55 (95% CI: 3.2–13.6) |
| **Jain *et al.*, 2021^55^**  **India** | NR | WWH (N=50) and matched controls without HIV (N=50)  Mean age: 32.7 years (range: 22–52) | **Prevalence of squamous cell carcinoma:**   - WWH: 5 (10.0%) - Without HIV: 1 (2.0%)   **Prevalence of adenocarcinoma:**   - WWH: 0 (0.0%) - Without HIV: 1 (2.0%) |
| **Klein *et al.*, 2019^60^**  **US** | January 2003–April 2019 | WWH (N=70) and matched controls without HIV (N=70)  Median age: 66 years (IQR: 65–68) | **Prevalence of cervical carcinoma:**   - WWH: NR (<1.0%) - Without HIV: NR (<1.0%) |
| **Lewis *et al.*, 2022^137^**  **Malawi** | June 2017–November 2019 | WWH (N=1,305) and women without HIV receiving first time VIA screening (N=100)  Mean age: NR (range: NR) | **Prevalence of suspected cervical cancer:**   - WWH: 13 (1.0%) - Without HIV: 0 (0.0%) |
| **Mbuya *et al.*, 2020^47^**  **Tanzania** | 2013–2017 | WWH (N=215) and women without HIV (N=240)  Median age:   - WWH: 38 years (IQR: 31–45 years) - Without HIV: 38 years (IQR: 29–48 years)   [200 WWH and 240 women without HIV with cytology data] | **Prevalence of cervical cancer:**   - WWH: 16 (8.0%) - Without HIV: 16 (7.0%) |
| **Nakisige *et al.*, 2022^138^**  **Uganda** | July 2017–February 2020 | WWH (N=188) and women without HIV (N=116)  Median age:   - WWH: 34 years (IQR: 28–40 years) - Without HIV: 33 years (IQR: 25–43 years) | **Prevalence of invasive cervical cancer:**   - WWH: 13 (7.0%) - Without HIV: 24 (21.0%) |
| **Ndizeye *et al.*, 2019^139^**  **Burundi** | Kirundo: May 2013–July 2013  Bujumbura: March 2016–May 2016 | Urban WWH (N=151) and women without HIV (N=149)  Mean age:   - WWH: 41.1 years (SD: 9.7) - Without HIV: 39.7 years (SD: 8.7) | **Prevalence of invasive cervical cancer:**   - WWH: 1 (0.7%) - Without HIV: 0 (0.0%) |
| **Rositch *et al.*, 2021^155^**  **US** | 1996–2016 | WWH (N=609) and women without HIV (N=62,006) with cervical cancer  Mean age:   - WWH: 43.6 years (range: NR) - Without HIV: 51.2 years (range: NR) | **Prevalence of adenocarcinoma:**   - WWH: 35 (NR) - Without HIV: 11,332 (NR)   **Prevalence of squamous cell carcinoma:**   - WWH: 507 (NR) - Without HIV: 42,987 (NR)   **Five-year overall survival of adenocarcinoma:**   - WWH: 46.2% (95% CI: 27.0–65.4%) - Without HIV: 64.7% (95% CI: 63.5–65.9%)   **Five-year overall survival of squamous cell carcinoma:**   - WWH: 43.8% (95% CI: 38.7–48.8%) - Without HIV: 58.5% (95% CI: 57.9–59.1%)   **Standardized incidence ratio of adenocarcinoma:** 1.47 (95% CI: 1.03–2.05)  **Standardized incidence ratio of squamous cell carcinoma:** 3.62 (95% CI: 3.31–3.94)  **Standardized incidence ratio of adenosquamous carcinoma:** 1.83 (95% CI: 0.88–3.36)  **Standardized incidence ratio of other/unknown cervical cancer:** 3.89 (95% CI: 2.95–5.05) |
| **Non-comparative** | | | |
| **Chaussaude *et al.*, 2021^156^**  **France** | January 2010–December 2017 | WWH (N=6)  Mean age: NR (range: NR) | **IR of cervical cancer:** 26.3 per 100,000 person-years |
| **Guthrie *et al.*, 2020^4^**  **Kenya** | September 2007–December 2009 | WWH in HIV-discordant couples (N=305)  Mean age: 28 years (range: 18–50 years)  [268 WWH with cervical cytological results] | **Prevalence of cervical squamous cell carcinoma:** 2 (0.8%) |
| **Khalil *et al.*, 2021^157^**  **Multinational** | 2020 | WWH (N=NR)  Mean age: NR (range: NR) | **IR of cervical cancer:**   - South Africa: 15.8 per 100,000 person-years - East Africa: 9.4 per 100,000 person-years - Central Africa: 3.2 per 100,000 person years - Western Africa: 2 per 100,000 person-years |
| **Kremer *et al.*, 2018^5^**  **South Africa** | February 2013–March 2015 | WWH (N=463)  Mean age: NR (range: NR) | **Prevalence of invasive cervical cancer:** 22 (NR) |
| **Ortiz *et al.*, 2018^158^**  **US** | 1996–2012 | (N=NR; 502 HPV-related cancers in 864,067 person-years of follow-up among HIV-infected Hispanics)  Mean age: NR (range: NR)  [137 cases of cervical cancer] | **SIR of cervical cancer:** 3.59 (95% CI: 3.02–4.25)  **IR of cervical cancer:**   - Hispanic WWH: 56.0 per 100,000 person-years - Non-Hispanic white WWH: 33.1 per 100,000 person-years - Non-Hispanic black WWH: 47.5 per 100,000 person-years   **Five-year survival:**   - Hispanic WWH: 53% - Non-Hispanic white WWH: 52% - Non-Hispanic black WWH: 46% |
| **Oulahdlahsen *et al.*, 2018^152^**  **Morrocco** | February 2013–September 2016 | WWH (N=251)  Median age: 39 years (IQR: 32–47 years)  [246 WWH with cervical cytological results] | **Prevalence of in situ cervical cancer:** 0 (0.0%) |
| **Shin *et al.*, 2019^150^**  **India** | February 2018–March 2018 | WWH receiving ART (N=598)  Median age: 37 years (IQR: 32–42 years) | **Prevalence of squamous cell carcinoma:** 4 (0.7%) |
| **Ye *et al.*, 2020^143^**  **US** | January 2006–March 2017 | WWH (N=1,038)  Mean age: 41.8 years (SD: 10.6) | **IR of cervical cancer:** 22.62 (95% CI: 9.8–35.4) per 10,000 person-years |
| **Zhou *et al.*, 2023^159^**  **US** | 2012 | WWH (N=72,508) Medicaid enrollees 18–64 years  Mean age: NR (range: 18–64 years) | **Prevalence of cervical cancer:** 837 (1.5%) |

Abbreviations: CI: Confidence interval; HIV: human immunodeficiency virus; HPV: human papillomavirus; IQR: interquartile range; IR: incidence rate; IRR: incidence rate ratio; LSIL: low-grade squamous intraepithelial lesions; NR: not reported; SCC: squamous cell carcinoma; SD: standard deviation; SIR: standardized incidence ratio; US: United States; VIA: visual inspection with acetic acid; WWH: women with HIV.

### Supplementary Table 21. Studies reporting age stratified incidence or prevalence among WWH

| **Publication, country** | **Age, years** | **Outcome** |
| --- | --- | --- |
| **Chaussade *et al.*, 2021^156^**  **(N=6)**  **France** | 18–39 | **Incidence rate of cervical cancer:** 0.0 per 100,000 person-years |
|  | 40–49 | **Incidence rate of cervical cancer:** 39.8 per 100,000 person-years |
|  | 50–59 | **Incidence rate of cervical cancer:** 27.2 per 100,000 person-years |
|  | ≥60 | **Incidence rate of cervical cancer:** 27.6 per 100,000 person-years |
| **Khalil *et al.*, 2021^157^**  **(N=NR)**  **Multinational** | 25–34 | **Incidence rate of cervical cancer:**   - Southern Africa: 22.1 per 100,000 person-years - Eastern Africa: 10.4 per 100,000 person-years - Central Africa: 3.8 per 100,000 person-years - Western Africa: 3.0 per 100,000 person-years |
|  | 35–44 | **Incidence rate of cervical cancer:**   - Southern Africa: 40.8 per 100,000 person-years - Eastern Africa: 19.6 per 100,000 person-years - Central Africa: 6.8 per 100,000 person-years - Western Africa: 4.2 per 100,000 person-years |
|  | 45–54 | **Incidence rate of cervical cancer:**   - Southern Africa: 42.4 per 100,000 person-years - Eastern Africa: 23.7 per 100,000 person-years - Central Africa: 7.0 per 100,000 person-years - Western Africa: 4.2 per 100,000 person-years |
|  | ≥55 | **Incidence rate of cervical cancer:**   - Southern Africa: 12.8 per 100,000 person-years - Eastern Africa: 8.5 per 100,000 person-years - Central Africa: 2.2 per 100,000 person-years - Western Africa: 1.4 per 100,000 person-years |
| **Rositch *et al.*, 2021^155^**  **(N=609)**  **US** | <35 | Prevalence of all cervical cancers: 15.4% |
|  | 35–44 | Prevalence of all cervical cancers: 44.8% |
|  | 45–54 | Prevalence of all cervical cancers: 30.9% |
|  | ≥55 | Prevalence of all cervical cancers: 8.9% |

Abbreviations: HIV: Human immunodeficiency virus; NR: not reported; WWH: women with HIV; US: United States.

### Supplementary Table 22. Comparative and non-comparative studies reporting on the prevalence or incidence of any anal abnormality in PWH and PWoH

| **Publication, country** | **Date of data collection** | **Population** | **Outcome** |
| --- | --- | --- | --- |
| **Comparative** | | | |
| **Cavallari *et al.*, 2023^65^**  **Italy** | NR | WWH (N=26) and women without HIV (N=74)  Median age: 45 years (range: NR) | **Prevalence of normal anal cytology:**   - WWH: NR (54.0%) - Without HIV: NR (86.0%) - p=0.01   **Prevalence of cytological LSIL:**   - WWH: NR (40.0%) - Without HIV: NR (12.0%) - p=0.017   **Prevalence of biopsy-proven HSIL:**   - WWH: NR (20.0%) - Without HIV: NR (4.0%) - p=0.01   **Prevalence of biopsy-proven LSIL:**   - WWH: NR (33.0%) - Without HIV: NR (22.0%) - p=0.23 |
| **Furukawa *et al.*, 2018^160^**  **Japan** | November 2013–April 2015 | MWH (N=28) and men without HIV with anal condyloma acuminatum (N=34)  Mean age: 35.1 years (SD: 11.6) | **Prevalence of intra-anal canal condyloma acuminatum lesion:**   - MWH: 26 (92.9%) - Without HIV: 18 (54.5%) - p=0.0012   **Prevalence of HSIL in condyloma acuminatum:**   - MWH: 7 (25.0%) - Without HIV: 2 (6.5%) - p=0.712   **Prevalence of recurrent condyloma acuminatum:**   - MWH: 9 (33.3%) - Without HIV: 13 (43.3%) - p=0.5868 |
| **Gupta *et al.*, 2022^122^**  **India** | October 2019–October 2021 | WWH (N=141) and women without HIV (N=161) aged 21–70 years  Mean age:   - WWH: 36.3 years (range: NR) - women without HIV: 35.2 years (range: NR)   [135 WWH and 160 women without HIV were included within the analysis] | **Prevalence of cytological abnormalities (ASC-US):**   - WWH: NR (3.7%) - Without HIV: NR (1.8%) - p=0.33 |
| **Shiojiri *et al.*, 2021^95^**  **Japan** | January 2019–August 2019 | MSM with HIV (N=437) and MSM without HIV (N=207)  Median age:   - MSM with HIV: 46 years (IQR: 40–53) - Without HIV: 36 years (IQR: 29–43) | **Prevalence of abnormal anal cytology (ASC-US):**   - MSM with HIV: NR (86.6%) - MSM without HIV: NR (62.3%) - P<0.001 |
| **Non-comparative** | | | |
| **Bhardwaj *et al.*, 2022^120^**  **US** | 2015–2020 | MSM with HIV <35 years (N=516)  Mean age: 28 years (range: 18–34) | - **Prevalence of ASC-US cytology:** NR (41.0%) - **Prevalence of LSIL cytology**: NR (34.0%) - **Prevalence of ASC-H/HSIL cytology**: NR (8.0%) - **Prevalence of LSIL biopsy:** NR (40.0%) - **Prevalence of HSIL biopsy:** NR (52.0%) - **Prevalence of AIN2 biopsy**: NR (35.0%) - **Prevalence of AIN3 biopsy:** NR (17.0%) |
| **Liu *et al.*, 2023^39^**  **US** | 2012–2019 | WWH (N=381)  Median age: 49 years (IQR: 20–81) | **Prevalence of abnormal anal cytology:** NR (44.0%) |
| **Geba *et al.*, 2022^161^**  **US** | December 2017–May 2021 | PWH (N=317)  Mean age: 51 (SD: 11) | **Prevalence of abnormal anal cytology:** 152 (48.0%) |
|  |  | PWH with abnormal cytology (N=152) | **Prevalence of LSIL/ASC-US:** 142 (93.0%)  **Prevalence of HSIL/ASC-H:** 10 (7.0%) |
|  |  | PWH with biopsy results (N=136) | **Prevalence of histological AIN2+:** 84 (62.0%) |
| **Marra *et al.*, 2019^113^**  **The Netherlands** | First data set: 2010–2011  Second data set: up to December 2015 | MSM with HIV (N=193)  Mean age: 50 years (SD: 10) | **Prevalence of HSIL:** 50 (26.0%) |
| **Revollo *et al.*, 2019^79^**  **Spain** | NR | MLWLH (N=251)  Median age: 43 years (IQR: 37–48)  [Median follow-up: 4 years] | **Cumulative incidence of ASC-US:** 16 (6.0%)  **Cumulative incidence of SIL (LSIL/HSIL):** 107 (43.0%; 95% CI: 36–49%)  **Cumulative incidence of LSIL:** 92 (37.0%; 95% CI: 31–43%)  **Cumulative incidence of HSIL:** 15 (6.0%; 95% CI: 3–10%) |
| **Ye *et al.*, 2020^143^**  **US** | January 2006–March 2017 | PWH diagnosed ≥18 years (N=4,484) with HPV-related clinical conditions  Mean age: 41.8 years (SD: 10.6) | **Incidence of anal LSIL:** 194.5 per 10,000 person-years  **Incidence of anal HSIL:** 33.8 per 10,000 person-years |

Abbreviations: AIN: Anal intraepithelial neoplasia; ASC-US: atypical squamous cells of undetermined significance; CI: confidence interval; HIV: human immunodeficiency virus; HPV: human papillomavirus; HSIL: high-grade squamous intraepithelial lesion; LSIL: low-grade squamous intraepithelial lesion; MSM: men who have sex with men; NR: not reported; PWH: people with HIV; PWoH: people without HIV; SD: standard deviation; WWH: women with HIV.

### Supplementary Table 23. Comparative and non-comparative studies reporting on the burden of anal cancer in PWH and PWoH

| **Publication, country** | **Date of data collection** | **Population** | **Outcome** |
| --- | --- | --- | --- |
| **Comparative** | | | |
| **Clark *et al.*, 2020^154^**  **US** | October 1999–December 2016 | Veteran women with (N=1,454) and without (N=5,816) HIV  Mean age: NR (range: NR) | **Prevalence of anal/rectal cancer:**   - WWH: 2 (NR) - Without HIV: 5 (NR)   **Age adjusted IR of anal/rectal cancer:**   - WWH: 12.6 (95% CI: 0.0–30.9) per 100,000 person-years - Without HIV: 8.35 (95% CI: 0.8–15.9) per 100,000 person-years |
| **Finkelman *et al.*, 2021^162^**  **US** | 2010–2019 | PWH (N=940) and PWoH (N=1,263) with at least one diagnosis of anorectal SIL  Mean age: NR (range: NR) | **HR of HSIL to CIS+ progression:** 1.43 (p=0.02)  **HR of CIS to SCC progression:** 2.08 (p=0.03) |
| **Non-comparative** | | | |
| **Brogden *et al.*, 2022^163^**  **England** | 2013–2017 | PWH within the Clinical Outcomes and Services Dataset with HPV-related perineal malignancies (N=NR)  Mean age: NR (range: NR) | **Incidence increase of anal squamous cell carcinoma:**   - Women: 29.0% - Men: 14.5% |
| **Cerejeira *et al.*, 2020^164^**  **Portugal** | January 2010–June 2018  (Follow-up: NR) | MSM with HIV (N=78)  Mean age:   - With perianal warts: 32.9 years (SD: 10.4) - Without perianal warts: 35.4 years (SD: 8.1) | **Incident in-situ or invasive anal carcinoma:** 0 (0.0%) |
| **Chaussade *et al.*, 2021^156^**  **France** | January 2010–December 2017 | PWH (N=11)  Mean age: NR (range: NR) | **IR of anal cancer:** 48.3 per 100,000 person-years |
| **Ortiz *et al.*, 2018^158^**  **US** | 1996–2012 | PWH with cancer (N=NR; 502 HPV-related cancers in 864,067 person-years of follow-up among HIV-infected Hispanics)  Mean age: NR (range: NR)  [62 cases of WWH with anal cancer and 218 cases of MWH with anal cancer] | **SIR of anal cancer:**   - Hispanic WWH: 13.2 (95% CI: 10.1–16.9) - Hispanic MWH: 18.7 (95% CI: 16.3–21.3)   **Incidence rate of anal cancer:**   - Hispanic WWH: 25.3 per 100,000 person-years - Hispanic MWH: 35.2 per 100,000 person-years - Non-Hispanic white WWH: 27.2 per 100,000 person-years - Non-Hispanic white MWH: 83.4 per 100,000 person-years - Non-Hispanic black WWH: 20.0 per 100,000 person-years - Non-Hispanic black MWH: 51.7 per 100,000 person-years   **Five-year survival of anal cancer:**   - Hispanic: NR (59.0%) - Non-Hispanic white: NR (48.0%) - Non-Hispanic black: NR (35.0%) |
| **Waters *et al.*, 2023^165^**  **US** | 1996–2015 | PWH (N=198)  Mean age: NR (range: NR) | **Prevalence of anal cancer:**   - MWH: 187 (94.4%) - WWH: 11 (5.6%) |
| **Ye *et al.*, 2020^143^**  **US** | January 2006–March 2017 | PWH diagnosed ≥18 years (N=4,484) with HPV-related clinical conditions  Mean age: 41.8 years (SD: 10.6) | **Incidence of anal cancer:** 19.4 (95% CI: 13.6–25.2) per 10,000 person-years   - MWH: 25.8 per 10,000 person-years - WWH: 0.0 per 10,000 person-years |

Abbreviations: AIN: Anal intraepithelial neoplasia; ASC-US: atypical squamous cells of undetermined significance; CI: confidence interval; CIS: carcinoma in situ; HIV: human immunodeficiency virus; HPV: human papillomavirus; HR: hazard ratio; IR: incidence rate; MSM: men who have sex with men; NR: not reported; PWH: people with HIV; PWoH: people without HIV; SCC: squamous cell carcinoma; SD: standard deviation; WWH: women with HIV.

### Supplementary Table 24. Non-comparative studies reporting on the prevalence of HPV-related head and neck/oral cancers among PWH

| **Publication, country** | **Date of data collection** | **Population** | **Outcome** |
| --- | --- | --- | --- |
| **Comparative** | | | |
| **Chaussade *et al.*, 2021^156^**  **France** | January 2010–December 2017 | N=10,703 [PWH with head and neck cancer (N=18)  Mean age: NR (range: NR)  [18 cases of head and neck cancer] | **IR of head and neck cancer:**   - PWH: 79.1 per 100,000 person-years - Without HIV: 30.7 per 100,000 person-years |
| **Non-comparative** | | | |
| **Ortiz *et al.*, 2018^158^**  **US** | 1996–2012 | MWH (N=NR; 502 HPV-related cancers in 864,067 person-years of follow-up among HIV-infected Hispanics)  Mean age: NR (range: NR)  [8 cases of oropharynx cancer] | **SIR of oropharynx cancer in Hispanic MWH versus the general US population of Hispanics without HIV:** 1.59 (95% CI: 0.69–3.14)  **IR of oropharynx cancer:**   - Hispanic MWH: 1.29 per 100,000 person-years - Non-Hispanic white MWH: 0.77 per 100,000 person-years - Non-Hispanic black MWH: 2.47 per 100,000 person-years |
| **Picard *et al.*, 2018^166^**  **France** | January 2015–March 2015 | PWH with head and neck cancers (N=286)  Median age: 52 years (range: NR) | **Prevalence of any oropharyngeal cancer:** 116 (41.0%) |
|  |  | PWH with oropharyngeal cancer (N=116) | **Incidence of potential HPV-related head and neck squamous cell carcinoma cases:** 0.78 supplementary cases per year   - p=0.007   **Prevalence of palatine tonsil cancer:** NR (43.0%)  **Prevalence of cancer of the oropharynx:** NR (26.0%)  **Prevalence of cancer on the base of the tongue:** NR (16.0%) |
| **Waters *et al.*, 2023^165^**  **US** | 1996–2015 | PWH with HPV-associated cancer (N=355)  Mean age: NR (range: NR) | **Prevalence of oropharyngeal cancer:** 13 (3.9%) |

Abbreviations: HIV: Human immunodeficiency virus; HPV: human papillomavirus; IR: incidence rate; MWH: men with HIV; NR: not reported; PWH: people with HIV; SIR: standardized incidence ratio; US: United States; WWH: women with HIV.

### Bibliography

1. Adebamowo SN, Adeyemo AA. Classical HLA alleles are associated with prevalent and persistent cervical high-risk HPV infection in African women. *Human immunology*. 2019;80(9):723-730. doi:<https://dx.doi.org/10.1016/j.humimm.2019.04.011>

2. Chachage M, Parikh AP, Mahenge A, et al. High-risk human papillomavirus genotype distribution among women living with and at risk for HIV in Africa. *AIDS*. 2023;37(4):625-635. doi:<https://dx.doi.org/10.1097/QAD.0000000000003437>

3. Ferre VM, Ekouevi DK, Gbeasor-Komlanvi FA, et al. Prevalence of human papillomavirus, human immunodeficiency virus and other sexually transmitted infections among female sex workers in Togo: a national cross-sectional survey. *Clinical Microbiology and Infection*. 2019;25(12):1560.e1-1560.e7. doi:<https://dx.doi.org/10.1016/j.cmi.2019.04.015>

4. Guthrie BL, Rositch AF, Cooper JA, et al. Human papillomavirus and abnormal cervical lesions among HIV-infected women in HIV-discordant couples from Kenya. *Sexually Transmitted Infections*. 2020;96(6):457-463. doi:<https://dx.doi.org/10.1136/sextrans-2019-054052>

5. Kremer WW, van Zummeren M, Heideman DAM, et al. HPV16-related cervical cancers and precancers have increased levels of host cell DNA methylation in women living with HIV. *International Journal of Molecular Sciences*. 2018;19(11):3297. doi:<https://dx.doi.org/10.3390/ijms19113297>

6. Luckett R, Painter H, Hacker MR, et al. Persistence and clearance of high-risk human papillomavirus and cervical dysplasia at 1 year in women living with human immunodeficiency virus: a prospective cohort study. *BJOG: An International Journal of Obstetrics and Gynaecology*. 2021;128(12):1986-1996. doi:<https://dx.doi.org/10.1111/1471-0528.16758>

7. Mandiriri AM, Pascoe MJ, Shamu T, Lowe S. Cervical human papillomavirus prevalence, risk factors and outcomes in a cohort of HIV-infected women in Harare, Zimbabwe. *Southern African Journal of HIV Medicine*. 2020;21(1):a1123. doi:<https://dx.doi.org/10.4102/SAJHIVMED.V21I1.1123>

8. Megersa T, Dango S, Kumsa K, Lemma K, Lencha B. Prevalence of high-risk human papillomavirus infections and associated factors among women living with HIV in Shashemene town public health facilities, Southern Ethiopia. *BMC Women's Health*. 2023;23(1):125. doi:<https://dx.doi.org/10.1186/s12905-023-02279-2>

9. Nyasenu YT, Gbeasor-Komlanvi FA, Ehlan A, et al. Prevalence and distribution of Human Papillomavirus (HPV) genotypes among HIV infected women in Lome, Togo. *PloS one*. 2019;14(2):e0212516. doi:<https://dx.doi.org/10.1371/journal.pone.0212516>

10. Rais M, Ouyahia A, Mohammedi D, et al. First study of genital HPV infection among women living with HIV recruited from May to September 2018 in Eastern Algeria. *International journal of STD & AIDS*. 2023:9564624231179274. doi:<https://dx.doi.org/10.1177/09564624231179274>

11. Uwamungu S, Nigussie B, Muvunyi CM, Hasseus B, Andersson M, Giglio D. Prevalence of Human Papillomavirus in Different Mucous Membranes in HIV Concordant Couples in Rwanda. *Viruses*. 2023;15(4):1005. doi:<https://dx.doi.org/10.3390/v15041005>

12. Vyankandondera J, Wambua S, Irungu E, et al. Type-Specific Human Papillomavirus Prevalence, Incident Cases, Persistence, and Associated Pregnancy Outcomes among HIV-Infected Women in Kenya. *Sexually Transmitted Diseases*. 2019;46(8):532-539. doi:<https://dx.doi.org/10.1097/OLQ.0000000000001029>

13. Yakub MM, Fowotade A, Anaedobe CG, Manga MM, Bakare RA, Abimiku BA. Human papillomavirus correlates of high grade cervical dysplasia among HIV-Infected women at a major treatment centre in Nigeria: A cross-sectional study. *Pan African Medical Journal*. 2019;33:125. doi:<https://dx.doi.org/10.11604/pamj.2019.33.125.17589>

14. Aziz H, Sattar AA, Mahmood H, Fatima S, Khurshid M, Faheem M. Prevalence of HPV types in HIV-positive and negative females with normal cervical cytology or dysplasia. *Journal of Clinical Laboratory Analysis*. 2023;37(4):e24851. doi:<https://dx.doi.org/10.1002/jcla.24851>

15. Isaguliants M, Nosik M, Karlsen A, et al. Prevalence and Risk Factors of Infection with High Risk Human Papilloma Viruses among HIV-Positive Women with Clinical Manifestations of Tuberculosis in a Middle-Income Country. *Biomedicines*. 2021;9(6)doi:<https://dx.doi.org/10.3390/biomedicines9060683>

16. Pahwa V, Pimple SA, Mishra GA, Anand KV. Prevalence and Determinants of Human Papilloma Virus Infection and Cervical Intraepithelial Neoplasia (CIN) among Women Living with HIV/AIDS in Mumbai, India. *Indian Journal of Medical and Paediatric Oncology*. 2022;43(1):97-102. doi:<https://dx.doi.org/10.1055/s-0042-1742661>

17. Qiao YP, Wang AL, Fang LW, Hann K, Wang LH. High-risk Human Papillomavirus Infection and Associated Factors among HIV-positive Women in High HIV-burden Areas of China. *Biomedical and environmental sciences : BES*. 2020;33(3):206-212. doi:<https://dx.doi.org/10.3967/bes2020.029>

18. Risnawati, Soejoenoes A, Hadisaputro S, et al. Hpv 52 most dominant human papillomavirus (Hpv) genotype in women infected by human immunodeficiency virus (hiv) that get antiretroviral treatment (art). *International Journal of Pharmaceutical Research*. 2020;12(4):2568-2573. doi:<https://dx.doi.org/10.31838/ijpr/2020.12.04.355>

19. Wang Q, Ma X, Zhang X, et al. Human papillomavirus infection and associated factors for cervical intraepithelial neoplasia in women living with HIV in China: a cross-sectional study. *Sexually transmitted infections*. 2019;95(2):140-144. doi:<https://dx.doi.org/10.1136/sextrans-2018-053636>

20. Abel S, Najioullah F, Volumenie JL, et al. High prevalence of human papillomavirus infection in HIV-infected women living in French Antilles and French Guiana. *PLoS ONE*. 2019;14(9):e0221334. doi:<https://dx.doi.org/10.1371/journal.pone.0221334>

21. Badial RM, Dias MC, Stuqui B, et al. Detection and genotyping of human papillomavirus (HPV) in HIV-infected women and its relationship with HPV/HIV co-infection. *Medicine*. 2018;97(14):e9545. doi:<https://dx.doi.org/10.1097/MD.0000000000009545>

22. Kury CMH, Santos KCDS, Carestiato FN, et al. Human papillomavirus prevalence, genomic diversity and related risk factors in HIV-positive women from a countryside city in the state of Rio de Janeiro. *Human Vaccines and Immunotherapeutics*. 2021;17(3):838-844. doi:<https://dx.doi.org/10.1080/21645515.2020.1799666>

23. Monteiro JC, Fonseca RRdS, Ferreira TCdS, et al. Prevalence of High Risk HPV in HIV-Infected Women From Belem, Para, Amazon Region of Brazil: A Cross-Sectional Study. *Frontiers in public health*. 2021;9:649152. doi:<https://dx.doi.org/10.3389/fpubh.2021.649152>

24. Ortiz-Gutierrez F, Sanchez-Minutti L, Martinez-Herrera JF, et al. Identification of Genetic Variants of Human Papillomavirus in a Group of Mexican HIV/AIDS Patients and Their Possible Association with Cervical Cancer. *Polish Journal of Microbiology*. 2021;70(4):501-509. doi:<https://dx.doi.org/10.33073/pjm-2021-047>

25. Perez-Quintanilla M, Mendez-Martinez R, Vazquez-Vega S, et al. High prevalence of human papillomavirus and European variants of HPV 16 infecting concomitantly to cervix and oral cavity in HIV positive women. *PloS one*. 2020;15(4):e0227900. doi:<https://dx.doi.org/10.1371/journal.pone.0227900>

26. Teixeira MF, Sabido M, Leturiondo AL, de Oliveira Ferreira C, Torres KL, Benzaken AS. High risk human papillomavirus prevalence and genotype distribution among women infected with HIV in Manaus, Amazonas. *Virology journal*. 2018;15(1):36. doi:<https://dx.doi.org/10.1186/s12985-018-0942-6>

27. Tosato Boldrini NA, Bondi Volpini LP, Freitas LB, et al. Sexually transmitted infections among women living with HIV in a Brazilian city. *Brazilian Journal of Infectious Diseases*. 2021;25(1):101044. doi:<https://dx.doi.org/10.1016/j.bjid.2020.101044>

28. Boudes M, Venard V, Routiot T, Buzzi M, Maillot F. Prevalence and distribution of hpv genotypes in immunosuppressed patients in lorraine region. *Viruses*. 2021;13(12):2454. doi:<https://dx.doi.org/10.3390/v13122454>

29. Cambrea SC, Aschie M, Resul G, et al. HPV and HIV Coinfection in Women from a Southeast Region of Romania-PICOPIV Study. *Medicina (Kaunas, Lithuania)*. 2022;58(6)doi:<https://dx.doi.org/10.3390/medicina58060760>

30. Carriero C, Fascilla FD, Cramarossa P, Lepera A, Bettocchi S, Vimercati A. Colpocytological abnormalities in HIV infected and uninfected pregnant women: prevalence, persistence and progression. *Journal of Obstetrics and Gynaecology*. 2018;38(4):526-531. doi:<https://dx.doi.org/10.1080/01443615.2017.1373082>

31. Elliott T, Henderson M, Crook E, et al. High-risk HPV prevalence and serostatus in women living with perinatally acquired HIV (the SHiP study). *HIV Medicine*. 2023;24(Supplement 3):12. Spring Conference of the British HIV Association, BHIVA 2023. Gateshead United Kingdom. doi:<https://dx.doi.org/10.1111/hiv.13476>

32. Metz CKS, Skof AS, Sehouli J, et al. Assessment of High-Risk Human Papillomavirus Infections and Cervical Dusplasia in Human Immunodeficiency Virus-Positive Pregnany Women in Germany: a Prospective Cross-Sectional Two Center Study. *International Journal of Gynecological Cancer*. 2022;32(Supplement 2):A37-A38. European Congress on Gynaecological Oncology, ESGO 2022. Berlin Germany. doi:<https://dx.doi.org/10.1136/ijgc-2022-ESGO.79>

33. Orlando G, Frati ER, Fasolo MM, et al. Incident genital HPV infections and potential impact of HPV vaccines in adult women living with HIV/AIDS. *Human Vaccines and Immunotherapeutics*. 2019;15(7-8):1904-1910. doi:<https://dx.doi.org/10.1080/21645515.2018.1528834>

34. Thorsteinsson K, Storgaard M, Katzenstein TL, et al. Prevalence of cervical, oral, and anal human papillomavirus infection in women living with HIV in Denmark - The SHADE cohort study. *Journal of clinical virology : the official publication of the Pan American Society for Clinical Virology*. 2018;105:64-71. doi:<https://dx.doi.org/10.1016/j.jcv.2018.05.010>

35. Al-Lawati Z, Khamis FA, Al-Hamdani A, et al. Prevalence of human papilloma virus in Oman: Genotypes 82 and 68 are dominating. *International Journal of Infectious Diseases*. 2020;93:22-27. doi:<https://dx.doi.org/10.1016/j.ijid.2019.12.038>

36. Al Shaarani M, Lu D, Szporn A, Zakowski M, Si Q. Prevalence of high-risk hpv and squamous lesions in concurrent anal and cervical cytology specimens from the same HIV infected women. *Modern Pathology*. 2020;33(3):321-322. 109th Annual Meeting of the United States and Canadian Academy of Pathology, USCAP 2020. Los Angeles, CA United States.

37. Cameron JE, Dennis DAC, Herrel NR, Chapple AG, Hagensee ME. Risk of abnormal cervical cytology in HIV-infected women testing positive for both human papillomavirus and Epstein-Barr virus in genital tract specimens. *Cancer Causes and Control*. 2020;31(4):365-375. doi:<https://dx.doi.org/10.1007/s10552-020-01287-z>

38. Covarrubias AS, Duthely L, Potter J, Bahadue F. Risk factors and pap progression among minority women living with HIV and negative baseline cervical cytology. *Journal of Lower Genital Tract Disease*. 2020;24(Supplement 1):S20. 2020 Annual Meeting of the American Society for Colposcopy and Cervical Pathology, ASCCP 2020. Orlando, FL United States. doi:<https://dx.doi.org/10.1097/LGT.0000000000000538>

39. Liu Y, Weiss K, Zamudio AR, et al. Primary Anal Cancer Screening Results From 381 Women With Human Immunodeficiency Virus. *The Journal of infectious diseases*. 2023;227(8):932-938. doi:<https://dx.doi.org/10.1093/infdis/jiac398>

40. Kaufman E, Williamson T, Mayrand M-H, et al. Identifying risk factors for prevalent anal human papillomavirus type 16 infection in women living with HIV. *PloS one*. 2022;17(5):e0268521. doi:<https://dx.doi.org/10.1371/journal.pone.0268521>

41. Ermel A, Tonui P, Titus M, et al. A cross-sectional analysis of factors associated with detection of oncogenic human papillomavirus in human immunodeficiency virus-infected and uninfected Kenyan women. *BMC infectious diseases*. 2019;19(1):352. doi:<https://dx.doi.org/10.1186/s12879-019-3982-7>

42. Hopkins KL, Jaffer M, Hlongwane KE, et al. Assessing national cervical cancer screening guidelines: Results from an HIV testing clinic also screening for cervical cancer and HPV in Soweto, South Africa. *PloS one*. 2021;16(7):e0255124. doi:<https://dx.doi.org/10.1371/journal.pone.0255124>

43. Jary A, Teguete I, Sidibe Y, et al. Prevalence of cervical HPV infection, sexually transmitted infections and associated antimicrobial resistance in women attending cervical cancer screening in Mali. *International journal of infectious diseases : IJID : official publication of the International Society for Infectious Diseases*. 2021;doi:<https://dx.doi.org/10.1016/j.ijid.2021.06.024>

44. Karani LW, Musyoki S, Orina R, Nyamache AK, Khayeka-Wandabwa C, Nyagaka B. Human papillomavirus genotype profiles and cytological grades interlinkages in coinfection with HIV. *Pan African Medical Journal*. 2020;35:67. doi:<https://dx.doi.org/10.11604/pamj.2020.35.67.21539>

45. Kinotia NJ, Muturib M, Kamauc L, Lwembed R. Human Papillomavirus types prevalence and their association with cervical dysplasia among HIV and non-HIV infected women attending reproductive health clinics in Eastern Kenya. *African health sciences*. 2022;22(1):106-114. doi:<https://dx.doi.org/10.4314/ahs.v22i1.14>

46. Lieber M, Reynolds CW, Lieb W, McGill S, Beddoe AM. Human papillomavirus knowledge, attitudes, practices, and prevalence among men who have sex with men in Monrovia, Liberia. *Journal of Lower Genital Tract Disease*. 2018;22(4):326-332. doi:<https://dx.doi.org/10.1097/LGT.0000000000000436>

47. Mbuya W, McHaro R, Mhizde J, et al. Depletion and activation of mucosal CD4 T cells in HIV infected women with HPVassociated lesions of the cervix uteri. *PLoS ONE*. 2020;15(10 October):e0240154. doi:<https://dx.doi.org/10.1371/journal.pone.0240154>

48. McHome BL, Kjaer SK, Manongi R, et al. HPV types, cervical high-grade lesions and risk factors for oncogenic human papillomavirus infection among 3416 Tanzanian women. *Sexually Transmitted Infections*. 2021;97(1):56-62. doi:<https://dx.doi.org/10.1136/sextrans-2019-054263>

49. Njue JK, Muturi M, Kamau L, Lwembe R. Human Papillomavirus Types Associated with Cervical Dysplasia among HIV- And Non-HIV-Infected Women Attending Reproductive Health Clinics in Eastern Kenya. *BioMed Research International*. 2021;2021:2250690. doi:<https://dx.doi.org/10.1155/2021/2250690>

50. Okoye JO, Ngokere AA, Erinle C, Mbamalu C. Co-existence of Herpes simplex virus type 2 and two other oncoviruses is associated with cervical lesions in women living with HIV in South-Western Nigeria. *African health sciences*. 2020;20(3):1015-1023. doi:<https://dx.doi.org/10.4314/ahs.v20i3.4>

51. Omire A, Budambula NLM, Kirumbi L, et al. Cervical Dysplasia, Infection, and Phylogeny of Human Papillomavirus in HIV-Infected and HIV-Uninfected Women at a Reproductive Health Clinic in Nairobi, Kenya. *BioMed Research International*. 2020;2020:4945608. doi:<https://dx.doi.org/10.1155/2020/4945608>

52. Swai P, Kjaer S, Mchome B, et al. The association between human papillomavirus and cervical high-grade cytology among HIV-positive and HIV-negative Tanzanian women: A cross-sectional study. *Acta Obstet Gynecol Scand*. 2021;100(4):775-785. doi:10.1111/aogs.14102.

53. Sweet K, Bosire C, Sanusi B, et al. Prevalence, incidence, and distribution of human papillomavirus types in female sex workers in Kenya. *International journal of STD & AIDS*. 2020:956462419884454. doi:<https://dx.doi.org/10.1177/0956462419884454>

54. Taku O, Mbulawa ZZA, Phohlo K, Garcia-Jardon M, Businge CB, Williamson A-L. Distribution of Human Papillomavirus (HPV) Genotypes in HIV-Negative and HIV-Positive Women with Cervical Intraepithelial Lesions in the Eastern Cape Province, South Africa. *Viruses*. 2021;13(2)doi:<https://dx.doi.org/10.3390/v13020280>

55. Jain B, Adhikary T, Sadhukhan P, Nandi A. Human papilloma virus infection of uterine cervix and spectrum of cervical pathology in human immunodeficiency virus/AIDS. *Journal of Cancer Research and Therapeutics*. 2021;17(6):1462-1467. doi:<https://dx.doi.org/10.4103/jcrt.JCRT_552_19>

56. Peyyala S, Mahey R, Vashist S, Wig N, Mathur S, Tanwar P. A comparative study of prevalence of hpv infection, precancerous lesions of cervix and vulva in hivpositive and negative women. *Indian Journal of Gynecologic Oncology*. 2021;19(3)17th World Congress of the International Federation of Colposcopy and Cervical Pathology-IFCPC 2021. Virtual. doi:<https://dx.doi.org/10.1007/s40944-021-00548-2>

57. Suehiro TT, Damke GMZF, Damke E, et al. Cervical and oral human papillomavirus infection in women living with human immunodeficiency virus (HIV) and matched HIV-negative controls in Brazil. *Infectious Agents and Cancer*. 2020;15(1):31. doi:<https://dx.doi.org/10.1186/s13027-020-00301-y>

58. Nijhawan P, Carraro A, Vita S, et al. Systemic, Mucosal Immune Activation and Psycho-Sexual Health in ART-Suppressed Women Living with HIV: Evaluating Biomarkers and Environmental Stimuli. *Viruses*. 2023;15(4):960. doi:<https://dx.doi.org/10.3390/v15040960>

59. Castle PE, Befano B, Schiffman M, et al. A comparison of high-grade cervical abnormality risks in women living with and without human immunodeficiency virus undergoing routine cervical-cancer screening. *Preventive Medicine*. 2022;162:107157. doi:<https://dx.doi.org/10.1016/j.ypmed.2022.107157>

60. Klein KL, Goron AR, Taylor G, Roque DM. Pap smear outcomes in HIV-positive women >=65 years and HIV-negative matched controls. *Gynecologic Oncology*. 2020;159(Supplement 1):183-184. Society of Gynecologic Oncology 2020 Annual Meeting on Women's Cancer. Virtual, Online. doi:<https://dx.doi.org/10.1016/j.ygyno.2020.05.280>

61. Mchome B, Linde DS, Manongi R, et al. Incident detection of human papillomavirus - a prospective follow-up study among Tanzanian women with a focus on HIV status. *International Journal of Infectious Diseases*. 2021;110:165-170. doi:<https://dx.doi.org/10.1016/j.ijid.2021.07.011>

62. Murenzi G, Tuyisenge P, Kanyabwisha F, et al. Type-specific persistence, clearance and incidence of high-risk HPV among screen-positive Rwandan women living with HIV. *Infectious Agents and Cancer*. 2021;16(1):16. doi:<https://dx.doi.org/10.1186/s13027-021-00355-6>

63. Videla S, Tarrats A, Ornelas A, et al. Incidence of cervical high-grade squamous intraepithelial lesions in HIV-1-infected women with no history of cervical pathology: up to 17 years of follow-up. *International Journal of STD and AIDS*. 2019;30(1):56-63. doi:<https://dx.doi.org/10.1177/0956462418792653>

64. Chinyowa S, Palefsky JM, Chirenje ZM, Makunike-Mutasa R, Munjoma M, Muguti GI. Anal human papillomavirus infection in HIV-positive men and women at two opportunistic infections clinics in Harare, Zimbabwe. *BMC public health*. 2018;18(1):1260. doi:<https://dx.doi.org/10.1186/s12889-018-6170-6>

65. Cavallari EN, Santinelli L, Eleonora M, et al. Increased Burden of Anal HPV-Related Potentially Cancerous Lesions in HIV+ Women *Topics in Antiviral Medicine*. 2023;31(2):411. Conference on Retroviruses and Opportunistic Infections, CROI 2023. Seattle, WA United States.

66. Hidalgo-Tenorio C, Calle-Gomez I, Moya-Megias R, et al. HPV Infection of the Oropharyngeal, Genital and Anal Mucosa and Associated Dysplasia in People Living with HIV. *Viruses*. 2023;15(5):1170. doi:<https://dx.doi.org/10.3390/v15051170>

67. Squillace N, Bernasconi DP, Lapadula G, et al. HPV 16 and 18 contribute to development of anal dysplasia in HIV infection irrespective of gender and sexual orientation. *HIV Medicine*. 2021;22(9):860-866. doi:<https://dx.doi.org/10.1111/hiv.13143>

68. Sigel K, Gaisa M, Liu Y. ANAL DYSPLASIA SCREENING IN PEOPLE WITH HIV YOUNGER THAN 35. *Sexual Health*. 2022;19(2):xxxii. 2022 Scientific Meeting of the International Anal Neoplasia Society's, IANS 2022. New York City, NY United States. doi:<https://dx.doi.org/10.1071/SHv19n2abs>

69. Conde-Ferraez L, Chan-Mezeta A, Gomez-Carballo JG, Ayora-Talavera G, Gonzalez-Losa MDR. Human Papillomavirus Genotypes Infecting the Anal Canal and Cervix in HIV+ Men and Women, Anal Cytology, and Risk Factors for Anal Infection. *Pathogens*. 2023;12(2):252. doi:<https://dx.doi.org/10.3390/pathogens12020252>

70. Boldrini NAT, Volpini LPB, De Freitas LB, et al. Anal HPV infection and correlates in HIV-Infected patients attending a sexually transmitted infection clinic in Brazil. *PLoS ONE*. 2018;13(7):e0199058. doi:<https://dx.doi.org/10.1371/journal.pone.0199058>

71. Medina-Laabes DT, Suarez-Perez EL, Guiot HM, et al. Human papillomavirus correlates with histologic anal high-grade squamous intraepithelial lesions in Hispanics with HIV. *Journal of Lower Genital Tract Disease*. 2018;22(4):320-325. doi:<https://dx.doi.org/10.1097/LGT.0000000000000416>

72. Posada DH, Acevedo LST, Arredondo MV, Vasquez GIS. High-risk human papillomavirus infection and associated factors in the anal canal of HIV-positive patients in Medellin, 2017-2018. *Revista de saude publica*. 2020;54:93. doi:<https://dx.doi.org/10.11606/s1518-8787.2020054001692>

73. Popova A, Domonova E, Pokrovskaya A, Shipulina O, Pokrovskiy V. Results of HPV testing from three anatomical locations among men with different HIV status and sexual behaviour. *Journal of the International AIDS Society*. 2020;23(SUPPL 7)HIV Glasgow. Virtual. doi:<https://dx.doi.org/10.1002/jia2.25616>

74. Cheng SH, Liao KS, Wang CC, Cheng CY, Chu FY. Multiple types of human papillomavirus infection and anal precancerous lesions in HIV-infected men in Taiwan: a cross-sectional study. *BMJ Open*. 2018;8(1)doi:10.1136/bmjopen-2017-019894.

75. Liu X, Lin H, Chen X, et al. Prevalence and genotypes of anal human papillomavirus infection among HIV-positive vs. HIV-negative men in Taizhou, China. *Epidemiology and Infection*. 2019;147:e117. doi:<https://dx.doi.org/10.1017/S0950268818003205>

76. Zhang J, Chen X, Ye Y, et al. Increased CD4+ T cell count is associated with lower anal human papillomavirus prevalence among HIV-positive male cohort in Taizhou, China: a cross-sectional study. *BMC Infectious Diseases*. 2022;22(1):250. doi:<https://dx.doi.org/10.1186/s12879-022-07251-3>

77. Ablanedo-Terrazas Y, Romero-Mora K, Gomez-Palacio M, et al. Prevalence and risk factors for oral human papillomavirus infection in Mexican HIV-infected men. *Prevalencia y factores de riesgo para infeccion oral con virus de papiloma humano en hombres mexicanos con VIH*. 2018;60(6):653-657. doi:<https://dx.doi.org/10.21149/9834>

78. Cuba LB, Tello AG, Bejarano SC. Anogenital human papillomavirus infection among hiv positive peruvian men. *Journal of the Dermatology Nurses' Association*. 2020;12(2)24th World Congress of Dermatology. Milan Italy.

79. Revollo B, Videla S, Sirera G, et al. Natural History of Anal Squamous Intraepithelial Lesions in HIV-Positive Men with Normal Baseline Cytology. *AIDS patient care and STDs*. 2019;33(11):459-465. doi:<https://dx.doi.org/10.1089/apc.2019.0186>

80. Santinelli L, Scordio M, Frasca F, et al. Lack of tlrs activation in anal cells of HIV+ men may contribute to HPV persistence. *Topics in Antiviral Medicine*. 2021;29(1):173. Conference on Retroviruses and Opportunistic Infections, CROI 2021. Virtual.

81. Vergori A, Garbuglia AR, Piselli P, et al. Oral human Papillomavirus DNA detection in HIV-positive men: Prevalence, predictors, and co-occurrence at anal site. *BMC Infectious Diseases*. 2018;18(1):25. doi:<https://dx.doi.org/10.1186/s12879-017-2937-0>

82. Patel P, Bush T, Conley L, et al. Prevalence, Incidence, and Clearance of Human Papillomavirus Types Covered by Current Vaccines in Men With Human Immunodeficiency Virus in the SUN Study. *The Journal of infectious diseases*. 2020;doi:<https://dx.doi.org/10.1093/infdis/jiz425>

83. Patel P, Bush T, Kojic EM, et al. Prevalence, Incidence, and Clearance of Anal High-Risk Human Papillomavirus Infection among HIV-Infected Men in the SUN Study. *Journal of Infectious Diseases*. 2018;217(6):953-963. doi:<https://dx.doi.org/10.1093/infdis/jix607>

84. Elfituri O, Post R, Cabay R, David O. Human Papillomavirus Infection in a Cohort of Men with Human Immunodeficiency Virus (HIV) Disease. *Journal of the American Society of Cytopathology*. 2020;9(6):S22. 68th Annual Scientific Meeting. Virtual, Online. doi:<https://dx.doi.org/10.1016/j.jasc.2020.07.045>

85. Bouassa RSM, Simaleko MM, Camengo SP, et al. Unusual and unique distribution of anal high-risk human papillomavirus (hr-hpv) among men who have sex with men living in the Central African Republic. *PLoS ONE*. 2018;13(5):e0197845. doi:<https://dx.doi.org/10.1371/journal.pone.0197845>

86. Ferre VM, Sadio A, Gbeasor-Komlanvi DF, et al. High prevalence of HPV, other STI, and anal lesions among MSM in Togo. *Topics in Antiviral Medicine*. 2023;31(2):412. Conference on Retroviruses and Opportunistic Infections, CROI 2023. Seattle, WA United States.

87. Koyalta D, Mboumba Bouassa RS, Maiga A, et al. High Prevalence of Anal Oncogenic Human Papillomavirus Infection in Young Men Who Have Sex with Men Living in Bamako, Mali. *Infectious Agents and Cancer*. 2021;16(1):51. doi:<https://dx.doi.org/10.1186/s13027-021-00385-0>

88. Murenzi G, Kim HY, Munyaneza A, et al. Anogenital Human Papillomavirus and HIV Infection in Rwandan Men Who Have Sex with Men. *Journal of Acquired Immune Deficiency Syndromes*. 2020;84(5):463-469. doi:<https://dx.doi.org/10.1097/QAI.0000000000002376>

89. Nyitray AG, Masunaga KIA, Nyoni J, Ross MW. Prevalence of and factors associated with anal high-risk human papillomavirus in urban Tanzanian men who have sex with men, 2011-2012. *International Journal of STD and AIDS*. 2022;33(7):672-679. doi:<https://dx.doi.org/10.1177/09564624221094456>

90. Oo MM, Moore S, Gibbons S, et al. High prevalence of vaccine-preventable anal human papillomavirus infections is associated with HIV infection among gay, bisexual, and men who have sex with men in Nairobi, Kenya. *Cancer medicine*. 2023;12(12):13745-13757. doi:<https://dx.doi.org/10.1002/cam4.6008>

91. Beliakov I, Senina M, Tyulenev Y, Novoselova E, Surovtsev V, Guschin A. The Prevalence of High Carcinogenic Risk of HPV Genotypes among HIV-Positive and HIV-Negative MSM from Russia. *Canadian Journal of Infectious Diseases and Medical Microbiology*. 2021;2021:6641888. doi:<https://dx.doi.org/10.1155/2021/6641888>

92. Ejaz M, Andersson S, Batool S, Ali T, Ekstrom AM. Anal human papillomavirus infection among men who have sex with men and transgender women living with and without HIV in Pakistan: Findings from a cross-sectional study. *BMJ Open*. 2021;11(11):e052176. doi:<https://dx.doi.org/10.1136/bmjopen-2021-052176>

93. Ejaz M, Mubarak M, Ali TS, Andersson S, Ekstrom AM. Human papillomavirus-associated anal squamous intraepithelial lesions in men who have sex with men and transgender women living with and without HIV in Karachi Pakistan: implications for screening and prevention. *BMC Infectious Diseases*. 2021;21(1):1163. doi:<https://dx.doi.org/10.1186/s12879-021-06850-w>

94. Lin CC, Hsieh MC, Hung HC, et al. Human papillomavirus prevalence and behavioral risk factors among HIV-infected and HIVuninfected men who have sex with men in Taiwan. *Medicine (United States)*. 2018;97(45):e13201. doi:<https://dx.doi.org/10.1097/MD.0000000000013201>

95. Shiojiri D, Mizushima D, Takano M, et al. Anal human papillomavirus infection and its relationship with abnormal anal cytology among MSM with or without HIV infection in Japan. *Scientific reports*. 2021;11(1):19257. doi:<https://dx.doi.org/10.1038/s41598-021-98720-3>

96. Somia IKA, Teeratakulpisarn N, Jeo WS, et al. Prevalence of and risk factors for anal high-risk HPV among HIV-negative and HIV-positive MSM and transgender women in three countries at South-East Asia. *Medicine (United States)*. 2018;97(10):e9898. doi:<https://dx.doi.org/10.1097/MD.0000000000009898>

97. Wang CC, Chang SL, Chu FY, Cheng CY, Cheng SH. Human papillomavirus infection and anal cytology in Taiwanese homosexual men with and without HIV infection. *Journal of Infection in Developing Countries*. 2019;13(4):318-325. doi:<https://dx.doi.org/10.3855/jidc.11162>

98. Wu P-F, Hang J-F, Strong C, et al. Anal human papillomavirus and its associations with abnormal anal cytology among men who have sex with men. *Scientific reports*. 2020;10(1):3165. doi:<https://dx.doi.org/10.1038/s41598-020-59967-4>

99. Yunihastuti E, Teeratakulpisarn N, Jeo WS, et al. Incidence, clearance, persistence and factors related with high-risk anal HPV persistence in South-East Asian MSM and transgender women. *AIDS*. 2020;34(13):1933-1941. doi:<https://dx.doi.org/10.1097/QAD.0000000000002654>

100. Zhou Y, Lin YF, Meng X, et al. Anal human papillomavirus among men who have sex with men in three metropolitan cities in southern China: implications for HPV vaccination. *Vaccine*. 2020;38(13):2849-2858. doi:<https://dx.doi.org/10.1016/j.vaccine.2020.02.009>

101. Mendez-Martinez R, Maldonado-Frias S, Vazquez-Vega S, et al. High prevalent human papillomavirus infections of the oral cavity of asymptomatic HIV-positive men. *BMC Infectious Diseases*. 2020;20(1):27. doi:<https://dx.doi.org/10.1186/s12879-019-4677-9>

102. Jalil EM, Wilson EC, Monteiro L, et al. High prevalence of anal high-risk HPV infection among transwomen: estimates from a Brazilian RDS study. *Journal of the International AIDS Society*. 2021;24(3):e25691. doi:<https://dx.doi.org/10.1002/jia2.25691>

103. Alberts CJ, Heard I, Canestri A, et al. Incidence and Clearance of Anal Human Papillomavirus (HPV)-16 and HPV-18 Infection, and Their Determinants, Among Human Immunodeficiency Virus-Infected Men Who Have Sex With Men in France. *The Journal of infectious diseases*. 2020;221(9):1488-1493. doi:<https://dx.doi.org/10.1093/infdis/jiz623>

104. Biala M, Zalewska M, Szetela B, Gasiorowski J, Leszczyszyn J, Inglot M. Prevalence and genotype distribution of human papillomavirus infection among HIV-infected men who have sex with men living in Lower Silesia, Poland. *Postepy Dermatologii i Alergologii*. 2022;39(6):1128-1133. doi:<https://dx.doi.org/10.5114/ada.2022.122607>

105. Borena W, Kruis S, Kitchen M, et al. Anal Ureaplasma spp. positivity among HIV positive men who have sex with men may be associated with high-risk-type HPV infections. *International Journal of Infectious Diseases*. 2019;84:75-79. doi:<https://dx.doi.org/10.1016/j.ijid.2019.04.025>

106. Bruzzesi E, Galli L, Poli A, et al. Prevalence and Risk Factors of Anal HPV Infection in MSM Living With HIV: Identifying the Target Groups to Prioritize for Immunization. *Journal of Acquired Immune Deficiency Syndromes*. 2022;91(2):226-231. doi:<https://dx.doi.org/10.1097/QAI.0000000000003057>

107. Callejo A, Del Mar Molina M, Dinares MC, et al. Exploratory study of an oral screening dysplasia program for HIV-infected men who have sex with men. *AIDS*. 2022;36(10):1383-1391. doi:<https://dx.doi.org/10.1097/QAD.0000000000003287>

108. Combes J-D, Heard H, Poizot-Martin I, et al. Prevalence and Risk Factors for Anal Human Papillomavirus Infection in Human Immunodeficiency Virus-Positive Men Who Have Sex with Men. *Journal of Infectious Diseases*. 2018;217(10):1535-1543. doi:<https://dx.doi.org/10.1093/infdis/jiy059>

109. Dona MG, Benevolo M, Latini A, et al. Anal Cytological Lesions and HPV Infection in Individuals at Increased Risk for Anal Cancer. *Cancer Cytopathology*. 2018;126(7):461-470. doi:<https://dx.doi.org/10.1002/cncy.22003>

110. Giuliani M, Latini A, Colafigli M, et al. Vaccine-preventable anal infections by human papillomavirus among HIV-infected men who have sex with men. *Future Microbiology*. 2018;13(13):1463-1472. doi:<https://dx.doi.org/10.2217/fmb-2018-0149>

111. Hidalgo-Tenorio C, Gil-Anguita C, Lopez Ruz MA, Omar M, Lopez-Hidalgo J, Pasquau J. ART is key to clearing oncogenic HPV genotypes (HR-HPV) in anal mucosa of HIV-positive MSM. *PLoS ONE*. 2019;14(10):e0224183. doi:<https://dx.doi.org/10.1371/journal.pone.0224183>

112. Hidalgo-Tenorio C, García-Martínez CM, Pasquau J, et al. Risk factors for≥ high-grade anal intraepithelial lesions in MSM living with HIV and the response to topical and surgical treatments. *PLoS One*. 2021;16(2):e0245870.

113. Marra E, Siegenbeek Van Heukelom ML, Leeman A, et al. Virological and serological predictors of anal high-grade squamous intraepithelial lesions among human immunodeficiency virus-positive men who have sex with men. *Clinical Infectious Diseases*. 2019;68(8):1376-1387. doi:<https://dx.doi.org/10.1093/cid/ciy719>

114. Nemcova J, Riegert J, Cerna K, et al. Prevalence of oral and anal human papillomavirus infection in Czech predominantly HIV-positive men having sex with men - data from a previously unreported population. *International Journal of STD and AIDS*. 2022;33(12):1054-1064. doi:<https://dx.doi.org/10.1177/09564624221123869>

115. Parisi SG, Basso M, Scaggiante R, et al. Oral and anal high-risk human papilloma virus infection in HIV-positive men who have sex with men over a 24-month longitudinal study: complexity and vaccine implications. *BMC public health*. 2019;19(1):645. doi:<https://dx.doi.org/10.1186/s12889-019-7004-x>

116. Szabo E, Kosa C, Babarczi E, et al. Prevalence of Anal Human Papillomavirus Infection in Hungarian Men Who Have Sex with Men. *Pathology and Oncology Research*. 2018;24(3):671-677. doi:<https://dx.doi.org/10.1007/s12253-017-0292-5>

117. Ucciferri C, Tamburro M, Falasca K, Sammarco ML, Ripabelli G, Vecchiet J. Prevalence of anal, oral, penile and urethral human papillomavirus in HIV infected and HIV uninfected men who have sex with men. *Journal of Medical Virology*. 2018;90(2):358-366. doi:<https://dx.doi.org/10.1002/jmv.24943>

118. Ong JJ, Walker S, Grulich A, et al. Incidence, Clearance, and Persistence of Anal Human Papillomavirus in Men Who Have Sex with Men Living with Human Immunodeficiency Virus: Implications for Human Papillomavirus Vaccination. *Sexually Transmitted Diseases*. 2019;46(4):229-233. doi:<https://dx.doi.org/10.1097/OLQ.0000000000000958>

119. Poynten IM, Jin F, Garland SM, et al. HIV, Immune Dysfunction, and the Natural History of Anal High-Risk Human Papillomavirus Infection in Gay and Bisexual Men. *Journal of Infectious Diseases*. 2021;224(2):246-257. doi:<https://dx.doi.org/10.1093/infdis/jiaa723>

120. Bhardwaj S, Gaisa M, Sigel K, Liu Y. HPV-associated Anal Intraepithelial Neoplasia in <35-year-old HIV-infected Men Who Have Sex with Men. *Journal of Lower Genital Tract Disease*. 2022;26(2 SUPPL 1):S1. Scientific Meeting on Anogenital and HPV-Related Diseases, ASCCP 2022. San Diego, CA United States. doi:<https://dx.doi.org/10.1097/LGT.0000000000000670>

121. Harfouch O, Eyasu R, Cover AA, et al. RISK FACTORS FOR ANAL DYSPLASIA AND LINKAGE TO HRA IN TRANSGENDER WOMEN. *Sexual Health*. 2022;19(2):xiii. 2022 Scientific Meeting of the International Anal Neoplasia Society's, IANS 2022. New York City, NY United States. doi:<https://dx.doi.org/10.1071/SHv19n2abs>

122. Gupta R, Hussain S, Hariprasad R, et al. Concurrent Cervical and Anal High-Risk Human Papillomavirus Infection in Women Living with HIV: An Observational Case-Control Study. *Journal of Acquired Immune Deficiency Syndromes*. 2022;91(3):319-324. doi:<https://dx.doi.org/10.1097/QAI.0000000000003064>

123. Rodrigues LLS, Pilotto JH, Martinelli KG, et al. Diversity of Anal HPV and Non-HPV Sexually Transmitted Infections and Concordance with Genital Infections in HIV-Infected and HIV-Uninfected Women in the Tapajos Region, Amazon, Brazil. *Viruses*. 2023;15(6)doi:<https://dx.doi.org/10.3390/v15061328>

124. Dona MG, Giuliani M, Rollo F, et al. Incidence and clearance of anal high-risk Human Papillomavirus infection and their risk factors in men who have sex with men living with HIV. *Scientific reports*. 2022;12(1):184. doi:<https://dx.doi.org/10.1038/s41598-021-03913-5>

125. Poynten IM, Jin F, Molano M, et al. Possible Reactivation of Latent Anal Human Papillomavirus Associated with Markers of Immune Dysfunction in Gay and Bisexual Men. *Cancer Epidemiology Biomarkers and Prevention*. 2022;31(5):1052-1057. doi:<https://dx.doi.org/10.1158/1055-9965.EPI-21-1346>

126. Castillejos-Garcia I, Ramirez-Amador VA, Carrillo-Garcia A, Garcia-Carranca A, Lizano M, Anaya-Saavedra G. Type-specific persistence and clearance rates of HPV genotypes in the oral and oropharyngeal mucosa in an HIV/AIDS cohort. *Journal of Oral Pathology and Medicine*. 2018;47(4):396-402. doi:<https://dx.doi.org/10.1111/jop.12687>

127. Goncalves HM, Silva J, Pintado Maury I, et al. The prevalence and risk-factors of oral HPV DNA detection among HIV-infected men between men who have sex with men and heterosexual men. *Infectious Diseases*. 2021;53(1):19-30. doi:<https://dx.doi.org/10.1080/23744235.2020.1811373>

128. Riddell J, Brouwer AF, Walline HM, et al. Oral human papillomavirus prevalence, persistence, and risk-factors in HIV-positive and HIV-negative adults. *Tumour Virus Research*. 2022;13:200237. doi:<https://dx.doi.org/10.1016/j.tvr.2022.200237>

129. Gheit T, Rollo F, Brancaccio RN, et al. Oral infection by mucosal and cutaneous human papillomaviruses in men who have sex with men from the ohmar study. *Viruses*. 2020;12(8):v12080899. doi:<https://dx.doi.org/10.3390/v12080899>

130. Oliver SE, Gorbach PM, Gratzer B, et al. Risk Factors for Oral Human Papillomavirus Infection among Young Men Who Have Sex with Men - 2 Cities, United States, 2012-2014. *Sexually Transmitted Diseases*. 2018;45(10):660-665. doi:<https://dx.doi.org/10.1097/OLQ.0000000000000845>

131. Rollo F, Latini A, Benevolo M, et al. Concurrent and concordant anal and oral human papillomavirus infections are not associated with sexual behavior in at-risk males. *Pathogens*. 2021;10(10):1254. doi:<https://dx.doi.org/10.3390/pathogens10101254>

132. Tsikis S, Hoefer L, Bethimoutis G, et al. Risk factors, prevalence, and site concordance of human papillomavirus in high-risk Greek men. *European journal of cancer prevention : the official journal of the European Cancer Prevention Organisation (ECP)*. 2018;27(5):514-520. doi:<https://dx.doi.org/10.1097/CEJ.0000000000000366>

133. Giuliani M, Rollo F, Vescio MF, et al. Oral human papillomavirus infection in HIV-infected and HIV-uninfected MSM: The OHMAR prospective cohort study. *Sexually Transmitted Infections*. 2020;96(7):528-536. doi:<https://dx.doi.org/10.1136/sextrans-2019-054301>

134. Bouassa RSM, Belec L, Gubavu C, et al. High Prevalence of Anal and Oral High-Risk Human Papillomavirus in Human Immunodeficiency Virus-Uninfected French Men Who Have Sex with Men and Use Preexposure Prophylaxis. *Open Forum Infectious Diseases*. 2019;6(9):ofz291. doi:<https://dx.doi.org/10.1093/ofid/ofz291>

135. Camargo M, Del Rio-Ospina L, Soto-De Leon SC, et al. Association of HIV status with infection by multiple HPV types. *Tropical Medicine and International Health*. 2018;23(11):1259-1268. doi:<https://dx.doi.org/10.1111/tmi.13142>

136. Gupta R, Hussain S, Hariprasad R, et al. High Prevalence of Cervical High-Grade Lesions and High-Risk Human Papillomavirus Infections in Women Living with HIV: A Case for Prioritizing Cervical Screening in This Vulnerable Group. *Acta Cytologica*. 2022;66(6):496-506. doi:<https://dx.doi.org/10.1159/000525340>

137. Lewis S, Mphande M, Chibwana F, et al. Association of HIV status and treatment characteristics with VIA screening outcomes in Malawi: A retrospective analysis. *PLoS ONE*. 2022;17(1 1):e0262904. doi:<https://dx.doi.org/10.1371/journal.pone.0262904>

138. Nakisige C, Adams SV, Namirembe C, et al. Multiple High-Risk HPV Types Contribute to Cervical Dysplasia in Ugandan Women Living with HIV on Antiretroviral Therapy. *Journal of Acquired Immune Deficiency Syndromes*. 2022;90(3):333-342. doi:<https://dx.doi.org/10.1097/QAI.0000000000002941>

139. Ndizeye Z, Vanden Broeck D, Lebelo RL, Bogers J, Benoy I, Van Geertruyden J-P. Prevalence and genotype-specific distribution of human papillomavirus in Burundi according to HIV status and urban or rural residence and its implications for control. *PloS one*. 2019;14(6):e0209303. doi:<https://dx.doi.org/10.1371/journal.pone.0209303>

140. Ogu CO, Achukwu PU, Nkwo PO. Prevalence and Risk Factors of Cervical Dysplasia among Human Immunodeficiency Virus Sero-Positive Females on Highly Active Antiretroviral Therapy in Enugu, Southeastern, Nigeria. *Asian Pacific journal of cancer prevention : APJCP*. 2019;20(10):2987-2994. doi:<https://dx.doi.org/10.31557/APJCP.2019.20.10.2987>

141. Ogunsowo K, Akadri A, Odelola O, Adefuye P, Shorunmu T, Ebili H. Prevalence and predictors of squamous intraepithelial lesions in human immunodeficiency virus positive women in Sagamu, southwest Nigeria. *Journal of Public Health in Africa*. 2022;13(3):1858. doi:<https://dx.doi.org/10.4081/jphia.2022.1858>

142. Massad LS, Xie X, Minkoff H, et al. Longitudinal assessment of abnormal Papanicolaou test rates among women with HIV. *AIDS (London, England)*. 2020;34(1):73-80. doi:<https://dx.doi.org/10.1097/QAD.0000000000002388>

143. Ye Y, Burkholder GA, Wiener HW, et al. Comorbidities associated with HPV infection among people living with HIV-1 in the southeastern US: A retrospective clinical cohort study. *BMC Infectious Diseases*. 2020;20(1):144. doi:<https://dx.doi.org/10.1186/s12879-020-4822-5>

144. Floridia M, Masuelli G, Tassis B, et al. Minimal prevalence of HPV vaccination and common occurrence of high-risk HPV types in pregnant women with HIV: data from a national study in Italy. *European Journal of Clinical Microbiology & Infectious Diseases*. 2022:1-5.

145. Fusco FM, Vichi F, Bisanzi S, et al. HPV infection and pre-neoplastic cervical lesions among 321 HIV+ women in Florence, Italy, 2006-2016: prevalence and associated factors. *The new microbiologica*. 2018;41(4):268-273.

146. Menon S, Rossi R, Benoy I, Bogers JP, van den Broeck D. Human papilloma virus infection in HIV-infected women in Belgium: implications for prophylactic vaccines within this subpopulation. *European journal of cancer prevention : the official journal of the European Cancer Prevention Organisation (ECP)*. 2018;27(1):46-53. doi:<https://dx.doi.org/10.1097/CEJ.0000000000000271>

147. Cambou MC, Levi JE, Lake JE, et al. Time trend analysis of cervical high-risk human papillomavirus (HPV) in HIV-infected women in an urban cohort from Rio de Janeiro, Brazil: The rise of non-16/18 HPV. *International Journal of Infectious Diseases*. 2015;41:17-20. doi:<https://dx.doi.org/10.1016/j.ijid.2015.10.017>

148. Joshi S, Muwonge R, Kulkarni V, et al. Can we increase the cervical cancer screening interval with an HPV test for women living with HIV? Results of a cohort study from Maharashtra, India. *International journal of cancer*. 2023;152(2):249-258. doi:<https://dx.doi.org/10.1002/ijc.34221>

149. Prathima S, Sarojini, Latha B, Ashakiran TR. Study of Prevalence of Abnormal Pap Smear and its Associated Risk Factors in HIV Positive Women: A Cross-sectional Study. *Journal of Obstetrics and Gynecology of India*. 2022;72(Supplement 1):255-261. doi:<https://dx.doi.org/10.1007/s13224-021-01533-7>

150. Shin SS, Carpenter CL, Ekstrand ML, et al. Cervical cancer awareness and presence of abnormal cytology among HIV-infected women on antiretroviral therapy in rural Andhra Pradesh, India. *International journal of STD & AIDS*. 2019;30(6):586-595. doi:<https://dx.doi.org/10.1177/0956462419825950>

151. Vasnik GK, Jain G, Husainy FA, Bansal V. Correlation of Cervical Squamous Intraepithelial Lesions with Human Papillomavirus in Women Infected with the Human Immunodeficiency Virus. *Indian Journal of Gynecologic Oncology*. 2019;17(1):6. doi:<https://dx.doi.org/10.1007/s40944-018-0251-4>

152. Ouladlahsen A, Fayssel N, Bensghir R, et al. Human papillomavirus among women living with human immunodeficiency virus in Morocco A prospective cross-sectional study. *Journal of Infection in Developing Countries*. 2018;12(6):477-484. doi:<https://dx.doi.org/10.3855/jidc.9711>

153. Smeltzer S, Yu X, Schmeler K, Levison J. Abnormal Vaginal Pap Test After Hysterectomy in Human Immunodeficiency Virus-Infected Women. *Obstetrics and gynecology*. 2016;127(Supplement 1):4S. doi:<https://dx.doi.org/10.1097/01.AOG.0000483629.84944.db>

154. Clark E, Chen L, Dong Y, et al. Veteran Women Living With Human Immunodeficiency Virus Have Increased Risk of Human Papillomavirus (HPV)-Associated Genital Tract Cancers. *Clinical infectious diseases : an official publication of the Infectious Diseases Society of America*. 2021;72(9):e359-e366. doi:<https://dx.doi.org/10.1093/cid/ciaa1162>

155. Rositch AF, Levinson K, Suneja G, et al. Epidemiology of Cervical Adenocarcinoma and Squamous Cell Carcinoma Among Women Living With Human Immunodeficiency Virus Compared With the General Population in the United States. *Clinical infectious diseases : an official publication of the Infectious Diseases Society of America*. 2022;74(5):814-820. Comment in: Clin Infect Dis. 2022 Mar 23;74(6):1124-1125 PMID: 34427653 [<https://www.ncbi.nlm.nih.gov/pubmed/34427653>]. doi:<https://dx.doi.org/10.1093/cid/ciab561>

156. Chaussade H, Le Marec F, Coureau G, et al. Incidence of lung and human papilloma virus-associated malignancies in HIV-infected patients. *AIDS (London, England)*. 2022;36(5):665-673. doi:<https://dx.doi.org/10.1097/QAD.0000000000003152>

157. Khalil IA, Mpunga T, Wei F, et al. Age-specific burden of cervical cancer associated with HIV: A global analysis with a focus on sub-Saharan Africa. *International Journal of Cancer*. 2022;150(5):761-772. doi:<https://dx.doi.org/10.1002/ijc.33841>

158. Ortiz AP, Engels EA, Nogueras-Gonzalez GM, et al. Disparities in human papillomavirus-related cancer incidence and survival among human immunodeficiency virus-infected Hispanics living in the United States. *Cancer*. 2018;124(23):4520-4528. doi:<https://dx.doi.org/10.1002/cncr.31702>

159. Zhou G, Koroukian SM, Navale SM, et al. Cancer burden in women with HIV on Medicaid_ A nationwide analysis. *Women's Health*. 2023;19doi:<https://dx.doi.org/10.1177/17455057231170061>

160. Furukawa S, Uota S, Yamana T, et al. Distribution of Human Papillomavirus Genotype in Anal Condyloma Acuminatum among Japanese Men: The Higher Prevalence of High Risk Human Papillomavirus in Men Who Have Sex with Men with HIV Infection. *AIDS Research and Human Retroviruses*. 2018;34(4):375-381. doi:<https://dx.doi.org/10.1089/aid.2017.0197>

161. Geba M, Cardenas B, Williams B, et al. Prevalence and Predictors of High-Grade Anal Dysplasia in People With HIV in One Southeastern Ryan White HIV/AIDS Program Clinic. *Open Forum Infectious Diseases*. 2022;9(11):ofac556. doi:<https://dx.doi.org/10.1093/ofid/ofac556>

162. Finkelman B, Weinberg S, Obeng R, Sun L, Liao J, Yang GY. HIV infection and low CD4 count are associated with faster progression of hpvmediated anal squamous intraepithelial lesions: A large clinicopathologic cohort study. *Laboratory Investigation*. 2021;101(SUPPL 1):410-411. 110th Annual Meeting of the United States and Canadian Academy of Pathology. Virtual. doi:<https://dx.doi.org/10.1038/s41374-021-00556-y>

163. Brogden DR, Lupi ME, Kontovounisios C, Tekkis P, Mills SC. IS LOCAL EXCISION ALONE FOR EARLY ANAL SQUAMOUS CELL CARCINOMA (ASCC) EFFICACIOUS IN PEOPLE LIVING WITH HIV (PLWH)? CSIRO PUBLISHING UNIPARK, BLDG 1, LEVEL 1, 195 WELLINGTON RD, LOCKED BAG 10 …; 2022:XXXVII-XXXVII.

164. Cerejeira A, Cunha S, Coelho R, et al. Perianal warts as a risk marker for anal high-risk-human papillomavirus (HPV) detection and HPV-associated diseases. *Journal of the European Academy of Dermatology and Venereology*. 2020;34(11):2613-2619. doi:<https://dx.doi.org/10.1111/jdv.16834>

165. Waters A, Dorsey K, Allston A, Woods A, Furness B, Doshi R. Risk Factors for Human Papillomavirus-Associated Cancers Among People Living with HIV in Washington, District of Columbia. *AIDS Res Hum Retroviruses*. 2023;39(4):195-203. doi:10.1089/AID.2022.0128.

166. Picard A, Arowas L, Piroth L, Heard I, Ferry T. Head and neck squamous cell carcinoma in people living with HIV in France. *Medecine et Maladies Infectieuses*. 2018;48(8):503-508. Carcinomes epidermoides de la sphere ORL chez les patients vivant avec le VIH en France. doi:<https://dx.doi.org/10.1016/j.medmal.2018.05.002>
